# *Mycobacterium tuberculosis* VadK is required for the regulation of the methylcitrate cycle and virulence

**DOI:** 10.1038/s44319-026-00818-0

**Published:** 2026-06-11

**Authors:** Jordan Pascoe, Jane Newcombe, Jessica Mendoza, Shalini Birua, Tom A Mendum, Kushi Anand, Albel Singh, Amitava Sinha, Kadamba Papavinasasundaram, Apoorva Bhatt, Gerald Larrouy-Maumus, Amit Singh, Celia W Goulding, Dany J V Beste

**Affiliations:** 1 Discipline of Microbes, Infection and Immunity, University of Surrey, Guildford, GU2 7XH UK; 2 Department of Molecular Biology & Biochemistry, University of California Irvine, Irvine, CA 92697 USA; 3 Department of Microbiology and Cell Biology, Indian Institute of Science, Bangalore, Karnataka 560012 India; 4 Department of Life Sciences, Kristu Jayanti University, Bangalore, India; 5 School of Biosciences, University of Birmingham, Edgbaston, Birmingham B15 2TT UK; 6 Department of Microbiology, University of Massachusetts Chan Medical School, Worcester, MA 01605 USA; 7 Centre for Bacterial Resistance Biology, Department of Life Sciences, Faculty of Natural Sciences, Imperial College London, London, SW7 2AZ UK; 8 Department of Pharmaceutical Sciences, University of California Irvine, Irvine, CA 92697 USA

**Keywords:** Metabolism, Microbiology, Virology & Host Pathogen Interaction, Signal Transduction

## Abstract

The evolution of new enzymatic functions is constrained and guided by the architecture of an organism’s metabolic and regulatory networks and environmental constraints. Here, we identify a kinase that has evolved from pyruvate phosphate dikinase. Using biochemical and systems-level analyses, we show that this enzyme, encoded by *rv1127c* in *Mycobacterium tuberculosis* (*Mtb*), has diverged from its ancestral role in central carbon metabolism to function as a histidine kinase in pathogenic mycobacteria and related species. We designate this enzyme Virulence Associated DiKinase (VadK), reflecting its ability to autophosphorylate and its role in virulence. VadK is essential for the utilization of carbon sources critical for survival within the host and to cause tuberculosis (TB) in murine models. Furthermore, VadK interacts with enzymes of the methylcitrate cycle, and ^13^C-tracer experiments demonstrates that it fine-tunes flux through this pathway, with elevated flux proving growth limiting. Together, these findings identify VadK as a regulatory kinase that integrates metabolic control with virulence in *Mtb*, revealing a new facet of metabolic regulation in bacterial pathogenesis and a potential target for therapeutic intervention.

## Synopsis

*Mycobacterium tuberculosis* (*Mtb*) VadK is a non-canonical histidine kinase that modulates the methylcitrate cycle to fine tune metabolism, redox balance and bioenergetics. Loss of VadK dysregulates central carbon metabolism and abolishes virulence in murine tuberculosis models.

- VadK evolved from pyruvate phosphate dikinase, that directly regulates the methylcitrate cycle.
- Loss of VadK causes methylcitrate cycle hyperflux, disrupting central carbon metabolism, redox balance and bioenergetics.
- VadK is essential for *Mtb* virulence in murine models of tuberculosis, with Δ*vadK* severely attenuated for survival in lungs and spleen.
- VadK orthologues are absent from humans, highlighting VadK as a pathogen-specific drug target.

## Abstract (web summary)

*Mycobacterium tuberculosis* VadK is a non-canonical histidine kinase that modulates the methylcitrate cycle to fine tune metabolism, redox balance and bioenergetics. Loss of VadK dysregulates central carbon metabolism and abolishes virulence in murine tuberculosis models.

## Graphical abstract

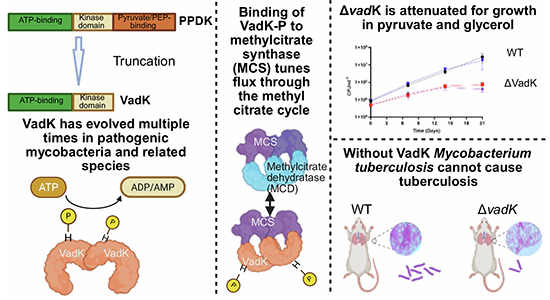

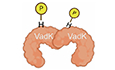

## Introduction

*Mycobacterium tuberculosis* (*Mtb*), the causative agent of human tuberculosis (TB), is still a formidable human pathogen that, despite decades of research and widely available treatments, remains a leading cause of death globally (World Health, 2024). Although the anti-TB drug pipeline is much healthier than it has been for decades, there remains an urgent need to develop shorter, less toxic treatments that can target antibiotic-resistant strains of *Mtb*. Central carbon metabolism (CCM) has been recognised as a promising target for anti-TB therapy. This connection is illustrated by bedaquiline, a clinically approved ATP synthase inhibitor, which disrupts energy production linked to CCM and exhibits potent bactericidal activity (Lakshmanan and Xavier, 2013). CCM likely harbours many other unidentified targets with critical roles in *Mtb’s* survival.

Our work on *Mtb* metabolism previously identified that enzymes of the anaplerotic (ANA) node of CCM have unique and essential roles in controlling the flux of carbon through *Mtb* (Basu et al, 2018; Burley et al, 2021). The *Mtb* ANA node consists of the enzymes pyruvate carboxylase (PCA), phosphoenolpyruvate (PEP) carboxykinase (PCK), malic enzyme (MEZ) and Rv1127c that is annotated as pyruvate phosphate dikinase (PPDK). Whilst humans have orthologues of the enzymes PCK, MEZ and PCA, the absence of PPDK in vertebrates identifies this enzyme as a promising anti-microbial drug target.

By measuring the metabolic flux profile of *Mtb* in a macrophage model of TB, we discovered that in the absence of Rv1127c, metabolism was dysregulated, and its growth inhibited (Basu et al, 2018). We also showed that Rv1127c was essential for* Mtb* growth in media containing cholesterol, or its breakdown product propionate, even in the presence of an additional growth-permissive carbon source (Basu et al, 2018). Furthermore, we showed that ∆*rv1127c* is more susceptible to bedaquiline (BDQ) than wild-type (WT) *Mtb* (Mackenzie et al, 2020), and therefore drugs targeting Rv1127c may synergise with BDQ, which is the backbone of the oral, shortened treatments for antibiotic-resistant TB.

**Figure EV1:**
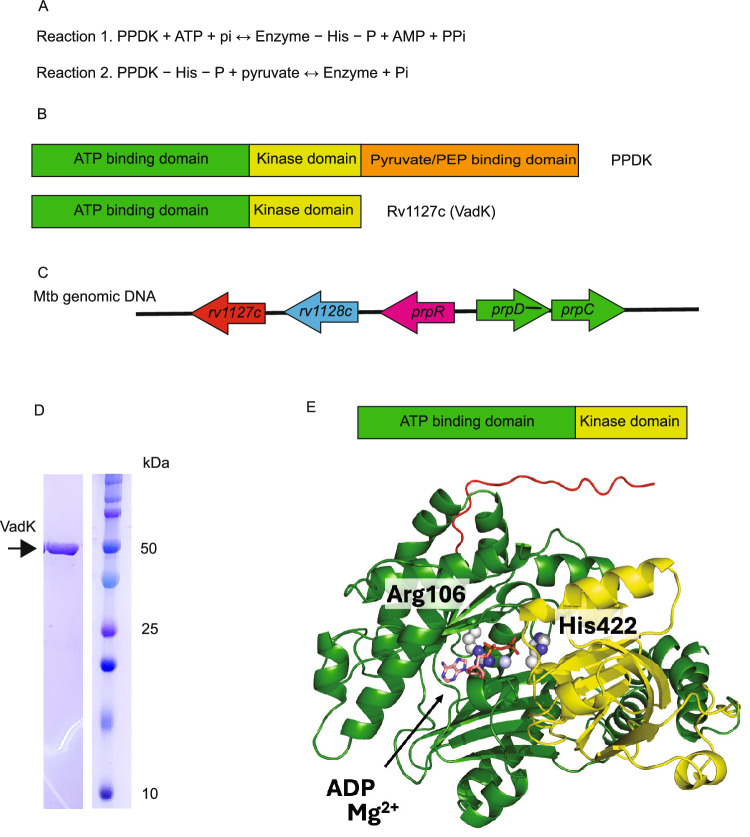
Reaction of PPDK and its structural comparison with VadK. (**A**) The two-step reaction of canonical PPDK. (**B**) VadK is missing the PPDK pyruvate binding domain. (**C**) *vadK* is in close proximity to genes of the methylcitrate cycle. (**C**) *Mtb* genomic location of *vadK*, (**D**) SDS-PAGE gel of purified VadK and (**E**) VadK complexed with ADP and Mg^2+^ structure prediction by AlphaFold3 (Abramson et al, 2024). The N-terminal 1-17 residues, ATP binding and kinase domains are coloured in red, green and yellow, respectively. ADP and Mg^2+^ are shown in stick (carbons, salmon) and sphere (light blue), respectively. The catalytic histidine, His422, and ATP-binding Arg106 are highlighted in spheres (carbons, white). Data Information: In (**E**), the structure was predicted with high confidence using AlphaFold3 (Abramson et al, 2024) with prediction statistics ipTM/pTM scores of 0.97/0.79 and visualised in PyMOL.

In many other organisms, PPDK is a critical enzyme. In bacteria and protozoa, it has roles in glycolysis and ATP production via substrate-level phosphorylation (Koendjbiharie et al, 2020), whilst in C4-plants it is critical for carbon cycling during photosynthesis (Om et al, 2022). Structurally, PPDK is an ATP-grasp protein composed of three distinct domains: an N-terminal ATP-binding domain, a central kinase domain (containing a catalytic histidine) and a C-terminal pyruvate/PEP binding domain. PPDK can catalyse a reversible three-step reaction sequence at two separate active sites that interconverts ATP, phosphate and pyruvate to phosphoenolpyruvate (PEP), pyrophosphate and AMP (Fig. EV1A).

In contrast to its roles in other organisms, we have previously demonstrated using ^13^C-Metabolic Flux Analysis that in *Mtb* there was no flux through the PPDK reaction (Borah et al, 2021). Furthermore, *rv1127c* is truncated, encoding only the PPDK N-terminal ATP-binding domain and the central catalytic histidine kinase domain but not the pyruvate/PEP binding domain (Fig. EV1B). The *Mtb* genome contains no genes encoding this domain that could form a complex with Rv1127c and so restore its canonical function (Borah et al, 2021). We therefore conclude that Rv1127c cannot function as a canonical PPDK.

Although *rv1127c* is not a functional PPDK, high-throughput mutagenesis screens conducted by our group and others predicted that *rv1127c* is essential under a variety of conditions, including in a chemostat model (Beste et al, 2009), cholesterol media (Griffin et al, 2012), and murine models of TB (Meade et al, 2023; Smith et al, 2022).

Functionally related genes are frequently located near each other in the genome of prokaryotes (Jacob and Monod, 1961). The genomic loci of *rv1127c* is adjacent to genes that encode essential enzymes of the methylcitrate cycle (MCC) (methylcitrate synthase (MCS and Rv1131c) and methylcitrate dehydratase (MCD and Rv1130c) and their transcriptional regulator (PrpR and Rv1129c) (Fig. EV1C) that like Rv1127c, are essential for the metabolism of cholesterol and propionate (Griffin et al, 2012), suggesting that Rv1127c may perform an unexpected role in the MCC. The MCC is one of three approaches that *Mtb* can use to metabolise the potentially toxic propionyl-CoA that is derived from the catabolism of host-derived sterols, odd-chain fatty acids and branched-chain amino acids, substrates that are present and utilised in vivo (Lee et al, 2013). The MCC metabolises propionyl-CoA to pyruvate via the potentially toxic intermediates, 2-methylcitrate and 2-methylisocitrate (Russell et al, 2010). In the presence of vitamin B_12_, the MCC is transcriptionally repressed (Pawełczyk et al, 2021), and *Mtb* uses the alternative B_12_-dependent methylmalonyl pathway to metabolise propionyl-CoA to succinyl-CoA. *Mtb* can also incorporate propionyl-CoA into its cell wall, including the virulence-associated lipids phthiocerol dimycocerosates (PDIM) and the trehalose ester family (sulfolipid-1 (SL-1), diacyltrehalose, triacyltrehalose, and polyacyltrehalose), which are synthesised from malonyl-CoA (produced from acetyl CoA) and methylmalonyl-CoA (produced from propionyl-CoA) (Lee et al, 2013). As these pathways are important for *Mtb* virulence and to prevent the buildup of potentially toxic metabolites, it is vital to *Mtb* that these pathways are rigorously regulated.

We previously demonstrated that growth attenuation of Δ*rv1127c* in propionate is rescued by the addition of either vitamin B_12_ or acetate (Basu et al, 2018), data that phenocopies *Mtb* mutants lacking MCC enzymes (Lee et al, 2013), thus supporting an unexpected role for Rv1127c in the MCC (Borah et al, 2021).

Here we demonstrate that deletion of *rv1127c* results in dysregulated CCM, disruption in both redox balance and in bioenergetics, when grown on propionate-containing, or yielding medium, and reduced survival and virulence of *Mtb* in two different murine models of TB. Our results confirm the importance of this protein in the life cycle of *Mtb*, and therefore, we herein rename this protein Virulence Associated Dikinase (VadK). We confirm that, despite being truncated, Rv1127c is a functional histidine kinase that fine-tunes the MCC by interacting with the enzymes of this pathway. The discovery of a unique, non-canonical histidine phosphotransfer system that has evolved from the CCM enzyme PPDK in pathogenic Mycobacteria and other related genera enhances our understanding of the regulation of metabolism and has implications for understanding the role of the MCC in *Mtb* growth and virulence.

## Results

### VadK is essential for *Mtb* to cause tuberculosis

**Figure EV2:**
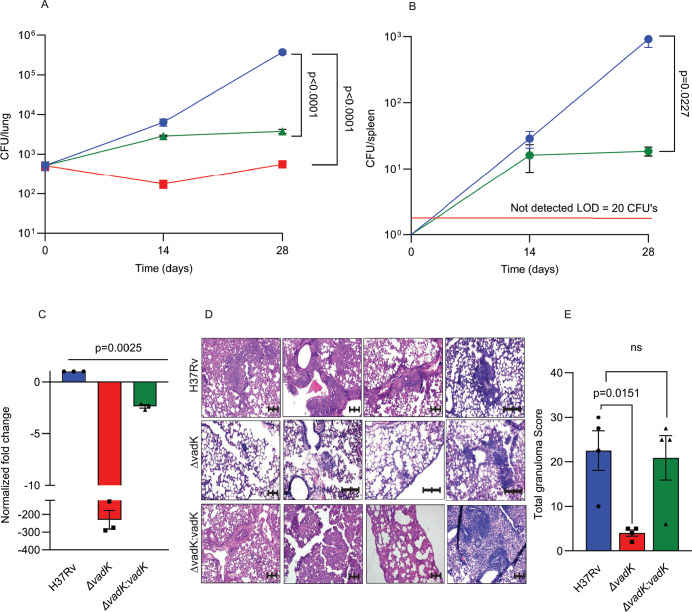
VadK is required for *Mtb* to cause tuberculosis in BALB/c mice. BALB/c mice (*n* = 6) were aerosol infected with WT (blue), *∆vadK* (red) and *∆vadK:vadK* (green) and the bacterial load in the (**A**) lungs and (**B**) spleen was measured at day 14 and day 28. *∆vadK* was not detected in the spleen, and the limit of detection is 20 CFU’s. (**C**) Total RNA was isolated from animal passaged logarithmically grown cells (*n* = 3) of WT H37Rv, *∆vadK* (red) and *∆vadK:vadK* (green) and expression of *vadK* was measured by real-time PCR. The results are from *n* = 3 biological replicates and are expressed as fold change as compared with WT expression ± SEM. (**D**) Lung sections were stained with HE after 28 days of infection and scored blindly by a pathologist using the method described (Kramnik and Beamer, 2016). The images show HE-stained (10 × magnification) from individual sections representative of four infected animals, and (**E**) the scores for animals (*n* = 4) in each group. Data information: In (**A**–**C**,** E**), data were presented as mean ± SEM. Comparison for determining statistical significance was made using an ordinary two-way ANOVA (Dunnett’s multiple comparison test) for (**A**), an unpaired *t*-test with Welch’s correction for (**B**, **C**) and an ordinary one-way ANOVA (Dunnett’s multiple comparison test) for (**E**). Scale bars in (**D**): 100 µm.

We previously demonstrated that Rv1127c (VadK) is essential for the intracellular growth of *Mtb* in macrophages, and this correlated with significant dysregulation of intraphagosomal bacterial metabolism, suggesting that VadK plays a critical role in bacterial survival within the host (Basu et al, 2018). To test this hypothesis, we used two murine models of TB, a low-dose aerosol infection of BALB/c mice and the Kramnik model (C3HeB/FeJ mice), which develop human-like granulomas (Kramnik et al, 2000). In accordance with our hypothesis, Δ*vadK* was severely attenuated and unable to grow in the lungs or disseminate into the spleen of BALB/c mice (Fig. EV2A,B). Although there was a significant difference between the growth of Δ*vadK* and Δ*vadK:vadK*, complementation was incomplete, suggesting that expression of *vad*K from the chromosomally distant *att*B site where we re-inserted it into the genome using pMV306 under its native promoter was insufficient. (Basu et al, 2018). Consistent with this, qRT-PCR analysis of RNA derived from bacteria isolated from murine lungs (Fig. EV2C) confirmed that the complemented strain expressed *vadK*, albeit at lower levels than the parental strain. Further, the histopathology data (Fig. EV2D,E) showed that whilst mice infected with ∆*vadK* had significantly less tissue damage, there were no significant differences between WT and *ΔvadK:vadK* infected mice, providing further evidence that the phenotype of Δ*vadK* was due to deletion of *vadK*.

**Figure 1:**
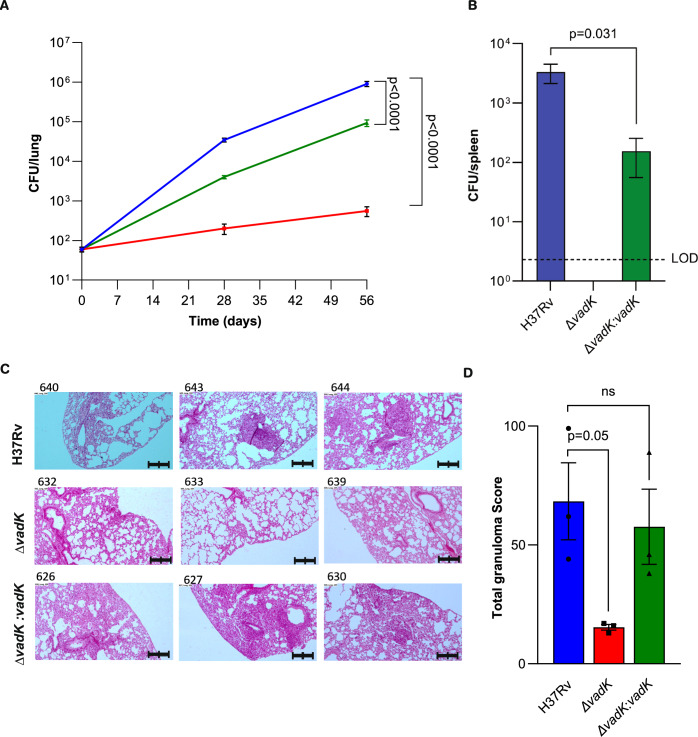
VadK is required for *Mtb* to cause tuberculosis in C3HeB/FeJ mice. C3HeB/FeJ mice (*n* = 8) were aerosol infected with WT (blue), *∆vadK* (red) and *∆vadK:vadK* (green). *Mtb* survival was measured in the (**A**) lungs at the indicated time points and (**B**) dissemination to the spleen after 56 days; *∆vadK* was not detected in the spleen. The limit of detection (LOD) is 20 CFU’s. (**C**) Lung sections were stained with haematoxylin and eosin (HE) after 56 days of infection and scored blindly by a pathologist using (Kramnik and Beamer, 2016). The images show HE-stained (10 × magnification) from individual lung sections representative of three infected animals/group (animal number indicated) and (**D**) the scores for animals in each group. Data information: In (**A**,** B**,** D**), data were presented as mean ± SEM. Comparison for determining statistical significance was made using an ordinary two-way ANOVA (Dunnet’s multiple Comparison test) for (**A**), ordinary one-way ANOVA (Dunnet’s multiple Comparison test) for (**B**) and an unpaired two-tailed *t*-test with Welch’s correction for (**D**). ns=not significant. Scale bars in (**C**): 100 µm. Source data are available online for this figure.

The bacterial burden in C3HeB/FeJ mice showed a similar result to the BALB/c model, again demonstrating that Δ*vadK* was unable to grow in either the lungs or disseminate to the spleen (Fig. 1A,B). Complementation restored bacterial burden in the lungs and dissemination to the spleen. Consistent with this, Δ*vadK* infection caused significantly less pathology (granuloma score,15.3) as compared with the parental (granuloma score, 68.3) and the complemented strain (granuloma score, 57.7) (Fig. 1C,D). In conclusion, the infection experiments showed that VadK is required for *Mtb* to cause disease in murine models of tuberculosis and thus is essential for virulence.

### VadK can autophosphorylate in vitro

**Figure 2:**
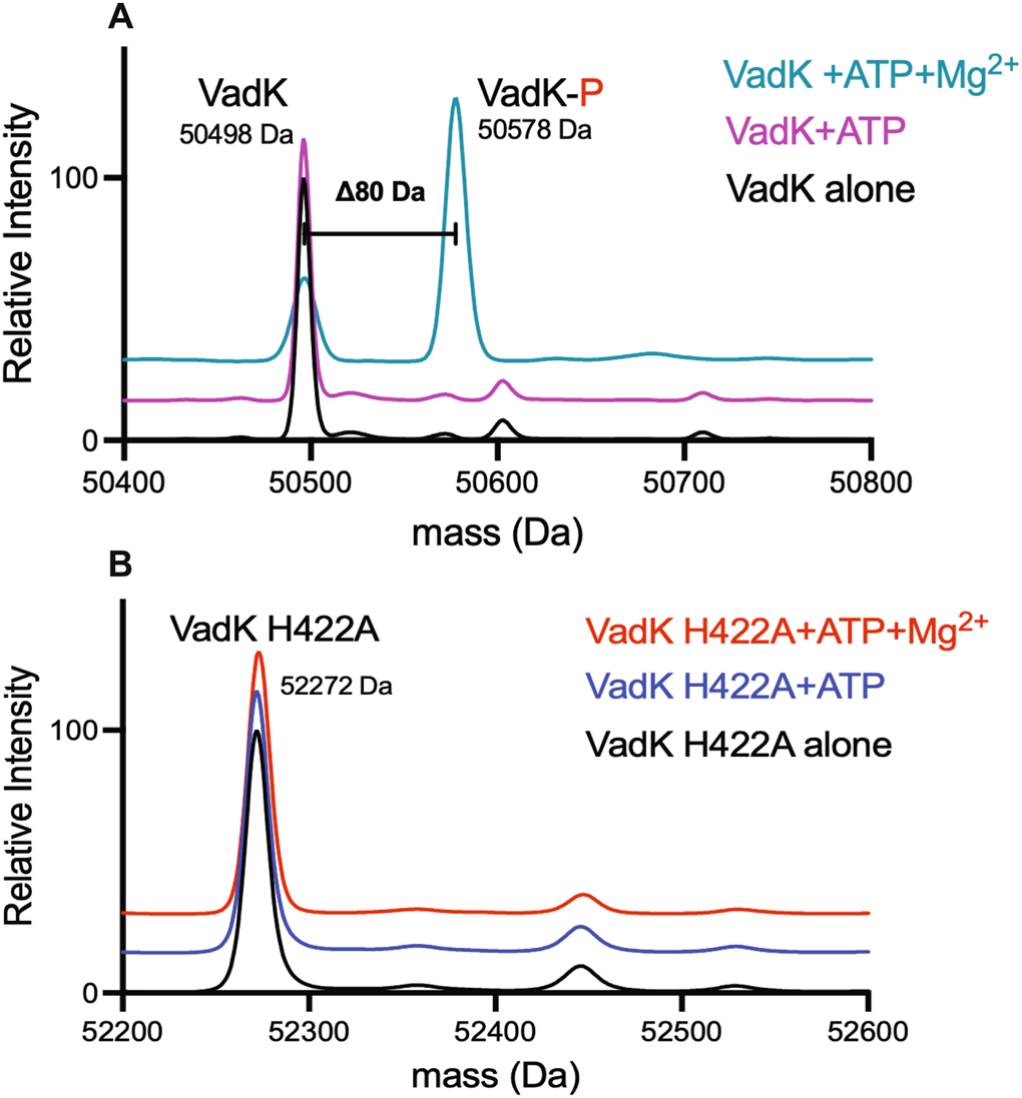
VadK-His can autophosphorylate. (**A**) MS of VadK alone (black line), with ATP and in the absence (purple line) and presence of Mg^2+^ (cyan line). A phosphorylation event (mass shift 80 Da, designated by VadK-P) only occurs for apo-VadK in the presence of ATP and Mg^2+^. (**B**) MS of un-phosphorylatable VadK^His422Ala^ alone (black line), with ATP and in the absence (blue line) and presence of Mg^2+^ (red line). No phosphorylation event is observed as there is no mass shift of ~80 Da in the presence of ATP and Mg^2+^. Data information: This is representative of at least triplicate biological experiments for each spectrum. The raw and deconvoluted data were deposited in the MassIVE repository (https://massive.ucsd.edu) under accession MSV000101031.

VadK consists of an N-terminal ATP-binding domain and a phospho-transfer histidine kinase domain. We predicted that VadK is an ATP-dependent (di)kinase that autophosphorylates its catalytic histidine. To test this, we purified recombinant VadK (Fig. EV1D) and tested its ability to autophosphorylate by measuring ATP-dependent VadK phosphorylation with and without Mg^2+^ (an essential co-factor of PPDK) by liquid chromatography-mass spectrometry (LC-MS) (Xevo G2-XS QTof). After 2 h incubation at RT, we detected both apo-VadK at the expected mass/charge (m/z = 50498) and phosphorylated-VadK with an increased mass shift of ~80 Da in accordance with a phosphorylation event (Fig. 2A). Phosphorylation was dependent on the presence of both Mg^2+^ and ATP (Fig. 2A). These results show that VadK, despite being a truncated relative of PPDK, retains its kinase activity.

Autophosphorylation of canonical PPDK’s occurs at a catalytic histidine in the kinase domain (Herzberg et al, 1996) (Minges et al, 2017) and we posit this is also the case for VadK. Using AlphaFold3 (Abramson et al, 2024) we predicted the structure of VadK and by overlaying this onto solved PPDK structures (Fig. EV1E) we identified a putative autophosphorylation site at His422. To test this prediction, we mutated VadK-His422 to alanine, checked that VadK^His422Ala^ was folded in a similar manner to VadK^WT^ by circular dichroism (CD) (Appendix Fig. S1), and repeated the autophosphorylation assay with VadK^WT^ and VadK^His422Ala^. For these experiments, the His-tag used for purification was not removed. We showed that the His-tag had no effect on autophosphorylation of VadK^WT^ (Appendix Fig. S2), however the mutant VadK^His422Ala^ did not autophosphorylate and remained in its apo form (m/z = 52272) (Fig. 2B), confirming that His422 is the catalytic residue of VadK. Peptide sequencing of apo- and phosphorylated-VadK by LC-tandem MS analysis confirmed the in-silico prediction that His422 was the phosphorylated residue (Appendix Fig. S3).

### VadK binding sites are essential for growth on propionate

To directly assess the function of the phosphohistidine and ATP-binding domains in propionate metabolism, we constructed an isogenic panel of *vadK* mutants by complementing ∆*vadK* with a chromosomally integrated copy of *vadK* encoding mutations in the His422 catalytic residue; non-phosphorylatable-VadK (∆*vadK:vadK*^His422Ala^) or phosphomimetic-VadK (∆*vadK:vadK*^His422Asp^) or a disrupted ATP-binding site, ∆*vadK:vadK*^Arg106Lys^ (predicted from (Ye et al, 2001) to disrupt ATP binding) and compared these strains to WT and with ∆*vadK:vadK* (Basu et al, 2018).

In standard 7H9 media ∆*vadK*, ∆*vadK:vadK*^His422Ala^, ∆*vadK:vadK*^His422Asp^ and ∆*vadK:vadK*^Arg106Lys^ *Mtb* strains grew slower than the parental and complemented strains, but after 21 days incubation, there was no significant differences between the strains (Appendix Fig. S4A). In 7H9 media with propionate ∆*vadK:vadK*^WT^ grew as WT. However, the mutant strains expressing non-phosphorylatable-∆*vadK:vadK*^His422Ala^, phosphomimetic ∆*vadK:vadK*^His422Asp^ or a non-functional ATP-binding site ∆*vadK:vadK*^Arg106Lys^ were unable to grow (Appendix Fig. S4B). These data show that kinase activity is critical for VadK’s role in propionate metabolism, as neither the phosphomimetic nor the non-phosphorylatable VadK were able to restore VadK growth on propionate.

### VadK does not transcriptionally regulate the MCC during propionate metabolism

Bacterial histidine kinases are typically part of two-component regulators that modulate gene expression. To test whether VadK regulates the transcription of genes associated with the MCC, we performed global transcriptional profiling. WT and ∆*vad*K were grown to mid-log phase in standard 7H9 media before being washed and inoculated into 7H9 media with propionate. After 4 h incubation, total RNA was isolated and sequenced. These data revealed no significant differences between the expression of genes encoding enzymes of the MCC (*prpC, prpD, icl1*) and MCC transcriptional regulator (*prpR*) or any other genes associated with CCM in ∆*vadK*, indicating that VadK is not involved in the transcriptional regulation of the MCC or CCM under the conditions tested (Dataset EV1). This work indicated that VadK is a non-canonical bacterial histidine kinase.

The RNA-seq analysis did, however, identify 28 upregulated genes and 21 downregulated genes (genes differentially expressed log2 fold change ≥±1.2) in ∆*vadK* compared to WT (*q* < 0.01). This data was significantly correlated with expression data obtained for *Mtb* growing in cholesterol (Pawełczyk et al, 2021). Of the 28 upregulated genes in ∆*vadK*, 13 of these were “cholesterol upregulated” genes. This included several transporters associated with efflux of lipids, antibiotics and metals (Appendix Fig. S5). Of the 21 downregulated genes, there were six genes that were also downregulated in cholesterol growth conditions (Appendix Fig. S5). These included *pe15* and *ppe20* that encode a complex that has been shown to transport calcium (Boradia et al, 2022).

### Methylcitrate synthase and methylcitrate dehydratase are interacting partners of VadK

Having established that VadK is not directly controlling the gene expression of the MCC, we then tested the hypothesis that VadK interacts directly with MCC-associated proteins. We performed co-immunoprecipitation experiments using WT *Mtb* transformed with a plasmid encoding a FLAG-tagged VadK under a constitutive promoter (VadK*Mtb*). We immunoprecipitated from whole-cell lysates of WT and VadK*Mtb* grown in standard 7H9 media with and without propionate to identify interactions that are important during growth in propionate. By applying a stringent cut-off to filter out nonspecific binding (proteins with peptide spectrum matches (PSMs) of each replicate ≤1 in the WT control and ≥50 in VadK*Mtb*), we identified interactions between VadK and the MCC enzymes, MCS and MCD (Table 1).

**Table 1:** VadK interacts with proteins involved in the methylcitrate cycle.

Rank	Gene	Name	Description	PSM VadKMtb	
				7H9	7H9 + prop
1	*rv1131*	*prpC*	2-methylcitrate synthase	7.5	56
2	*rv1130*	*prpD*	2-methylcitrate dehydratase	10.5	71.5
3	*rv1020*	*mfd*	Transcription-repair-coupling factor	38	52
4	*rv1179c*	*rv1179c*	Helicase ATP-binding domain-containing protein	46	52
5	*rv2935*	*ppsE*	Phenolphthiocerol/phthiocerol polyketide synthase subunit	78	85.5

*Mtb* transformed with a plasmid encoding VadK-FLAG under a constitutive promoter (VadK*Mtb*) were grown in standard 7H9 media (7H9) and standard 7H9 media and propionate (7H9 + prop). VadK*Mtb* was immunoprecipitated from whole-cell lysates, and interacting proteins were identified by MS. Data were calculated from two biological replicates. Nonspecific binding peptides were removed by setting the filter of Peptide spectrum matches (PSMs) of each replicate to '≤1' in the WT control samples and '≥50' in 'VadK*Mtb*' samples. Hits were ranked in descending order based on the ratio of average PSM’s from VadKMtb grown in 7H9 + propionate versus 7H9. Raw data have been deposited to the ProteomeXchange Consortium via the PRIDE (Perez-Riverol et al, 2025) partner repository with the dataset identifier PXD073941.

### Loss of VadK dysregulates central carbon metabolism, energetics and redox homoeostasis in cholesterol/propionate conditions

We previously demonstrated that ∆*vadK* is unable to grow in either cholesterol or propionate, even with the addition of an alternative carbon source (Basu et al, 2018). To test whether this phenotype is caused by defects in the MCC, we applied ^13^C-isotopomer analysis to measure changes in metabolic fluxes using different ^13^C-labelled carbon sources. As *∆vadK* cannot grow in media containing propionate/cholesterol, we grew the strains in growth-permissive 7H9 media before washing and then shifting into non-permissive media for 48 h.

**Figure 3:**
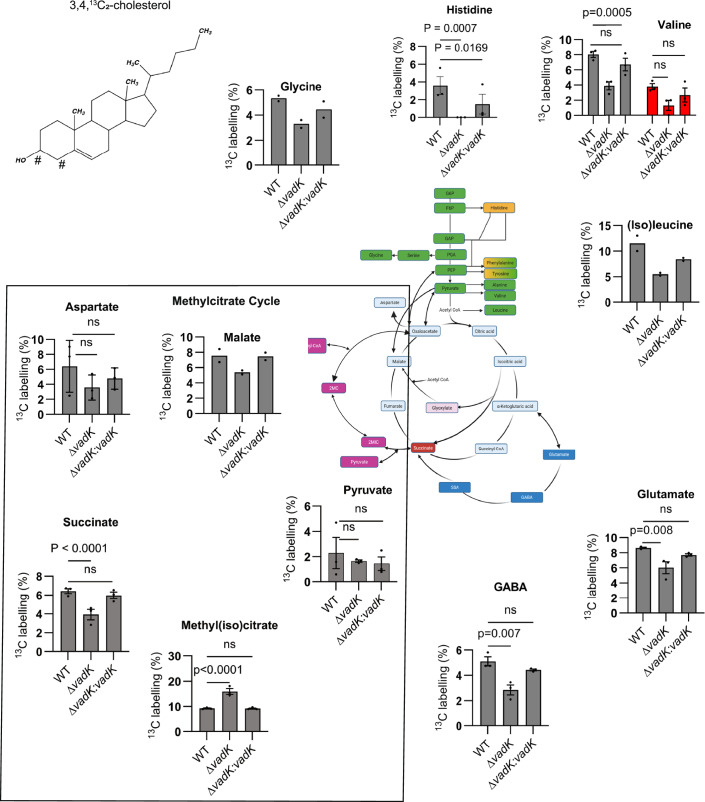
VadK-deficient *Mtb* increases flux through the methylcitrate cycle in cholesterol. ^13^C-isotopomer labelling of metabolites extracted from WT, *∆vadK* and *∆vadK:vadK* *Mtb* strains incubated in Roisin’s media containing 100% [3, 4-^13^C_2_]-labelled cholesterol for 48 h. Average percent ^13^C-incorporation into 1-carbon (grey) and >2-carbons (red) are shown superimposed on a metabolic map created in BioRender. Beste, D. (2026) https://BioRender.com/0hre2pf. # represents the position of the ^13^C label in cholesterol. Data information: Data were presented as mean ± SEM if *N* = 3 biological replicates, and statistical significance was made using an ordinary two-way ANOVA (Dunnett’s multiple Comparison test). The full dataset has been deposited in the MetaboLights (Yurekten et al, 2023) repository with the study identifier MTBLS13906.

Firstly, using [3,4-^13^C_2_] cholesterol as a tracer and sole carbon source in Roisin’s minimal media, we metabolically profiled WT, ∆*vadK* and complement after 48 h (Fig. 3). Labelling of intracellular metabolites were measured by MS as previously described (Thomson et al, 2022). Cholesterol catabolism yields four propionyl-CoAs, four acetyl-CoAs, one succinyl-CoA and a pyruvate that contains the ^13^C labelling. There was no significant difference in ^13^C-labelling of pyruvate and alanine, indicating that ∆*vadK* can generate pyruvate from cholesterol at levels comparable to WT. The incorporation of [^13^C_2_] into alanine and valine accordingly reflects the entry point of the [3,4-^13^C_2_] cholesterol into metabolism (Fig. 3). MCC was active and complete in all three strains, as evidenced by ^13^C-incorporation into 2-methyl(iso)citrate (MS cannot distinguish between 2-methylcitrate and 2-methylisocitrate) (Fig. 3).

**Figure EV3:**
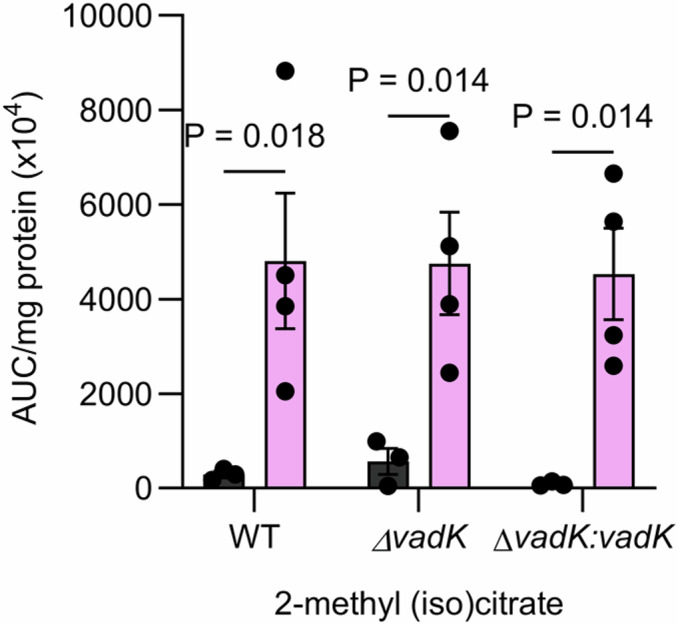
Abundance of 2-methyl(iso)citrate. MS measurements of intracellular 2-methyl(iso)citrate from *Mtb* strains grown in either Roisin’s minimal media with cholesterol (black bars) or 7H9 with 20 mM propionate (pink bars) for 48 h. Abundances are shown as normalised AUC (Methods). Data information: Mean ± SEM (*n* = 3–4 biological replicates). Statistics was calculated using an unpaired two-tailed *t*-test with Welch’s correction.

Surprisingly, there was significantly increased incorporation of ^13^C into 2-methyl(iso)citrate in ∆*vad*K compared to WT and complemented strain (Fig. 3), consistent with enhanced flux through MCS and/or MCD in ∆*vadK* that could potentially generate elevated and toxic concentrations of the MCC intermediates. However, 2-methyl(iso)citrate (Fig. EV3) and the levels of any other metabolite measured were not significantly different between the strains in these conditions (Appendix Fig. S6).

An alternative explanation is that an overactive MCC consumes oxaloacetate (OAA) and other intermediates required both to fuel the rest of CCM and to grow. In support of this hypothesis, there was reduced ^13^C-incorporation into the intermediates of the oxidative branch of the tricarboxylic acid cycle (TCA) cycle (iso)leucine, and succinate) in ∆*vadK* and reduced flux through the gamma-aminobutyric acid (GABA) shunt (<^13^C incorporation into glutamate and GABA), gluconeogenesis (<^13^C label in glycine) and no flux into the pentose phosphate pathway (PPP) (no ^13^C- labelled histidine was detected) as compared with the WT and complement (Fig. 3). Overall, these results phenocopy the metabolic profile that we previously reported for intracellular ∆*vadK* within macrophages, conditions where this strain is also unable to grow (Basu et al, 2018).

**Figure 4:**
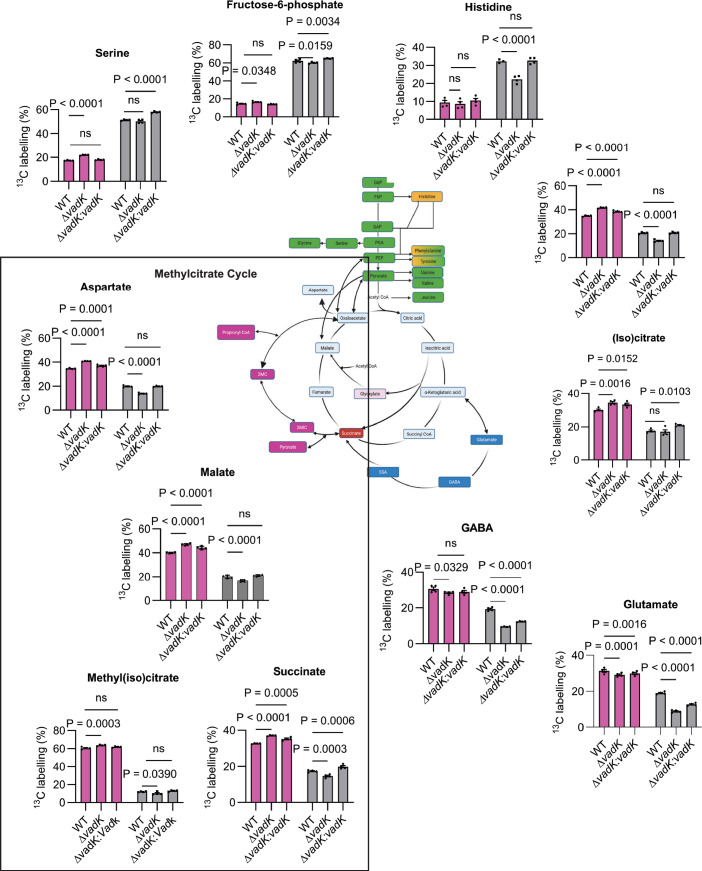
VadK-deficient *Mtb* increases flux of carbon derived from propionate through the methylcitrate cycle. WT, *∆vadK* and *∆vadK:vadK* *Mtb* strains were exposed to 7H9 media (contains unlabelled glutamate and glucose) with either [^13^C_3_]glycerol and unlabelled propionate OR [^13^C_3_]propionate and unlabelled glycerol for 24 h. The metabolites were extracted, and the percent ^13^C-labelling from propionate (pink bars) and glycerol (grey bars) was measured by MS. Metabolic map created in BioRender. Beste, D. (2026) https://BioRender.com/0hre2pf. Data information: Data were presented as mean ± SEM if *N* = 3 biological replicates, and statistical significance was made using an ordinary two-way ANOVA (Dunnett’s multiple comparison test). The full dataset has been deposited to MetaboLights (Yurekten et al, 2023) repository with the study identifier MTBLS13906.

To directly explore the role of VadK in the metabolism of propionate, we performed a mirrored ^13^C labelling experiment. We profiled WT, ∆*vad*K and complement after 48 h of labelling in 7H9 media containing either fully labelled [^13^C_3_]glycerol and unlabelled propionate or [^13^C_3_]propionate and unlabelled glycerol. This approach allowed us to calculate how much of the carbon in each metabolite was derived from propionate and how much was derived from glycerol. These experiments demonstrated there were significant differences between the flux of carbon from propionate and glycerol in ∆*vadK* as compared with WT and the complemented strains (Fig. 4).

All three strains had an active and complete MCC with more than 60% of the carbon in 2-methy(iso)citrate derived from propionate (Fig. 4). This shows that even in WT *Mtb* flux through MCS/MCD is higher than through the rest of the MCC where 32–47% of the carbon backbone from succinate, malate and aspartate (as a proxy for OAA) were derived from propionate (Fig. 4). Consistent with enhanced flux through the MCC, loss of VadK led to a significantly increased flux of propionate into all intermediates of this pathway as compared with WT (Fig. 4).

The different media and ^13^C labels used limit direct quantitative comparison between ^13^C-experiments (Figs. 3 and 4); however, there are notable differences worth discussion. Under cholesterol-only conditions, the elevated flux into 2-methyl(iso)citrate measured for Δ*vadK* (Fig. 3) was greater than that detected for ^13^C-propionate (Fig. 4) but did not extend to other MCC intermediates, likely due to differences in flux through the glyoxylate shunt. This pattern differs from that measured for propionate, where we measured significantly increased ^13^C-incorporation into 2-methyl(iso)citrate, and MCC intermediates malate, aspartate (as a proxy for OAA), and succinate (Fig. 4). Together, these results demonstrated that loss of *vadK* results in increased flux through the MCC when *Mtb* is grown in either cholesterol or propionate.

Again, we did not detect differences in 2-methyl(iso)citrate levels in Δ*vadK* compared with WT and complement, suggesting that 2-methyl(iso)citrate toxicity doesn’t explain the growth defect of this mutant in propionate conditions (Fig. EV3). There was notably ~10-fold more 2-methyl(iso)citrate detected in propionate versus cholesterol conditions in all *Mtb* strains tested that could not be explained by differences in amounts of carbon provided (Fig. EV3). However, we did detect a small but significant reduction in the relative levels of aspartate and alanine in Δ*vadK* compared with the parental strain (Appendix Fig. S7). As these amino acids are directly derived from OAA and pyruvate, respectively, key intermediates of the MCC, these data provide further evidence of increased MCC flux in Δ*vadK* in propionate conditions, limiting their production. The mutant also showed a small but significant relative reduction in pool size of glutamate, GABA, and serine as compared with the parental strain (Appendix Fig. S7).

Labelling from ^13^C-glycerol was significantly reduced in ∆*vadK* in comparison to WT in all metabolites detected except serine and (iso)citrate, where no significant change was measured (Fig. 4). This suggested that increased flux of propionate through the MCC in ∆*vadK* was affecting glycerol metabolism more generally.

**Figure 5:**
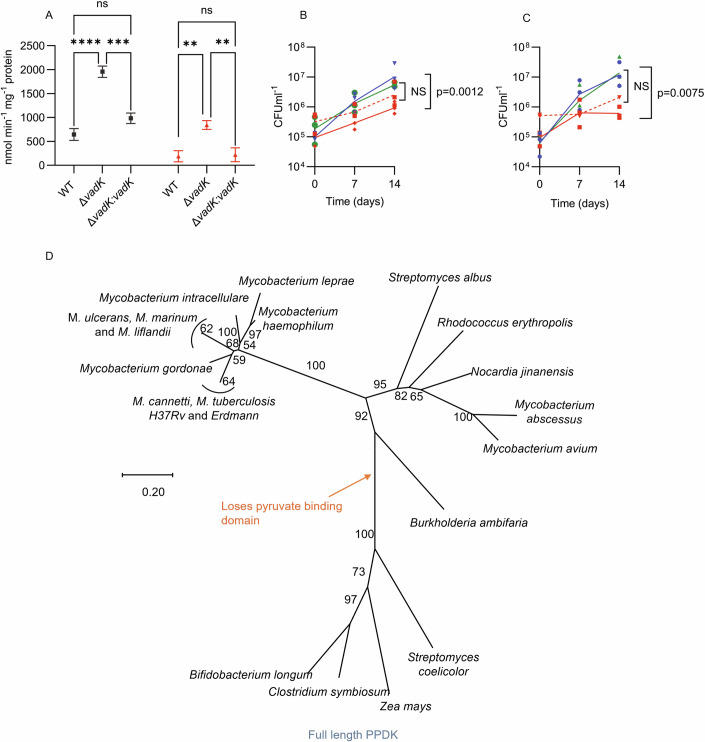
VadK-deficient *Mtb* displays increased MCS activity, attenuated growth on pyruvate and glycerol, and evidence of VadK in related organisms. (**A**) WT, *∆vadK*, *∆vadK:vadK* were grown to mid-log in 7H9 media, then shifted into 7H9 media containing 10 mM propionate for 1 or 4 days before MCS activity was measured. WT (blue) and *∆vadK* (red), *∆vadK:vadK* (green). *Mtb* strains were grown in Roisin’s minimal media containing either (**B**) glycerol or (**C**) pyruvate as the sole carbon source. *∆vadK* is rescued by the addition of vitamin B_12_ (red dotted line). Growth was measured by enumerating the CFU’s. (**D**) The evolutionary history was inferred with MEGAX (Kumar et al, 2018) using (Whelan and Goldman, 2001). The percentage of trees in which the associated taxa clustered together is shown next to the branches. Branch lengths indicate the number of substitutions per site. All positions containing gaps and missing data were eliminated. Data information: In (**A**–**C**), data were presented as mean ± SEM if *N* = 3 biological replicates and statistical significance was made using an ordinary two-way ANOVA (Dunnett’s multiple Comparison test). Source data are available online for this figure.

Kinetic modelling predicts that MCS controls the catabolic flux through the MCC (Tummler et al, 2018). To validate the increase in flux through the MCC, given that MCS was identified as an interacting partner of VadK, we measured MCS activity in cell-free extracts of WT, ∆*vadK* and ∆*vadK:vadK*. We performed carbon shift experiments as described (Muñoz-Elías et al, 2006) in which *Mtb* strains were grown to mid-log phase in standard 7H9 media, then shifted into 7H9 media plus propionate and incubated for 1 or 4 days before MCS activities were measured (Fig. 5A). These data showed that in accordance with the flux data ∆*vadK* had significantly increased MCS activity after 1 (303%) and 4 days (447%) as compared with the WT and complemented strain. These data confirm the increased flux through the MCC and provide further evidence that VadK is regulating the MCC.

**Figure EV4:**
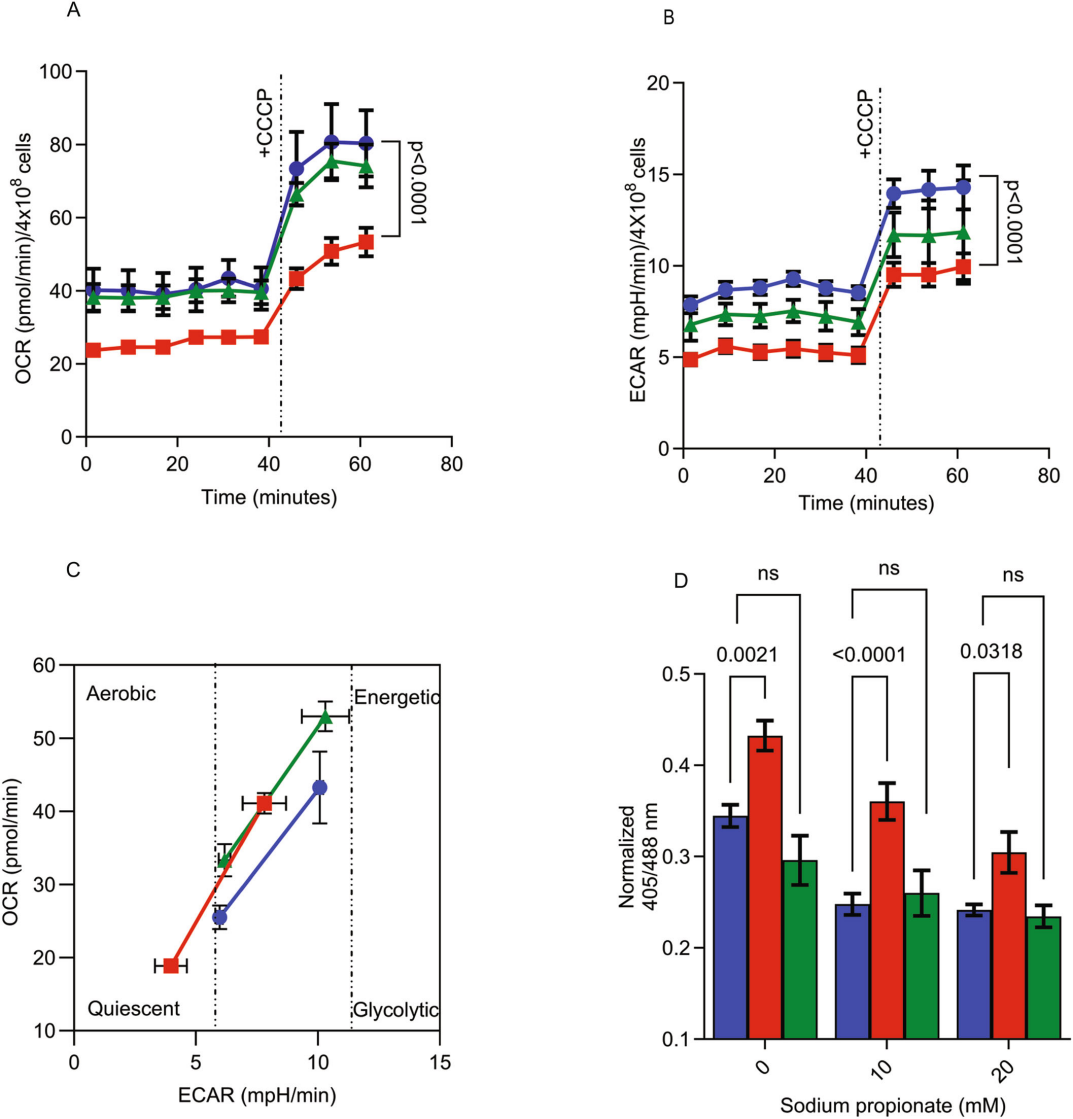
VadK-deficient *Mtb* has reduced oxygen consumption rate (OCR), extracellular acidification rate (ECAR) and is more oxidised. The metabolic potential of WT, *∆vadK* and *∆vadK:vadk* was assessed using the Agilent Seahorse XF cell energy phenotype test. Basal and stressed energy profiles were generated by measuring (**A**) oxygen consumption rate (OCR) and (**B**) extracellular acidification rate (ECAR), before and after treatment with the mitochondrial uncoupler CCCP. (**C**) The cell energy phenotype profile indicates that *∆vadK* has low energetics in comparison to WT and complement. OCR and ECAR are normalised to colony-forming unit (CFU). (**D**) The mycothiol redox potential (EMSH) of WT (blue) and *∆vadK* (red), *∆vadK:vadK* (green) in 7H9 media with glycerol and propionate as indicated was determined by measuring Mrx1-roGFP2 biosensor ratio (405/488 nm) using flow cytometry. Data information: Mean ± SEM (*n* = 9 biological replicates). Statistical significance was calculated using a one-way ANOVA with Dunnett’s multiple comparison test.

To complement the ^13^C-labelling experiments, Seahorse extracellular flux assays were performed to measure cellular bioenergetics of WT, ∆*vadK* and ∆*vadK:vadK* in 7H9 media with propionate. The bioenergetic phenogram indicated that ∆*vadK* has an energetically quiescent phenotype in concordance with reduced TCA flux and no growth in these conditions (Fig. EV4A–C).

Given that reduced flux through both the oxidative branch of the TCA cycle and the PPP in ∆*vadK*, we hypothesised that cellular redox homoeostasis could be perturbed in the mutant. To test this hypothesis, we determined the mycothiol redox potential (EMSH) using a previously described redox biosensor (Mrx1-roGFP2) that measures the concentrations of reduced mycothiol [MSH] and oxidised mycothiol disulfide [MSSM] (Bhaskar et al, 2014). An increase in the 405/488 ratio indicates an oxidative shift in the MSH/MSSM ratio. As shown (Fig. EV4D) the intramycobacterial redox state of ∆v*adK* was significantly more oxidised than either the WT or complemented strain of *Mtb* in propionate conditions which is concordant with ^13^C data showing the reduction of flux through the PPP and oxidative arm of the TCA cycle (Figs. 3 and 4).

### ATP accumulates in ∆*vadK*

Given that ∆*vadK* perturbs CCM and that VadK requires ATP for activity, we measured ATP levels in* Mtb* strains grown in 7H9 medium with or without propionate (Appendix Fig. S8). No significant differences in ATP were observed among WT, ∆*vadK*, and complemented strains in standard 7H9 medium. In propionate-containing media, ATP levels were elevated in all strains after 7 days (Appendix Fig. S8). This was not due to an artefact of the extraction process (Mulholland et al, 2025) as the cells were lysed by bead-beating (see Methods). By 14 days, WT and ∆*vadK:vadK* strains returned to levels comparable to propionate-free conditions. In contrast, ∆*vadK* maintained higher ATP levels than the WT and complement.

### Loss of VadK does not affect cell wall lipid biosynthesis

As propionyl-CoA can also be channelled directly into the biosynthesis of cell wall lipids, we extracted lipids from WT, ∆*vadK* and ∆*vadK:vadK* grown in 7H9 media with propionate and analysed them by two-dimensional thin layer chromatography (2D-TLC) to test the effects of VadK deletion on cell wall lipids (Appendix Fig. S9A). There were no significant differences in PDIM incorporation between WT, ∆*vadK* and ∆*vadK:vadK*, which is in accordance with previous studies that showed that incorporation of carbon from propionate into PDIM’s is a constitutive process, occurring independently of the amount of flux through the MCC (Lee et al, 2013). This result also confirmed that PDIM was maintained in our strains, driven by the frequent inclusion of propionate in our culture media, as recently described (Mulholland et al, 2024). Production of sulfolipid SL-1 was, however, slightly elevated in the mutant strain as compared to the WT and complement (Appendix Fig. S9B).

### VadK-deficient *Mtb* is attenuated for growth on glycerol and pyruvate

The role of the canonical MCC pathway is well established for the catabolism of propionate and cholesterol in Mtb and other bacteria and fungi (Dolan et al, 2018; Huang et al, 2023). However, Serafini et al (2019) demonstrated that* Mtb* can operate a reversed MCC when growing on pyruvate or lactate. We similarly showed that this was also the case for glycerol-grown *Mtb* (Borah et al, 2021). Here, ^13^C-labelling also demonstrated that ∆*vad*K had dysregulated glycerol metabolism (Fig. 4). To test the hypothesis that VadK is important for growth on carbon sources that require a reversed MCC we cultured WT, ∆*vadK* and ∆*vad*K:*vad*K in Roisin’s minimal media containing either glycerol Fig. 5B) or pyruvate as sole carbon sources (Fig. 5C). These data showed that ∆*vad*K was significantly attenuated for growth on either of these carbon sources as compared with the WT and complement (Fig. 5B,C). To confirm that this phenotype is a consequence of a dysregulated MCC, we tested whether B_12_ chemically complemented the growth phenotype. Vitamin B_12_ downregulates the expression of *prpR*-*prpD*-*prpC* in Mtb, effectively turning the MCC off (Campos-Pardos et al, 2024) and activates the methylmalonyl pathway because B_12_ is an essential co-factor for the second enzyme in this pathway, methylmalonyl-CoA mutase (MCM). In accordance with our hypothesis, vitamin B_12_ rescued the growth phenotype of ∆*vadK* in these carbon sources (Fig. 5B,C).

### Proposed mechanistic model of VadK regulation of MCC

**Figure EV5:**
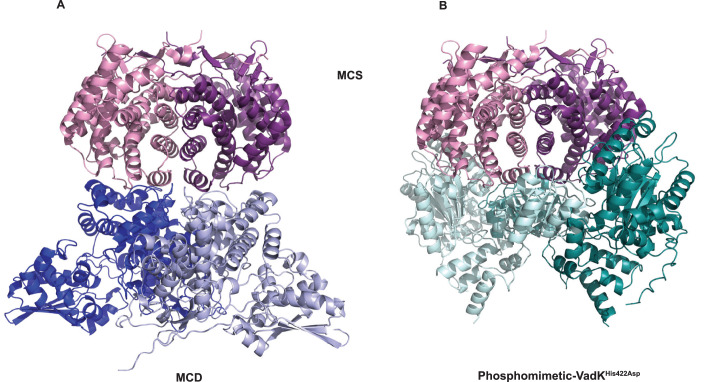
Structure prediction of MCS/MCD and MCS and VadK. (**A**) Cartoon representation of the AlphaFold3 (Abramson et al, 2024) predicted heterotetrameric complex of MCS (pink/purple) and MCD (blue/light blue) with medium confidence. (**B**) Cartoon representation of the predicted heterotetrameric complex of MCS (pink/purple) and phosphomimetic VadK^His422Asp^ (cyan/light cyan) with medium confidence. Data information: Models were generated using AlphaFold3 (Abramson et al, 2024) and visualised in PyMOL. Prediction statistics for (**A**) MCS/MCD ipTM/pTM scores is 0.41/0.55 and (**B**) MCS/VadK ipTM/pTM scores is 0.36/0.49.

Collectively, our data indicate that VadK regulates the methylcitrate cycle (MCC) by modulating the activity of MCS and/or MCD. Our pull-down assays indicated that this is modulated through direct interactions between VadK and the MCC enzymes. In addition to our AlphaFold3 structure of VadK (Fig. EV1E), we also predicted the structures of MCS and MCD and found that AlphaFold3 supports the formation of a heterotetrameric MCS-MCD complex (Fig. EV5A). Furthermore, AlphaFold3 predicts that MCS can also form a heterotetrameric complex with VadK and, notably, that phosphomimetic VadK^His422Asp^ binds MCS with higher predicted affinity than unphosphorylated VadK (Fig. EV5B).

Co-transcription of the genes encoding these MCC enzymes, *prpC* and *prpD* (Fig. EV1C), further supports the possibility that MCS and MCD form a functional complex (Griffin et al, 2011). Biologically, such an interaction could be advantageous for Mtb, as it would facilitate channelling of the toxic methylcitrate intermediate between enzyme active sites, thereby minimising toxicity to the rest of the cell.

Based on these observations, we propose a working model in which VadK competes with MCD for binding to MCS, disrupting the putative MCS-MCD complex. Furthermore, binding of phosphorylated-VadK to MCS modulates MCS activity. Future work will focus on validating these predictions through experimental determination of the protein complexes.

### PPDK has evolved into VadK in all pathogenic mycobacteria and related genera

Having established the significant role of VadK in the metabolism and virulence of *Mtb*, we wanted to explore the distribution of VadK across mycobacteria and related genera. This analysis (Fig. 5D) showed that, in addition to Mtb, VadK is present in many other pathogenic mycobacteria, including the causative agent of leprosy (*Mycobacterium leprae*), bovine TB (*Mycobacterium bovis*), Buruli Ulcer (*Mycobacterium ulcerans*) and within *Mycobacterium abscessus* - an important pathogen in people with chronic lung disease. However, non-pathogens such as *Mycobacterium smegmatis* completely lack VadK, whilst other mycobacteria have full-length PPDK but lack critical motifs for kinase activity (*Mycobacterium paraseoulense* and *Mycobacterium kiuyosense*). Additionally, VadK was identified in the genome of other actinomyces, including Nocardia, Streptomyces and Rhodococcus spp. indicating that VadK may have evolved from PPDK multiple times or has been acquired by horizontal gene transfer.

## Discussion

Phosphorylation is a widely used mechanism to regulate protein function during adaptation to different physiological conditions in the host. In bacteria, this is typically mediated via protein kinases that phosphorylate serine, tyrosine or threonine, or histidine/aspartate-based two-component regulators. We have discovered a unique, non-canonical histidine (di)kinase, VadK, that has evolved from an ancestral PPDK in pathogenic mycobacteria, including *Mtb* and other related actinomyces. We have demonstrated that VadK is a functional kinase that carries out a regulatory role in the MCC that is essential for *Mtb* growth and virulence in animal models of TB.

Metabolism of propionate via the MCC is a metabolic strategy that is critical for the virulence and survival of several pathogens in the host (Dolan et al, 2018). Propionate can be derived from the metabolism of host-derived carbon sources such as sterols, branched-chain amino acids and fatty acids and is also present at high (mM) levels during certain diseases such as the lungs of cystic fibrosis patients (Ghorbani et al, 2015). However, flux through the MCC presents microbes with a challenge. MCC intermediates can inhibit microbial growth, and the pathway diverts intermediates of the TCA cycle that must be replenished through anaplerotic pathways. Therefore, the MCC must be carefully regulated to prevent collateral harm to the rest of CCM.

Some of the first studies to explore the role of the MCC in metabolism and virulence of microbes were performed using strains of* Mtb* with deletions that interrupted the MCC (Muñoz-Elías et al, 2006). These strains were growth attenuated in media containing either propionate or cholesterol (Muñoz-Elías et al, 2006). Although the exact mechanism of growth inhibition remains unknown, propionate toxicity is correlated with reduced flux through the TCA, reduced energy production and inhibition of the PPP in *Mtb* (Dolan et al, 2018; Eoh and Rhee, 2014).

Here, we have identified that ∆*vadK* has a growth and metabolic phenotype strikingly similar to that of MCC-deficient strains but without any lesion in the MCC. *Mtb* strains lacking VadK exhibit increased flux through the MCC that prevents in vitro growth of *Mtb* in propionate/cholesterol-containing media and attenuates growth on media with glycerol or pyruvate as the sole carbon source. These phenotypes are driven by the impact of increased flux through the MCC that competes for TCA intermediates, reducing flux through the oxidative branch of the TCA cycle and through the PPP, causing changes in redox balance.

RNA-seq analysis demonstrated that VadK is not transcriptionally regulating the MCC and showed a significant overlap with the transcriptomic response to cholesterol, specifically the upregulation of detoxification systems and efflux of lipids/antibiotics/mycobactin and the downregulation of genes involved in amino acid and lipid metabolism as well as calcium import. These transcriptional signatures correlate to increased ATP under these conditions, as calcium and ATP levels are intrinsically linked. Calcium transport is dependent on ATP, but its import also enhances ATP production.

Interestingly, this work demonstrated that levels of 2-methyl(iso)citrate are significantly higher when *Mtb* is exposed directly to propionate versus substrates that yield propionyl-CoA, such as cholesterol. The regulatory mechanisms underpinning these differences require further investigation. This is significant as it has been shown that* Mtb* has a mixed diet in the host, which includes propionyl- CoA, yielding carbon sources such as sterols, whereas *M. abscessus* within a cystic fibrosis lung has direct access to propionyl-CoA (Beste et al, 2013; Dolan et al, 2018).

We previously demonstrated that VadK is required for the growth of *Mtb* in macrophages (Basu et al, 2018), and here we extended this work to show that it is also required for virulence in two different murine models of TB. The connection between the MCC and virulence has been observed previously. For example, blocking the MCC impairs the virulence of several pathogenic fungi and bacteria (Dolan et al, 2018). The MCC is necessary for the virulence of the rice blast fungus, *Pyricularia oryzae* (Yan et al, 2019) and *Aspergillus fumigatus* (Ibrahim-Granet et al, 2008) and is important to *Neisseria meningitidis* infection and transmission (Catenazzi et al, 2014). By contrast, whilst deleting methylcitrate lyase (MCL) activity (encoded by *icl* genes) or *prpDC* from *Mtb* impaired growth in macrophages, only *icl* mutants are attenuated in vivo (Muñoz-Elías and McKinney, 2005). Interpretation of this result is complicated in *Mtb* as MCL’s can also function as isocitrate lyase’s in the glyoxylate shunt, so deleting the MCL ablates both pathways. Elegant studies by Rhee et al (2014) indicated that in vitro the defects associated with *icl* deficiency are due to a broken MCC. Breaking the MCC at MCL leads to the build-up of the toxic intermediate 2-methylisocitrate (Eoh and Rhee, 2014). It is presumed that in vivo, *prpDC* is dispensable because there is sufficient B_12_ to activate the B_12_-dependent methylmalonyl pathway to metabolise propionate. Vitamin B_12_ can also downregulate the MCC by repressing the co-transcription of *prpDC* and the MCC transcriptional regulator *prpR* (Campos-Pardos et al, 2024). In contrast to disrupting the MCC, we show that dysregulating flux through the MCC severely attenuates *Mtb* in vivo and so represents a better strategy to target* Mtb*.

The requirement of VadK for virulence and survival in the murine models suggests that in vivo B_12_ concentrations do not fully repress the MCC and/or that VadK regulates MCC, but via a mechanism other than transcription. Although murine serum B_12_ levels vary significantly, they are generally in the picogram range rather than the microgram levels of B_12_ required to rescue ∆*vadK* (Campos-Pardos et al, 2024). In vivo data indicate that while B_12_ concentrations are adequate to induce the methylmalonyl pathway, they are not high enough to completely suppress the MCC (Campos-Pardos et al, 2024). As an extension of our work, it would be interesting to test the phenotype of ∆*vadK* in a B_12_ anaemic murine model of TB. Future studies will be required to establish the reach of VadK’s regulatory effects.

It was previously established that a reverse MCC pathway is utilised by *Mtb* grown on pyruvate, lactate (Serafini et al, 2019) and glycerol (Borah et al, 2021). However, *prpDC* is not essential for growth in either pyruvate or lactate, demonstrating that although the MCC is active under these conditions, it is not essential (Serafini et al, 2019). By contrast, we have demonstrated that deletion of VadK attenuates the growth of *Mtb* in either glycerol or pyruvate and that this phenotype is rescued by supplementation with B_12_, demonstrating that downregulation of the MCC and/or activation of the methylmalonyl pathway restores growth of this mutant.

Overall, this work demonstrates that dysregulation of the MCC caused by the loss of VadK has broader impacts on *Mtb* metabolism and growth than complete loss of the pathway, and links MCC hyperactivity to a slow- or non-replicating phenotype. These findings raise the intriguing hypothesis that fine-tuning flux through the MCC could serve as another mechanism by which Mtb regulates its growth rate. Given that VadK’s autophosphorylation requires ATP, it could function as a sensor of cellular energy state: high ATP levels drive VadK-mediated suppression of MCC flux, while low ATP could enhance MCC flux, imposing a brake/slowdown of growth. Supporting this hypothesis, we demonstrated that ATP levels increase in response to propionate even in WT Mtb, but this is reduced over time. In the absence of VadK, however, ATP levels remained elevated. We and others have previously demonstrated that ATP is growth inhibitory to Mycobacteria, and this therefore could be contributing to the growth attenuation of ∆*vadK* (Lodhiya et al, 2024; Tatano et al, 2015). Further work is required to test this hypothesis.

Our phylogenetic trees indicate that VadK orthologues are conserved across pathogenic mycobacteria—including amongst *M. leprae’s* minimal set of proteins, supporting a key role for VadK in mycobacterial pathogenesis and its potential as a drug target. As propionate accumulates in the lungs during certain diseases, such as cystic fibrosis patients at millimolar concentrations (Ghorbani et al, 2015), careful regulation of flux through the MCC is likely to be important to various pathogens that cause disease in this environment. Notably, non-tuberculous mycobacteria, including *M. abscessus* that causes the most severe disease and highest mortality in individuals with chronic lung conditions such as chronic obstructive pulmonary disease, cystic fibrosis, and bronchiectasis, encodes a VadK orthologue. We also identified VadK in *Burkholderia ambifaria*, a plant-associated bacterium that causes opportunistic infections in humans, particularly in cystic fibrosis patients (Vinacour et al, 2023), suggesting VadK may contribute more broadly to bacterial virulence, particularly in hosts with chronic lung disease.

In summary, we have identified a novel kinase, VadK, that is essential for *Mtb* growth on carbon sources metabolised through the MCC and causes TB. VadK likely functions to fine-tune flux through the methylcitrate cycle, so coordinating MCC activity with the rest of the CCM and the cell's energy state. The presence of VadK orthologues in other mycobacterial and non-mycobacterial pathogens suggests that this kinase plays a role in the pathogenesis of organisms that rely on the MCC within the host. Importantly, VadK has no homologues in humans, highlighting its potential as a candidate for future drug development.

## Methods

**Table:** Reagents and tools table

Reagent/resource	Reference or source	Identifier or Catalogue Number
**Experimental models**		
*Mycobacterium tuberculosis* H37Rv	ATCC	ATCC 27294
*M. tuberculosis* H37RvΔvadK (nee Δppdk)	Basu et al, 2018	
*M. tuberculosis H37RvΔvadK:vadK (nee Δppdk:ppdk)*	Basu et al, 2018	
*M. tuberculosis H37RvΔvadK:* Mrx1-roGFP2	This study	
*M. tuberculosis H37RvΔvadK:vadK: Mrx1-roGFP2*	This study	
*M. tuberculosis H37RvΔvadK:* vadK ^His422Asp^	This study	
*M. tuberculosis H37RvΔvadK:* vadK ^His422Ala^	This study	
*M. tuberculosis H37RvΔvadK: vadK* ^Arg106lys^	This study	
*M. tuberculosis H37Rv*: *pKP1153*	This study	
*Balb/c mice* (female, 6 weeks old)	Vaarunya Biolabs Private Limited	
*C3HFeB/FeJ mice* (female and male, 6 weeks old)	National Center for Biological Science	
**Recombinant DNA**		
pKP1153	This study	
pMV306ppdk	Basu et al, 2018	
vadK:vadK ^His422Asp^	Genscript generated	
vadK:vadK ^His422Ala^	Genscript generated	
vadK:vadK ^Arg106lys^	Genscript generated	
Mrx1-roGFP2	Bhaskar et al, 2014	
**Antibodies**		
Anti-FLAG M2 affinity matrix	Sigma	Cat # A2220
**Oligonucleotides and other sequence-based reagents**		
VadK HisTag pGEX6p1 Precission	Genscript	SC1691
VadK H422A HisTag pGEX6p1 Precission	Genscript	SC1626
VadK Forward NdeI	Invitrogen	75643297
Universal Reverse XhoI	Invitrogen	75643297
VadK H422D Forward	Azenta/Genewiz	30-1074820235
VadK H422D Reverse	Azenta/Genewiz	30-1074820235
Rv1126c HisTag pET28a-TEV	Genscript	SC1691
QRT-PCR primers	Forward	Reverse
16 s rRNA	F: 5’ GCCGTA AACGGTGGGTACTA 3'	R: 5’ TGCATGTCAAACCCAGGTAA 3'
*rv1127c (vadK)*	F:5’GCGGTATTCGCCTCCTGGAACTCACCTC3’	R:5’AGCCATTCGCCGAACGGTTCGTTGGCTC3’
**Chemicals, Enzymes and other reagents**		
Lysis matrix B	MPBio	Cat # 691150
Dodecyl-B-D maltoside	Avanti	Cat # 250520 P
DNase	New England Biolabs	Cat # M0303S
[U-13C3]-glycerol	CKisotopes	Cat # CLM-1510
[U-13C3]-sodium pyruvate	CKisotopes	Cat # CLM-2440
[U-13C3]-sodium propionate	CKisotopes	Cat # CLM 1865 MPT-PK
[U-13C2]-cholesterol	CKisotopes	Cat # CLM-804
ATP kit	Thermo Fisher	Cat # A22066
RNA clean-up kit	New England Biolabs	Cat # T2030
Cumene hydroperoxide	Sigma-Aldrich	Cat # 247502
Dithiothreitol	Sigma-Aldrich	Cat # D9779
Cell-Tak–coated XF 439	Corning	Cat # 354240
Pfu Ultra Polymerase	Agilent	600385
KOD Polymerase	Sigma-Aldrich	71086-3
T7 Express lysY	NEB	C3010I
DH5alpha	Thermo Fisher	18258012
Trypsin Gold	Promega	V5280
Iodoacetamide	Thermo Fisher	AC122271000
DTT	Thermo Fisher	R0861
Ammonium bicarbonate	Fisher Scientific	AC393212500
ATP	Sigma-Aldrich	A2383-5G
Magnesium Chloride	Fisher Scientific	AC413415000
Tris	MP Biomedical	152176
Sodium Chloride	Fisher Scientific	S271-10
Glycerol	Fisher Scientific	G37-4
Imidazole	Thermo Scientific	122020020
FastRNA™ Pro Blue Kit	MP Biomedicals™	116025050
RNeasy Kit	QIAgen	74136
Ribopure DNA-free Kit	Invitrogen	AM1928
iScript cDNA Synthesis Kit	Bio-Rad	170-8891
iQ SYBR Green Supermix	Bio-Rad	1708886
**Software**		
Xcalibur 3.0	Thermo Scientific	
Proteome Discoverer v1.4	Thermo Scientific	
MassHunter Qualitative v10	Agilent	
GraphPad Prism 10.6.1	Graphpad Software, LLC	
MassLynx	Waters	4.1 SCN909
Bio Pharma Lynx	Waters	1.3.5
**Other**		
Spin-X columns	CoStar	Cat # 8161
Fast Prep	Hybaid	
Orbitrap Fusion Lumos MS	Thermo Scientific	
Seahorse XFp FluxPak	Agilent	103022-100
HisTrap FF crude 5 mL	Cytvia	17528601
HisPur NiNTA Resin	Thermo Fisher	88222
Superdex Increase 200 10/300	Cytvia	28990944
QIAquick Extraction Kit	Qiagen	28704
Amicon Centrifugal Filter	Sigma Millipore	UFC903008
StepOnePlus Real- Time PCR System	Applied Biosystems	

### Bacterial strains

Frozen stocks of Mtb (H37Rv) and mutant strains *∆vadK* and ∆*vadK:vadK* are previously described (Basu et al, 2018). For co-immunoprecipitation, the overexpression vector pKP1153 was constructed by cloning C-terminally FLAG-tagged Rv1127c under the control of the constitutive mycobacterial Ptb38 promoter into pDE43-MEH using a Gateway cloning approach (Ehrt et al, 2005; Kim et al, 2011). Site-directed mutagenesis of pMV306ppdk (Basu et al, 2018) by Genescript generated three different alleles of *vadK: vadK*^His422Ala^, *vadK*^His422Asp^ and *vadK*^Arg106Lys^. All plasmids were transformed by electroporation into WT or *∆vadK* and selected on 7H11 plates supplemented with 25 μg ml^−1^ kanamycin or 50 μg ml^−1^ hygromycin, as appropriate. (Basu et al, 2018). PCR was used to confirm the presence of the correct gene.

### Growth conditions

*Mtb* strains were cultivated using Middlebrook 7H11 agar containing 5% (v/v) oleic acid/albumin/dextrose/catalase (OADC) enrichment medium supplement (BD) and 0.5% (v/v) glycerol. Liquid cultures were grown in standard Middlebrook 7H9 broth containing 0.2% (v/v) glycerol, 0.2% (v/v) Tween80 or Tyloxapol, and 5% (v/v) ADC or Rosins minimal media (Beste et al, 2005). For the methylcitrate synthase assay, strains were grown in 7H9 media containing 0.2% (v/v) glycerol, 0.2% (v/v) Tyloxapol, and 5% (v/v) ADC and 10- or 20-mm sodium propionate. Vitamin B12 (10 μg ml^−1^) was added when indicated.

For the sole carbon source experiments, Roisin’s minimal media (Beste et al, 2005) containing either glycerol (0.5%), pyruvate (0.165%) or cholesterol (0.154%), and 0.2% tyloxapol. Cultures were grown until mid-log phase (OD_600nm_ = 0.6–0.8) in standard 7H9, washed once with PBS, and then resuspended to a starting OD_600nm_ of 0.01. Cell growth was monitored daily by OD and/or CFU measurements. When selection was required, kanamycin at 20 μg ml^−1^ and hygromycin at 50 μg ml^−1^ were added to the culture medium.

### Animal ethics and biosafety approvals

All animal experiments adhered to the strict guidelines of the Committee for the Purpose of Control and Supervision of Experiments on Animals (CPCSEA), Government of India. The study protocols received formal approval from the Institutional Biosafety Level-3 (BSL-3) Committee as well as the Institutional Animal Ethics Committee, with specific approvals designated as IAEC: CAF/Ethics/780/2020 for the BALB/c studies and IAEC: CAF/Ethics/972/2023 for the C3HeB/FeJ experiments.

### Housing and husbandry conditions

Animal Housing and Environment: Animals were housed in Individually Ventilated Cages to maintain a pathogen-free micro-environment. The facility was maintained under a controlled 12:12 h light/dark cycle. The ambient temperature was regulated at 22 ± 2 °C with a relative humidity of 30–70%.

### Husbandry and maintenance

To ensure high standards of hygiene and animal welfare, sterile coir bedding was utilised and replaced bi-weekly to minimise ammonia buildup and moisture. All efforts were made to minimise animal suffering. Animals were monitored daily for clinical signs of distress, including changes in coat condition, posture, and activity levels. At the conclusion of the study, euthanasia was performed using Isoflurane in accordance with approved veterinary practices.

### Mouse infection models

For Infection studies, 6–8-week-old both male and female C3HeB/FeJ mice and female BALB/c mice (*n* = 7–8 per group) were infected by aerosol using the Glas-Col Inhalation Exposure System to deliver ~50 bacilli per mouse with WT, knockout and complemented* Mtb* strains as indicated (Mtb H37Rv, *ΔvadK* and *ΔvadK:vadK*). To minimise experimental bias, animals were assigned to study groups using a weight randomisation approach. This ensured inter-group homogeneity regarding initial body weight and general health status. Baseline physical parameters were assessed prior to the study to confirm that no statistically significant differences existed between groups at the start of the experiment.

Mice were housed inside the BSL-3 facility and euthanised at day 28 and day 56 post-infection. Lungs and spleens were aseptically harvested and homogenised in sterile PBS for bacillary load analysis, tissue histopathology, and pathological scoring, as described (Anand et al, 2021). Serial dilutions of tissue homogenates were plated on Middlebrook 7H11 agar plates supplemented with OADC enrichment and PANTA antibiotic mixture. Plates were incubated at 37 °C, and CFUs were enumerated after 4 weeks of incubation.

### Histopathological analysis

Lung tissues were fixed in 10% formaldehyde, embedded in paraffin, and sectioned for histological evaluation. To ensure objectivity, histopathology samples were anonymised using a numeric coding system. The pathologist remained blinded to the treatment groups throughout the processing and scoring phases to prevent investigator bias. Sections were stained with HE for pathological examination. Disease severity was assessed based on granuloma scoring, and representative histopathological images were captured for each infection group.

### RNA extraction and quantitative real-time PCR (qRT-PCR) analysis

Bacterial strains were grown till mid-log phase (OD ≈ 0.7–0.8). GTC buffer (5 M guanidium thiocyanate, 0.5% sarcosyl, 0.5% Tween80, 1% β-mercaptoethanol (freshly added)) was added to the bacterial culture, mixed, and the cells were immediately harvested. Total RNA was extracted using MP Biomedicals™ FastRNA™ Pro Blue Kit and QIAgen RNeasy Kit. DNA contamination was removed from the isolated RNA using a Ribopure DNA-free Kit and 600 ng of RNA was used for cDNA synthesis using the iScript cDNA Synthesis Kit. Quantitative RT-PCR is done with StepOnePlus Real- Time PCR System (Applied Biosystems) using gene-specific primers and iQ SYBR Green Supermix (Bio-Rad). Expression of genes were normalised with the Ct value for 16s rRNA (internal housekeeping control).

### In vitro VadK expression and purification

To improve the solubility of recombinant VadK, we expressed a slightly truncated version of the protein lacking 17 N-terminal residues that are predicted to be disordered according to AlphaFold3 (Abramson et al, 2024) (Fig S1D&E). We also found that co-expressing VadK with its operonic partner Rv1126c improved its solubility. The synthetic Mtb Rv1127c gene sequence, codon-optimised for *Escherichia coli*, was cloned into the pET28a-TEV vector (GenScript) to produce an N-terminal TEV-cleavable 6x His-tagged VadK. The neighbouring gene, Rv1126c gene sequence was codon-optimised for *E. coli* and cloned into pET22b (Novagen) to produce Rv1126c without a HisTag. Successful cloning was verified by DNA sequencing (GeneWiz from Azenta Life Sciences). Similarly, VadK^His422Ala^ was constructed in the pET28a-thrombin vector. Site-directed mutagenesis was performed using Pfu Ultra DNA Polymerase (Agilent) and Rv1127c H422A-forward (5’-TGGTGCGGCCTCCGCAGCAGCGGTGGTGTCACGTGAACTA) and Rv1127c H422A-reverse (sequence: 5’-TAGTTCACGTGACACCACCGCTGCTGCGGAGGCCGCACCA) primers (Azenta). The VadK^His422Ala^ mutant was confirmed by DNA sequencing (GeneWiz from Azenta Life Sciences).

pET28a-TEV-VadK-HisTag and pET22b-Rv1126c-noTag, were co-transformed into *E. coli* BL21-Gold (DE3) T7 Express cells (New England Biolabs Inc) and were grown in LB media containing 100 μg ml^−1^ ampicillin and 50 µg ml^−1^ kanamycin, and protein expression was induced by 1 mM IPTG. Cultures were lysed by sonication on ice (45% amplitude, 15 s on and 45 s off for 15 cycles) in Buffer A (50 mM Tris, pH 7.4, 350 mM NaCl, 10 mM imidazole), 100 µM PMSF and 0.5 mg ml^−1^ lysozyme. Proteins were loaded onto a HisTrap FF column (5 mL, GE Healthcare) using an AKTA Start FPLC and washed extensively with Buffer A containing 2 M NaCl to separate Rv1126c from Rv1127c. Bound protein was eluted from the column using an imidazole gradient (10 mM to 0.5 mM) in Buffer A. Rv1127c was pooled, concentrated, and further purified using a Superdex 200 Increase 10/300 GL column (Cytiva) equilibrated with Buffer B (50 mM Tris pH 7.4, 150 mM NaCl buffer, 10% glycerol) to yield pure fractions of VadK-HisTag (Appendix Fig. S2B). The expression and purification method for the Rv1127c^His422Ala^ mutant was carried out in an identical fashion.

TEV at a ratio of 10:1 VadK:TEV was used to remove the His-tag from VadK in Buffer B with 2 mM DDT at 4 °C. Cleaved VadK was recovered using NiNTA agarose beads (ThermoFisherScientific) and further purified using a Superdex 200 Increase 10/300 GL column (Cytiva) equilibrated with Buffer B. Pure fractions were pooled and concentrated.

### VadK autophosphorylation assays

Reaction mixtures of 25 μM VadK, 2 mM ATP and 2 mM MgCl_2_ in Buffer B were incubated for 2 h at room temperature. Samples were analysed by MS using LC-MS/MS (ACQUITY UPLC H-class system, Xevo G2-XS QTof, Waters). Phosphorylation was observed by reverse-phase chromatography at 45 °C using a C4 column (Protein BEH C4 Column, 300 Å, 1.7 μm, 2.1 mm × 50 mm, Waters) and a 5-min gradient of Buffer C (0.1% formic acid in water), Buffer D (100% acetonitrile from 0% Buffer D to 90% Buffer D with a flow rate of 0.3 mL min^−^1). The Xevo Z-spray source was run with a capillary voltage of 300 V, and a cone voltage of 40 V (NaCsI calibration, Leu-enkephalin lock-mass). N_2_ was used as the desolvation gas at 350 °C and a flow rate of 800 L/h and data acquisition was done at alternating collision energy with low energy at 0 V and high collision energy ramp from 15 to 45 V. Data were acquired in continuum with 0.5 second scans across a mass range of 400–4000 Da. Data were deconvoluted using the proprietary Water MaxEnt1 algorithm.

### Circular dichroism (CD) of Mtb VadK and its mutant

Far UV CD spectra were collected at 25 °C using a Jasco J-810 spectropolarimeter with a Digital Integration Time of 1 second and a bandwidth of 1 nm, using 0.1 cm cuvettes with VadK and its mutant (5 µM) in 5 mM Tris, pH 7.4, 35 mM NaCl, 1% glycerol. Spectra were read from 260 to 190 nm at 100 nm min^−1^ for a total of ten accumulations. The eStSel tool (https://bestsel.elte.hu/index.php) was used to quantify secondary structural elements.

### VadK sequencing by tandem MS to confirm the catalytic histidine

Reaction mixture (25 μM VadK, 2 mM ATP and 2 mM MgCl_2_, in Buffer B) was incubated for 2 h at room temperature. Samples of apo and phosphorylated-VadK (4 µM) and DTT (20 mM) in 50 mM ammonium bicarbonate (ABC) buffer, pH 7.4, were boiled at 80 °C for 20 min. After cooling, iodoacetamide (15 mM) was added to the samples and incubated in the dark at room temperature for 1 h, followed by the addition of Trypsin Gold (400 nM, Promega) and an overnight digestion at 37 °C. Samples were analysed using liquid chromatography tandem MS (LC-MS/MS, ACQUITY UPLC H-class system, Xevo G2-XS QTof, Waters) by reverse-phase chromatography at 45 °C using a C4 column (Protein BEH C4 Column, 300 Å, 1.7 μm, 2.1 mm × 50 mm, Waters) and a 5 min gradient of Buffer C and Buffer D with a flow rate of 0.3 mL min^−1^. The Xevo Z-spray source was run with a capillary voltage of 300 V, and a cone voltage of 40 V (NaCsI calibration, Leu-enkephalin lock-mass). N_2_ was used as the desolvation gas at 350 °C and a flow rate of 800 L h^−1^, and data acquisition was carried out at alternating collision energy with low energy at 0 V and high collision energy ramp from 15–45 V. Data were acquired in continuum with 0.5 s scans across a mass range of 400–4000 Da. Waters proprietary software BioPharma Lynx was used to identify phosphorylated peptide fragments, intensities, and control b/y ions. Finally, the b/y ions were then further identified in raw Xevo MS/MS data.

### Co-Immunoprecipitation of FLAG-tagged VadK

We followed the methodology described here (de Miranda et al, 2023). Briefly, 50 ml cultures of Mtb were grown in 7H9 media with 0.2% glycerol and Tyloxapol with and without 10 mM sodium propionate until late log phase (OD_600_ = 0.8–1). Cell pellets were resuspended in lysis buffer (50 mM Tris, pH 7.4, 350 mM NaCl and 10% glycerol). Complete protease inhibitor (Roche) by bead-beating with lysis matrix B using a Hybaid Fast Prep at speed (4.0) for 20 s with careful chilling between each round. Lysates were incubated with 1% *n*-Dodecyl-B-D-maltoside (Avanti) for 4 h at 4 ^o^C before centrifugation and then filtered twice through 0.22-μm Spin-X column filters (CoStar). Anti-Flag M2 affinity matrix (40 µl) (Sigma) that had been pre-washed in TBS (Tris-Buffered Saline, 50 mM Tris-HCl, pH 7.4, 150 mM NaCl) was added to the lysate and incubated overnight, rotating at 4 ^o^C. The resin was carefully washed 5 times with TBS before being resuspended in NuPAGE LDS loading dye and heated at 95 ^o^C for 5 min. Proteins were electrophoretically separated on a 10% Novex Bis-Tris SDS -PAGE gel (Invitrogen) with MES buffer, and the entire gel slice was sent for MS analysis.

Each gel slice was subjected to in-gel tryptic digestion using a DigestPro automated digestion unit (Intavis Ltd.). The resulting peptides were fractionated using an Ultimate 3000 nano-LC system in line with an Orbitrap Fusion Lumos mass spectrometer (Thermo Scientific). In brief, peptides in 1% (vol/vol) formic acid were injected onto an Acclaim PepMap C18 nano-trap column (Thermo Scientific). After washing with 0.5% (vol/vol) acetonitrile 0.1% (vol/vol) formic acid peptides were resolved on a 250 mm × 75 μm Acclaim PepMap C18 reverse-phase analytical column (Thermo Scientific) over a 150 min organic gradient, using seven gradient segments (1–6% solvent B over 1 min, 6–15% B over 58 min, 15–32% B over 58 min, 32–40% B over 5 min, 40–90%B over 1 min, held at 90%B for 6 min and then reduced to 1%B over 1 min.) with a flow rate of 300 nl min^−1^. Solvent A (0.1% formic acid) and Solvent B (80% acetonitrile in 0.1% formic acid). Peptides were ionised by nano-electrospray ionisation at 2.2 kV using a stainless-steel emitter with an internal diameter of 30 μm (Thermo Scientific) and a capillary temperature of 300 °C.

All spectra were acquired using an Orbitrap Fusion Lumos mass spectrometer controlled by Xcalibur 3.0 software (Thermo Scientific) and operated in data-dependent acquisition mode. FTMS1 spectra were collected at a resolution of 120 000 over a scan range (m/z) of 350–1550, with an automatic gain control (AGC) target of 4E5 and a max injection time of 50 ms. Precursors were filtered according to charge state (to include charge states 2–7), with monoisotopic peak determination set to peptide and using an intensity threshold of 1E3. Previously interrogated precursors were excluded using a dynamic window (40 s ±  10 ppm). The MS2 precursors were isolated with a quadrupole isolation window of 0.7 m/z. ITMS2 spectra were collected with an AGC target of 2E4, max injection time of 35 ms and HCD collision energy of 30%. The raw data files were processed using Proteome Discoverer software v1.4 (Thermo Scientific) and searched against the UniProt *Mycobacterium tuberculosis* (strain ATCC 25618 H37Rv) [83332] database (downloaded July 2022; 3993 sequences) and the VadK sequence using the SEQUEST HT algorithm. Peptide precursor mass tolerance was set at 10 ppm, and MS/MS tolerance was set at 0.6 Da. Search criteria included oxidation of methionine (+15.995 Da) and phosphorylation of histidine (+79.966 Da) as variable modifications and carbamidomethylation of cysteine (+57.021 Da) as a fixed modification. Searches were performed with full tryptic digestion, and a maximum of two missed cleavages were allowed. The reverse database search option was enabled, and all data was filtered to satisfy a false discovery rate (FDR) of 1%.

### RNA-sequencing

*Mtb* strains were grown in standard 7H9 until mid-log phase (OD_600_ = 0.6–0.8) before being spiked with 20 mM sodium propionate. After 4 h, transcription was stopped by the addition of four volumes of GTC buffer (5 M guanidine isothiocyanate, 0.5% sodium L-lauryl sarcosine, 25 mM tri-sodium citrate, pH 7 and 0.1 M 2-mercaptoethanol). RNA was extracted as described (Beste et al, 2007). RNA was treated with DNAse twice and purified (RNAeasy kit (NEB)). RNA sequencing and analysis was performed by Genewiz (Azenta). RNA samples were quantified using Qubit 4.0 Fluorometer (Life Technologies, Carlsbad, CA, USA) and RNA integrity was checked with RNA Kit on TapeStation (Agilent Technologies, Palo Alto, CA, USA). ERCC RNA Spike-In Mix (Cat: #4456740) from Thermo Fisher Scientific, was added to normalised total RNA prior to library preparation following the manufacturer’s protocol. RNA depletion was performed using the NEBNext rRNA Depletion Kit (Bacteria). RNA sequencing library preparation used NEBNext Ultra RNA Library Prep Kit for Illumina, following the manufacturer’s recommendations (NEB, Ipswich, MA, USA). Briefly, enriched RNAs were fragmented according to the manufacturer’s instructions. First-strand and second-strand cDNA were subsequently synthesised. cDNA fragments were end- repaired and adenylated at 3’ends, and a universal adaptor was ligated to cDNA fragments, followed by index addition and library enrichment with limited-cycle PCR. Sequencing libraries were validated using NGS Kit on the Agilent 5300 Fragment Analyzer (Agilent Technologies, Palo Alto, CA, USA), and quantified by using Qubit 4.0 Fluorometer (Invitrogen, Carlsbad, CA).

The sequencing libraries were multiplexed and loaded on the flowcell on the Illumina NovaSeq X Plus instrument according to the manufacturer’s instructions. The samples were sequenced using a 2 × 150 Pair-End (PE) configuration. Image analysis and base calling were conducted by the NovaSeq Control Software on the NovaSeq instrument. Raw sequence data (.bcl files) generated from Illumina NovaSeq was converted into fastq files and de-multiplexed using the Illumina bcl2fastq programme. One mismatch was allowed for index sequence identification.

Sequence reads were trimmed to remove possible adaptor sequences and nucleotides with poor quality using Trimmomatic v.0.36. The trimmed reads were then mapped to the Mtb H37Rv reference genome using the Bowtie2 aligner v.2.2.6 to generate BAM files. Unique gene hit counts were calculated by using featureCounts from the Subread package v.1.5.2. The hit counts were summarised and reported using the $gene_feature feature in the annotation file. Only unique reads that fell within gene regions were counted.

After the extraction of gene hit counts, the gene hit counts table was used for downstream differential expression analysis. Using DESeq2, a comparison of gene expression was performed. The Wald test was used to generate *p* values, *p* adjusted *q* values and log2 fold changes. Genes with <0.01 *q* values and absolute log2 fold change >1.2 were called as differentially expressed genes for each comparison.

### ^13^C-isotopologue analysis

Mtb strains were grown in standard 7H9 media to late log phase (OD_600_ = 0.8–1) before being washed and resuspended in ^13^C-labelled media and incubated for 48 h. Using [U-13C3]-glycerol (99%), 20 mM [U-13C3]-pyruvate (99%) or 0.154% [3, 4-13C2] cholesterol for 48 h. Metabolites were quenched and extracted using a slight modification of the protocol described (Thomson et al, 2022). The bacteria were metabolically quenched by plunging into acetonitrile/methanol/H2O (2:2:1), precooled to −80 °C. Metabolites were extracted by bead-beating three times in the FastPrep at 6.5 for 20 s with careful cooling in between. Soluble extracts were filtered twice through 0.22 μm Spin-X column filters (CoStar) and then stored at −80 °C until analysis.

The data were acquired with an Agilent 1290 Infinity II UHPLC coupled to a 6545 LC/Q-TOF system. Chromatographic separation was performed with an Agilent InfinityLab Poroshell 120 HILIC-Z (2.1 × 100 mm, 2.7 μm (p/n 675775-924)) column. The HILIC-Z methodology was optimised for polar acidic metabolites. Column compartment was set at 50 °C. For easy and consistent mobile-phase preparation, a concentrated 10 × solution consisting of 100 mM ammonium acetate (pH 9.0) in water was prepared to produce mobile phases A and B. Mobile phase A consisted of 10 mM ammonium acetate in water (pH 9) with a 5 μM Agilent InfinityLab deactivator additive (p/n 5191-4506), and mobile phase B consisted of 10 mM ammonium acetate (pH 9) in 10:90 (v:v) water/acetonitrile with a 5 μM Agilent InfinityLab deactivator additive (p/n 5191-4506). The following gradient was applied at a flow rate of 0.25 ml/min: 0 min, 96% B; 2 min, 96% B; 5.5 min, 88% B; 8.5 min, 88% B; 9 min, 86% B; 14 min, 86% B; 17 min, 82% B; 23 min, 65% B; 24 min, 65% B; 24.5 min, 96% B; 26 min, 96% B and 3-min of re-equilibration at 96% B. Accurate MS was performed using an Agilent Accurate Mass 6545 QTOF apparatus. Dynamic mass axis calibration was achieved by continuous infusion after the chromatography of a reference mass solution using an isocratic pump connected to an electrospray ionisation source operated in negative-ion mode. The following parameters were used: gas temperature, 225 °C; drying gas, 13 l min^−1^; sheath gas temperature, 350 °C; nebuliser pressure, 35 psi; sheath gas flow, 12 l min^−1^; capillary voltage, 3,500 V; nozzle voltage, 0 V; fragmentor voltage, 125 V; skimmer 45 V and octupole 1 RF voltage, 750 V. The data were collected in centroid 4 GHz (extended dynamic range) mode.

### LC-MS metabolomics data analysis

Data analysis was performed using the Agilent MassHunter Qualitative (v10), Quantitative Analysis and Profinder Software. Metabolite identification was based on mass-retention times and isotope distribution patterns. Metabolites were quantified using area under the curve (AUC) normalised to protein concentration determined using a standard Bradford assay.

### Determination of ATP levels

ATP was measured using the luciferase-based ATP determination kit (Thermo Fisher, USA; A22066) according to the manufacturer’s instructions. Strains of Mtb were grown in standard 7H9 media with or without 10 mM propionate for 14 days. Samples were harvested after 7- and 14-days incubation and extracted in lysis buffer (50 mM Tris pH7.4, 150 mM NaCl_2_ and 0.01% Tween80) by bead-beating with 0.1 mm zirconia-silica beads (Sigma) in the FastPrep at 6.5 for 3 × 45 s with careful cooling between bursts.

### Analysis of mycobacterial Lipids

Mtb strains were in standard Middlebrook 7H9 media for 10 days before spiking with 20 mM sodium propionate and a further 4-day incubation. Cell pellets were washed with PBS and autoclaved before extraction. Extraction of Mtb lipids and Thin Layer Chromatography (TLC) analysis was carried out using protocols described by Dobson et al (1985) (DOBSON et al, 1983). Dry weights of cell pellets were used as a measure to equalise loading on TLC plates using solvent Systems A (direction 1; Petroleum ether/ethyl acetate 98:2 × 3, direction 2; Petroleum ether/acetone 98:2), System B (direction 1; Petroleum ether/acetone 92:8 ×3, direction 2; Toluene/acetone 95:5), System C (direction 1; Chloroform/methanol 96:4, direction 2; Toluene/acetone 80:20), System D, (direction 1; chloroform/methanol/water 100:14:0.8, direction 2; chloroform/acetone/methanol/water 50:60:25:3).

### Measurement using Mrx1-roGFP2 redox biosensor

The Mrx1-roGFP2 ratio was determined during in vitro growth of *Mtb* strains in glycerol (0.2%) or propionate (10 mM and 20 mM) containing 7H9 medium as described in (Das et al, 2023). Briefly, bacterial cultures expressing Mrx1-roGFP2 were treated with 5 mM *N*-ethylmaleimide (Sigma-Aldrich, St. Louis, MO) for 5 min at room temperature, followed by 4% paraformaldehyde (PFA) fixation (Himedia, Mumbai, India) for 1 h at room temperature. Bacteria were analysed using a FACSVerse flow cytometer (BD Biosciences, San Jose, CA). The biosensor response was quantified by measuring the fluorescence ratio at a fixed emission (510 nm) on excitation at 405 and 488 nm. The data obtained were analysed with the BD FACSuite software. These ratiometric data were normalised to measurements of cells treated with 10 mM CHP (Sigma-Aldrich, St. Louis, MO), giving maximal oxidation of the biosensor, and 20 mM dithiothreitol (Sigma-Aldrich, St. Louis, MO), yielding a readout of maximal reduction of the biosensor. Ten thousand events per sample were analysed.

### OCR and ECAR measurements

To assess the metabolic activity of *Mtb* strains, the basal oxygen consumption rate (OCR) and extracellular acidification rate (ECAR) were measured using the Agilent Seahorse XFp Analyzer. Log-phase *Mtb* cultures were subjected to nutrient starvation in Middlebrook 7H9 broth lacking albumin, dextrose, and sodium chloride (ADS) or any additional carbon source, and supplemented with tyloxapol, a non-metabolisable detergent, to prevent bacterial clumping. Following starvation, cultures were centrifuged at 100×*g* for 5 min to enrich for single-cell suspensions. The resulting suspensions were washed thoroughly with unbuffered 7H9 medium to remove residual nutrients. A total of 4 × 10⁶ bacterial cells per well were seeded into the wells of a Cell-Tak–coated XF cell culture microplate (Corning, Cat. No. 354240). To ensure equal seeding density across wells, CFU validation was performed by plating aliquots on Middlebrook 7H11 agar and enumerating colonies after a 4-week incubation at 37 °C. OCR and ECAR were measured using unbuffered 7H9 assay medium (pH 7.45; lacking disodium phosphate and monopotassium phosphate). The medium was supplemented with 20 mM sodium propionate and 0.2% glycerol as the sole carbon sources. Measurements were recorded over a period of approximately 60 min. To assess respiratory capacity, carbonyl cyanide 3-chlorophenylhydrazone (CCCP) (Sigma-Aldrich, Cat. No. C2759) was added at a final concentration of 10 μM through designated drug injection ports during the assay, as indicated in corresponding figure legends. Changes in OCR and ECAR in response to substrate addition or CCCP stimulation were calculated and expressed as pmol/min per 4 × 10⁶ cells.

### Crude enzyme extractions

*Mtb* was grown to late log phase (OD_600_ = 0.8–1.0) in standard 7H9 broth. Bacterial pellets were harvested and resuspended in the same media with or without 10 mM propionate and incubated for 1 or 4 days. Preparation of cell-free extracts was performed basically as described by Muñoz-Elías et al (2006). Briefly, bacterial pellets were lysed in buffer (150 mM NaCl_2_, 50 mM Tris, 10% (v/v) glycerol, 1 mM phenylmethylsulfonyl fluoride (PMSF) at pH 7.4 by bead-beating as described for the ATP assay. Extracts were clarified by centrifugation and filtration through a 0.45 µm pore Spin-X filter (Corning), and the total protein concentration was determined by BCA assay. Cell-free extracts were standardised to 0.25 mg ml^−1^ frozen and stored at −80 °C.

### Methylcitrate synthase assay

The enzyme activity of crude extracts was performed as described by Muñoz-Elías et al (2006), with some modifications. Briefly, MCS activity was measured at 30 ^°^C using the reaction buffer: 50 mM Tris, pH 7.4, 150 mM NaCl_2_, 2 mM 5,5-dithio-bis-(2-nitrobenzoic acid (DTNB), 0.5 mM OAA, 0.3 mM propionyl-CoA. Activity was measured at spectrophotometrically at 400 nm.

### Phylogenetic analysis

PPDK sequences of typical mycobacteria and other relevant organisms were downloaded from UniProt (Consortium, 2024). The evolutionary history was inferred with MEGA X (Kumar et al, 2018), sequences were aligned using the MUSCLE algorithm, and a phylogeny was generated using the maximum likelihood method with the optimised Whelan and Goldman + Freq. model (Whelan and Goldman, 2001).

## Supplementary information

Appendix

Peer Review File

Dataset EV1

Source data Fig. 1

Source data Fig. 5

Expanded View Figures

## Data Availability

The mass spectrometry proteomics data have been deposited in the ProteomeXchange Consortium via the PRIDE (Perez-Riverol et al, 2025) partner repository with the dataset identifier PXD073941. The metabolomics data have been deposited in the MetaboLights (Yurekten et al, 2023) repository with the study identifier MTBLS13906. In vitro protein phosphorylation mass spectrometry raw data have been deposited in the MassIVE repository (https://massive.ucsd.edu) under accession MSV000101031 (https://massive.ucsd.edu/ProteoSAFe/dataset.jsp?task=884edcdb3a4140f0ad96f55d5de79f6a). The source data of this paper are collected in the following database record: biostudies:S-SCDT-10_1038-S44319-026-00818-0.
