# Supplementary material for: Mycobacterium tuberculosis VadK is required for the regulation of the methylcitrate cycle and virulence
Source: EMBO Rep. 2026 Jun 11;27(14):4100–23. doi: 10.1038/s44319-026-00818-0 (PMC13400623; doi:10.1038/s44319-026-00818-0)
Supplement: Supplementary file 1 — Appendix [file 44319_2026_818_MOESM1_ESM.pdf]

Appendix for *Mycobacterium tuberculosis* VadK is required for the regulation of the methylcitrate cycle and virulence

Table of Contents

Appendix Figure S1. VadK-His422Ala is correctly folded.

Appendix Figure S2. VadK-His can auto-phosphorylate.

Appendix Figure S3. MS/MS spectra of b/y ion for peptide fragment.

Appendix Figure S4. Strains of Mtb expressing isogenic forms VadK cannot grow in the presence of propionate.

Appendix Figure S5. The absence of VadK is associated with shared transcriptional profile for Mtb growing in cholesterol.

Appendix Figure S6. No detectable change in metabolite pool size upon *vadK* deletion in Mtb compared with the parent strain when grown on cholesterol.

Appendix Figure S7. Reduction in the pool size of selected metabolites upon vadK deletion in Mtb compared with the parent strain when grown on propionate.

Appendix Figure S8. ATP levels increase in response to propionate in Mtb.

Appendix Figure S9. No significant changes in cell wall lipids in Mtb lacking *vadK*, except for sulphur-lipids.

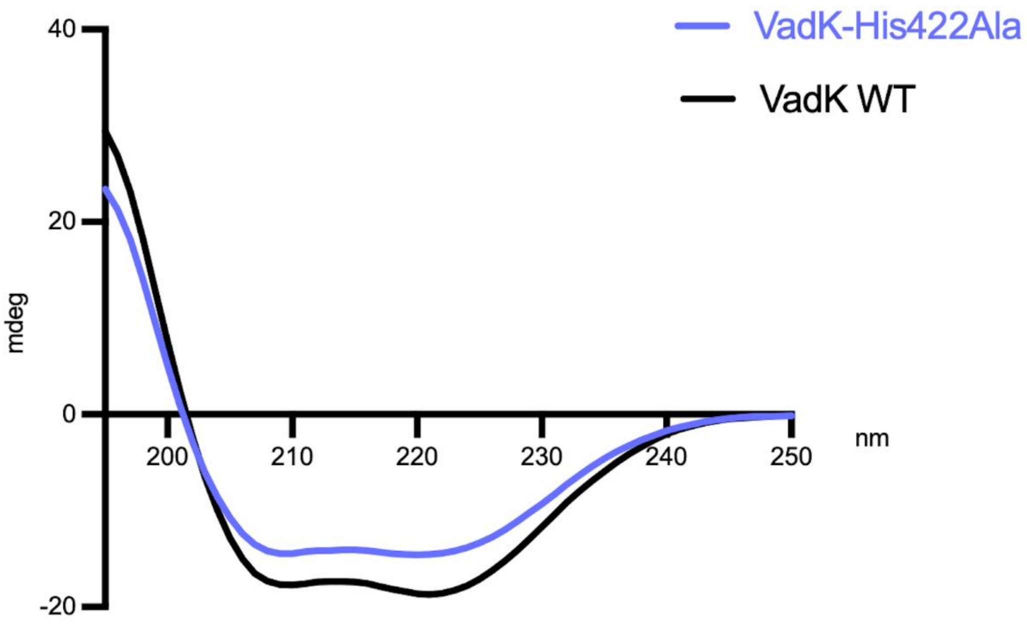

Appendix Figure S1. VadK-His422Ala is correctly folded. Circular dichroism spectra of WT VadK (black line) and VadK-His422Ala (blue line) are similar, indicating that the mutant has the same secondary structure as WT VadK and is folded correctly. This is representative results of three different protein preparations of both VadK and mutant.

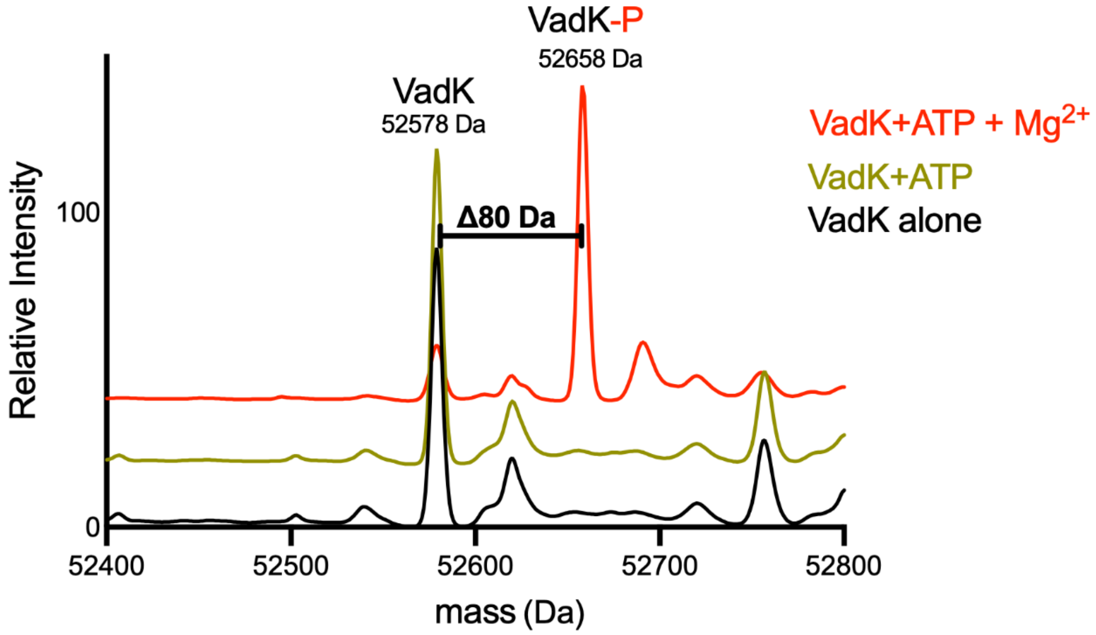

Appendix Figure S2. VadK-His can auto-phosphorylate. MS of VadK-His alone (black line), with ATP and in the absence (green line) and presence of Mg^{2+} (red line). A phosphorylation event (mass shift 80 Da, designated by VadK-P) only occurs for apo-VadK-His in the presence of ATP and Mg^{2+}. This is representative of at least triplicate experiments for each spectrum. The raw and deconvoluted data is deposited in the MassIVE repository (https://massive.ucsd.edu) under accession MSV000101031.

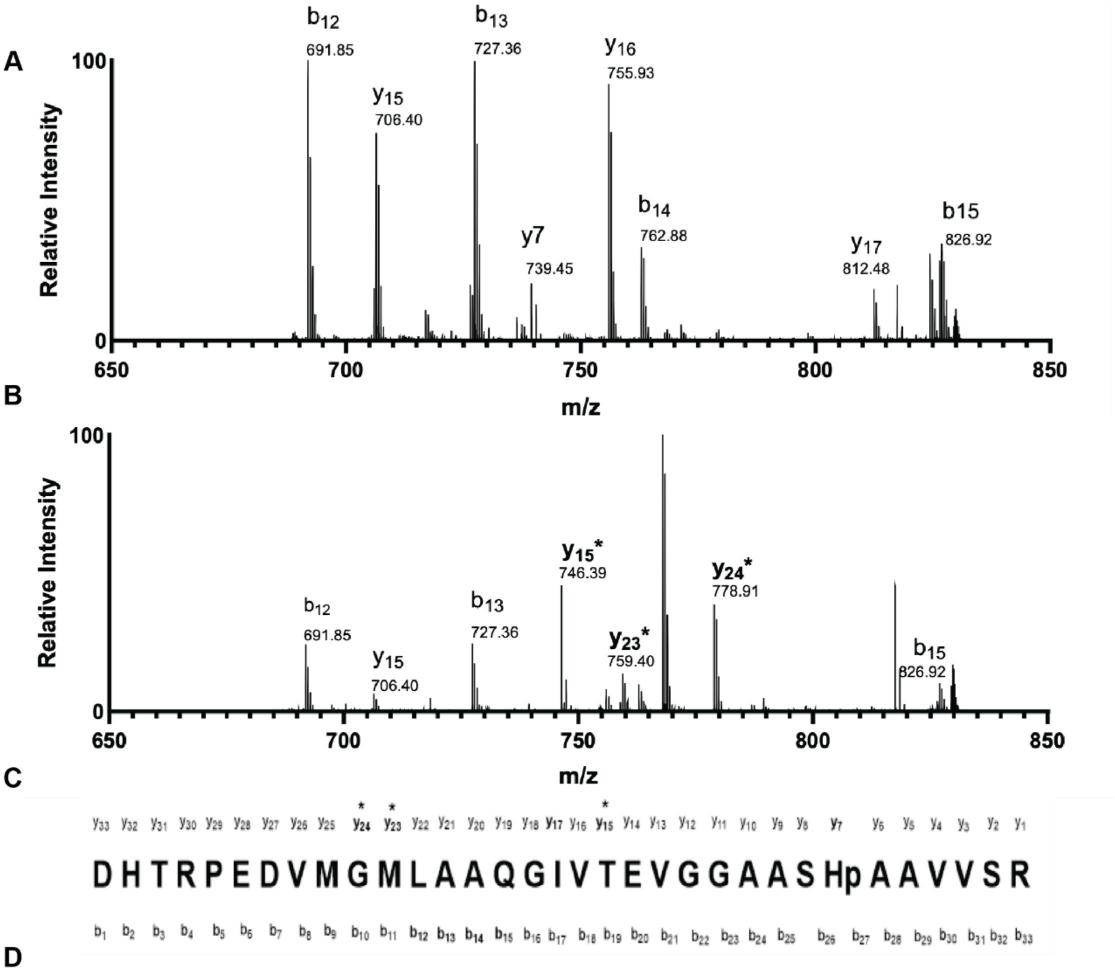

D

Ion Assignment	Sequence	Fragment Mass (Da)	Charge state Found	Predicted Mass (Da)	Observed Mass (Da)	Control Mass Error (Da)
b12	DHTRPEDVMGML	1399.62246	2nd	691.81325	691.8459	0.03265
b13	DHTRPEDVMGMLA	1470.65958	2nd	727.33181	727.3638	0.03199
b14	DHTRPEDVMGMLAA	1541.69669	2nd	762.85036	762.8831	0.03274
b15	DHTRPEDVMGMLAAQ	1669.75527	2nd	826.87965	826.9172	0.03755
y15	RSVVAAHSAAGGEVT	1410.72158	2nd	706.36809	706.3978	0.02971
y15*	RSVVAAHpSAAGGEVT	1490.72158	2nd	746.36809	746.3869	0.01881
y7	RSVVAAH	738.41371	1st	739.42099	739.4527	0.03171
y17	RSVVAAHSAAGGEVTVI	1622.87406	2nd	812.44433	812.4786	0.03427
y23*	RSVVAAHpSAAGGEVTVIGQAALM	2274.15288	3rd	759.05827	759.4047	0.34643
y24*	RSVVAAHpSAAGGEVTVIGQAALMG	2331.17434	3rd	778.06542	778.9081	

Appendix Figure S3. MS/MS spectra of b/y ion for peptide fragment. DHTRPEDVMGMLAAQGIVTEVGGAASHAAVVSR, encompassing amino acid residues 401-433. Comparison of peptide fragment pattern when VadK is (A) untreated and (B) treated with ATP and Mg^{2+}. Identification of phosphorylated y ions are labelled with an asterisk and bolded. Notably, the presence of phosphorylated y15 ion suggest that histidine phosphorylation is on H422. (C) peptide sequence denotes order of b and y ions, with p representing phosphorylation modification and identified phosphorylated ion in bold with an asterisk. (D) Table of fragmentation masses which are listed together with the charge state. b and y ions with asterisk indicate phosphorylated ion identified. Lowercase p in sequence refers to phosphorylation. This is representative of duplicate biological experiments for WT

and VadK-His422Ala. The raw and deconvoluted data is deposited in the MassIVE repository (https://massive.ucsd.edu) under accession MSV000101031.

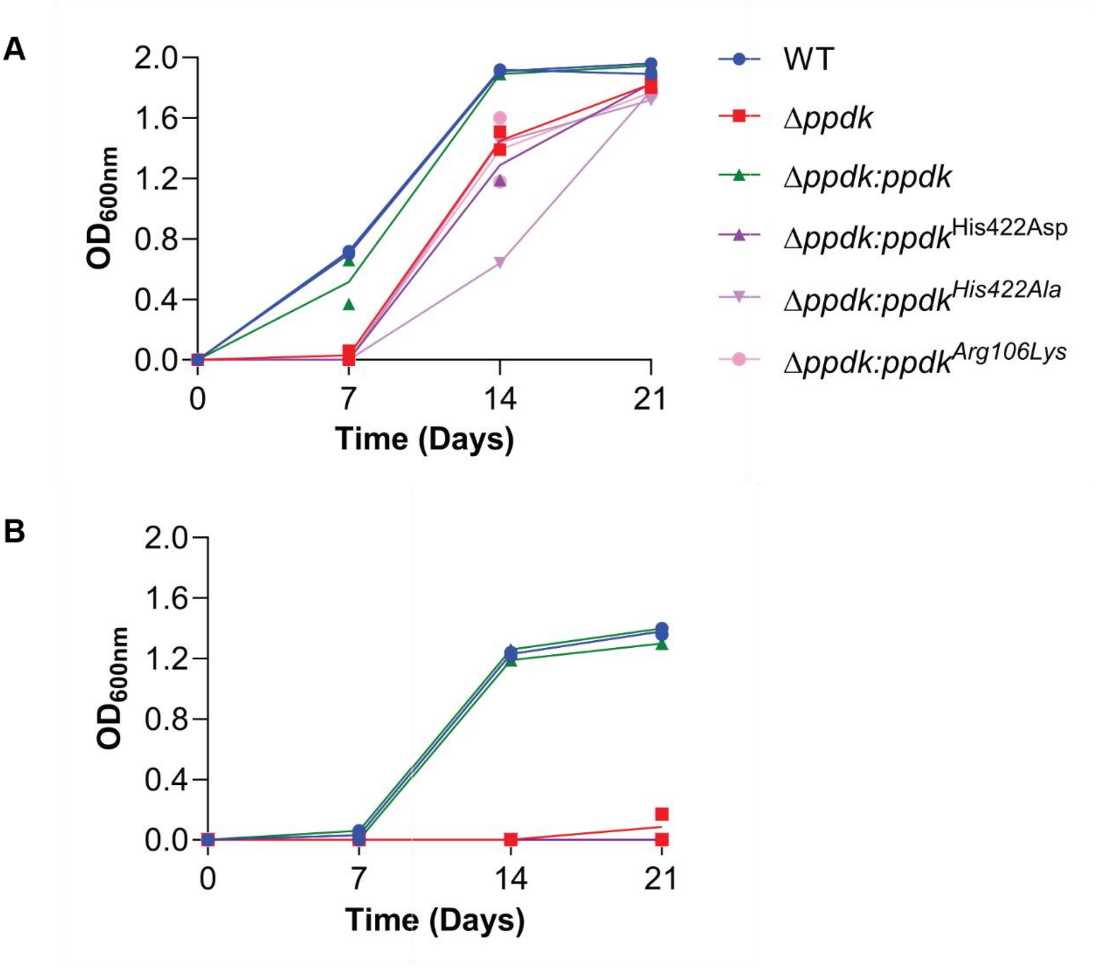

Appendix Figure S4. Strains of Mtb expressing isogenic forms VadK cannot grow in the presence of propionate. ∆*vadK*, ∆*vadK:vadK* and Mtb expressing isogenic forms of VadK that is unphosphorylatable because the catalytic histidine has been mutated to alanine (His422>Ala) or is a phosphomimetic because the histidine has been mutated to aspartate (His422>Asp) or unable to bind ATP (Arg106>Lys) were grown in 7H9 media without (A) or with 10 mM propionate (B). Growth was measured by OD (600nm). The data represent the average (n=2 biological replicates).

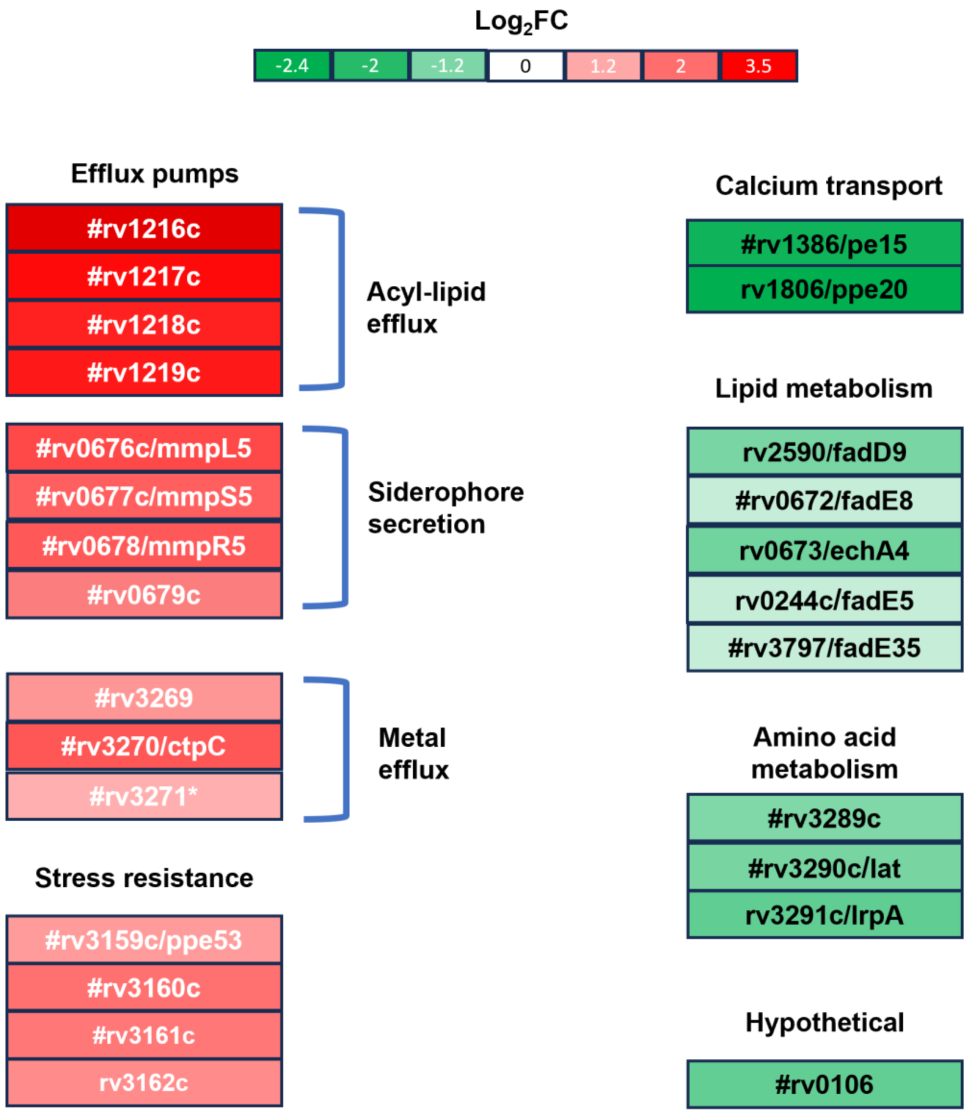

Appendix Figure S5. The absence of VadK is associated with shared transcriptional profile for Mtb growing in cholesterol. RNA-seq was used to quantify differentially expressed genes between WT and Δ*vadK* grown in 7H9 medium containing glycerol and 20 mM propionate. Data are displayed as $\log_2$ fold change in gene expression (Δ*vadK* vs parental Mtb). # identified as differentially expressed in cholesterol growth conditions (Pawełczyk et al., 2021). Data from (n = 3 biological replicates; adjusted q-value ≤ 0.001). All processed data can be found in Appendix Supplementary Dataset 1.

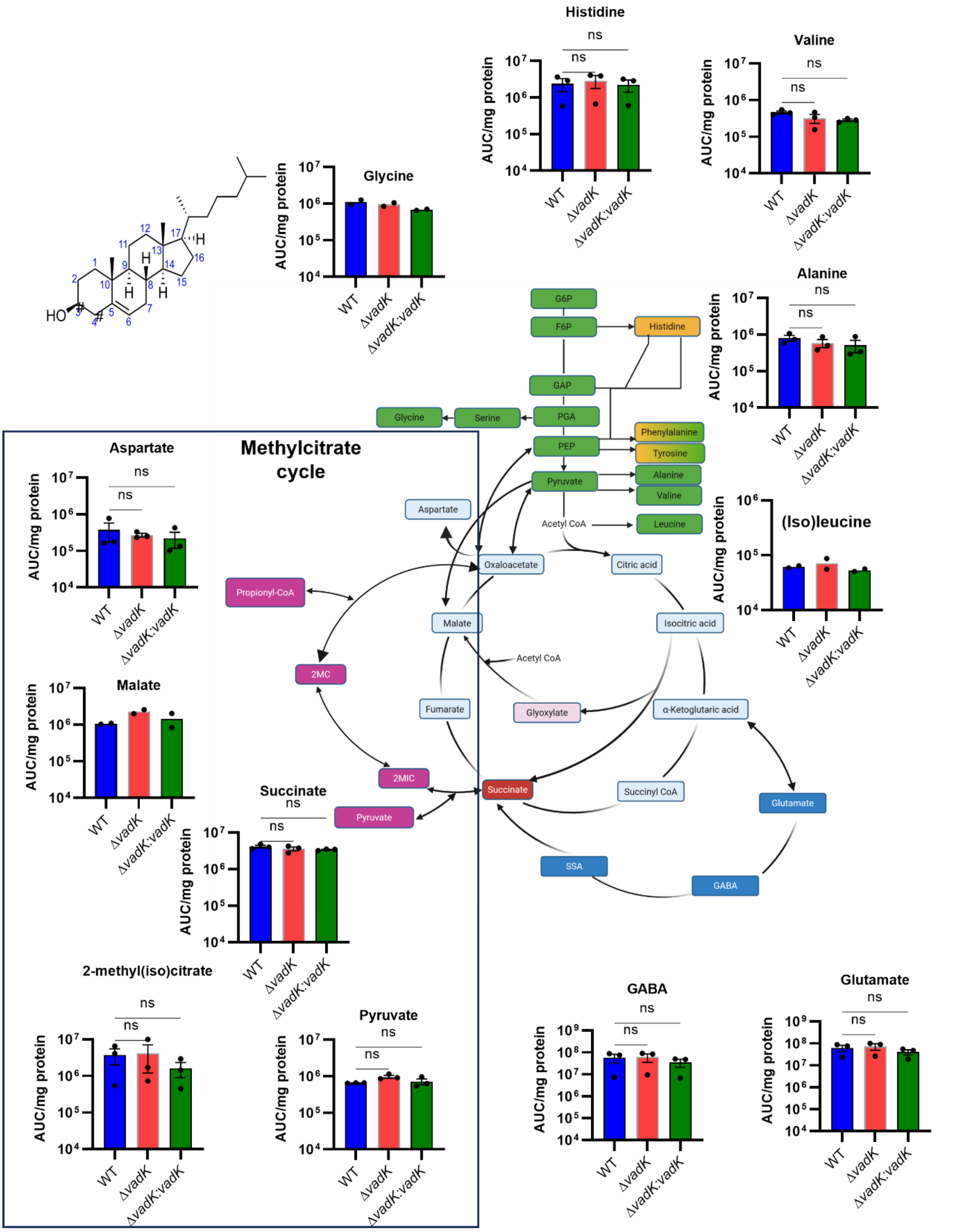

Appendix Figure S6. No detectable change in metabolite pool size upon *vadK* deletion in Mtb compared with the parent strain when grown on cholesterol. MS measurements of intracellular metabolites from of Mtb grown in Roisin's minimal media with cholesterol for 48 h. Abundances are shown as normalized AUC (Methods). Mean ± SEM (n=2-3 biological replicates). Statistics was calculated using

an unpaired two-tailed t-test with Welch's correction. Schematic of central carbon metabolism was created with biorednder.com.

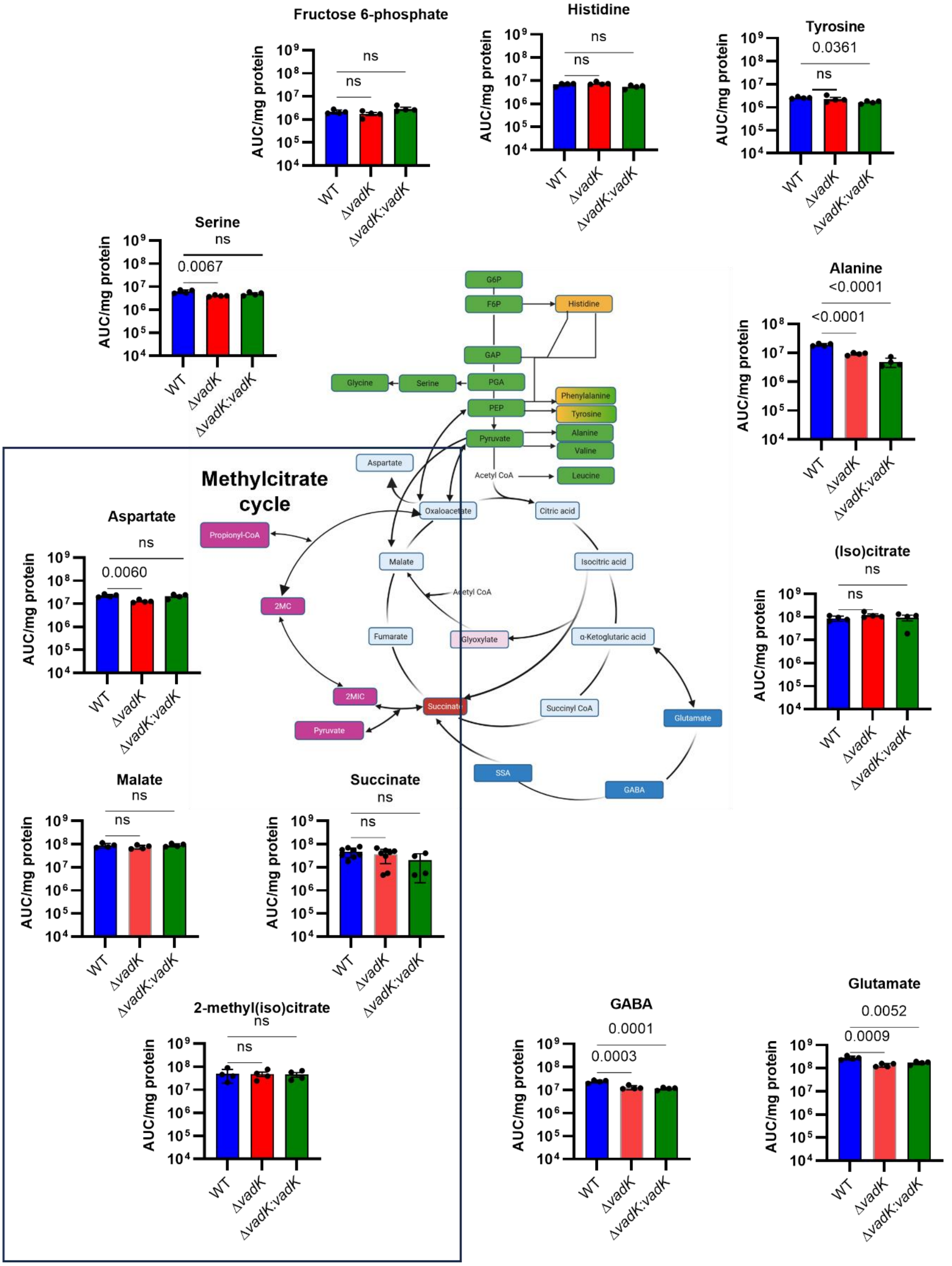

Appendix Figure S7. Reduction in the pool size of selected metabolites upon *vadK* deletion in Mtb compared with the parent strain when grown on propionate. MS measurements of intracellular metabolites from indicated strains of

Mtb grown in Roisin's minimal media with cholesterol for 48 h. Abundances are shown as normalized AUC (Methods). Mean ± SEM (n=4 biological replicates). Statistics was calculated using an unpaired two-tailed t-test with Welch's correction. Schematic of central carbon metabolism was created with biorednder.com.

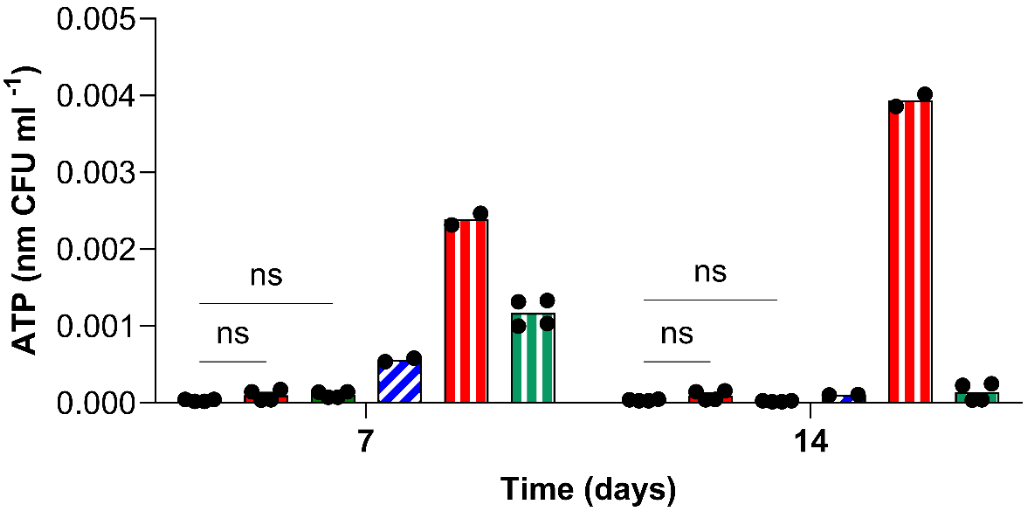

Appendix Figure S8. ATP levels increase in response to propionate in Mtb. ATP levels Appendix in WT (blue bars), ∆*vadK* (red bars) and ∆*vadK:vadK* (green bars) were grown in 7H9 media without (solid bars) or with 10 mM propionate (checkered bars). ATP was measured (methods) and the results presented are ± SEM for 2-4 independent biological replicates. Where n>2 biological replicates statistical significance is calculated using a two-way ANOVA with Dunnet's multiple-comparison test.

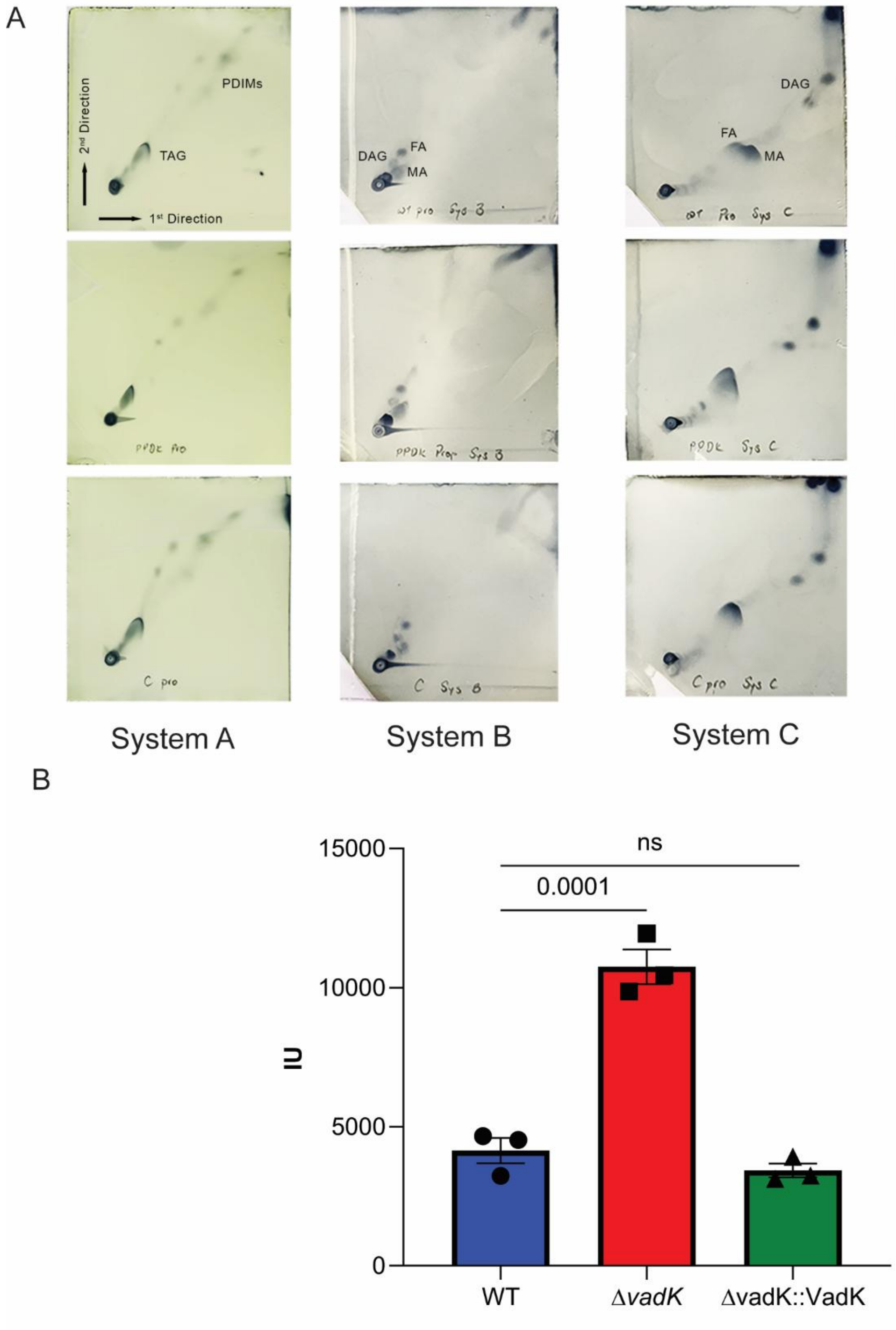

Appendix Figure S9. No significant changes in cell wall lipids in Mtb lacking *vadK*, except for sulphur-lipids. (A) 2D TLC analysis of apolar lipid fractions from Mtb strains grown in propionate. 1st and 2nd direction of runs are shown in a representative TLC (n=3) on the top left-hand corner. TAG; triacylated glycerol, PDIM; phthiocerol dimycocerosate, DAG; diacylated glycerol, TMM, trehalose monomycolate, TDM, trehalose dimycolate; FA, free fatty acid; MA, free mycolic acid;

SL1, sulfolipid 1 (B) Bar graph representing densitometric quantification of SL-1 from System D TLCs from Mtb strains (n=3 biological replicates). IU, grey pixel intensity unit.
