# Supplementary material for: Mycobacterium tuberculosis VadK is required for the regulation of the methylcitrate cycle and virulence
Source: EMBO Rep. 2026 Jun 11;27(14):4100–23. doi: 10.1038/s44319-026-00818-0 (PMC13400623; doi:10.1038/s44319-026-00818-0)
Supplement: Supplementary file 6 — Expanded View Figures [file 44319_2026_818_MOESM6_ESM.pdf]

Expanded View Figures

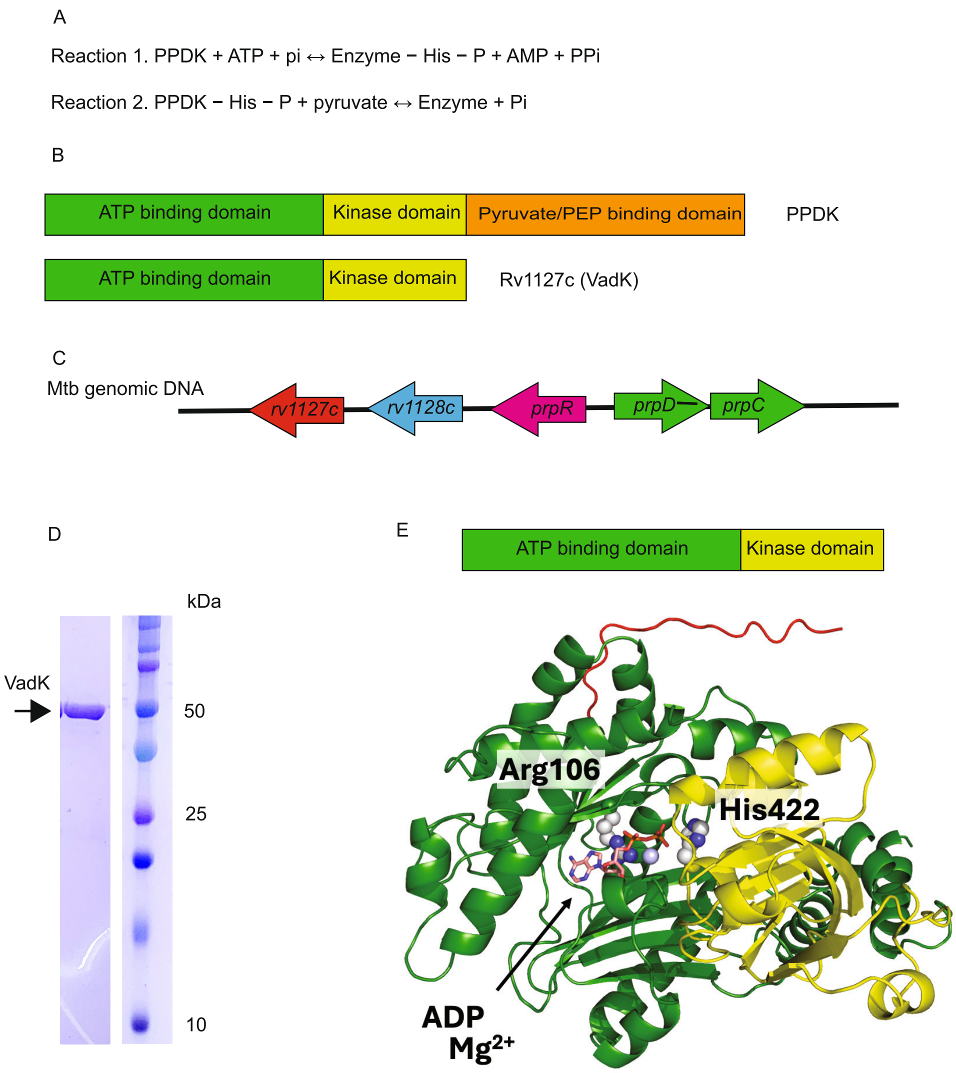

Figure EV1. Reaction of PPDK and its structural comparison with VadK.

(**A**) The two-step reaction of canonical PPDK. (**B**) VadK is missing the PPDK pyruvate binding domain. (**C**) *vadK* is in close proximity to genes of the methylcitrate cycle. (**C**) *Mtb* genomic location of *vadK*, (**D**) SDS-PAGE gel of purified VadK and (**E**) VadK complexed with ADP and Mg^{2+} structure prediction by AlphaFold3 (Abramson et al, 2024). The N-terminal 1-17 residues, ATP binding and kinase domains are coloured in red, green and yellow, respectively. ADP and Mg^{2+} are shown in stick (carbons, salmon) and sphere (light blue), respectively. The catalytic histidine, His422, and ATP-binding Arg106 are highlighted in spheres (carbons, white). Data Information: In (**E**), the structure was predicted with high confidence using AlphaFold3 (Abramson et al, 2024) with prediction statistics ipTM/pTM scores of 0.97/0.79 and visualised in PyMOL.

 © The Author(s)

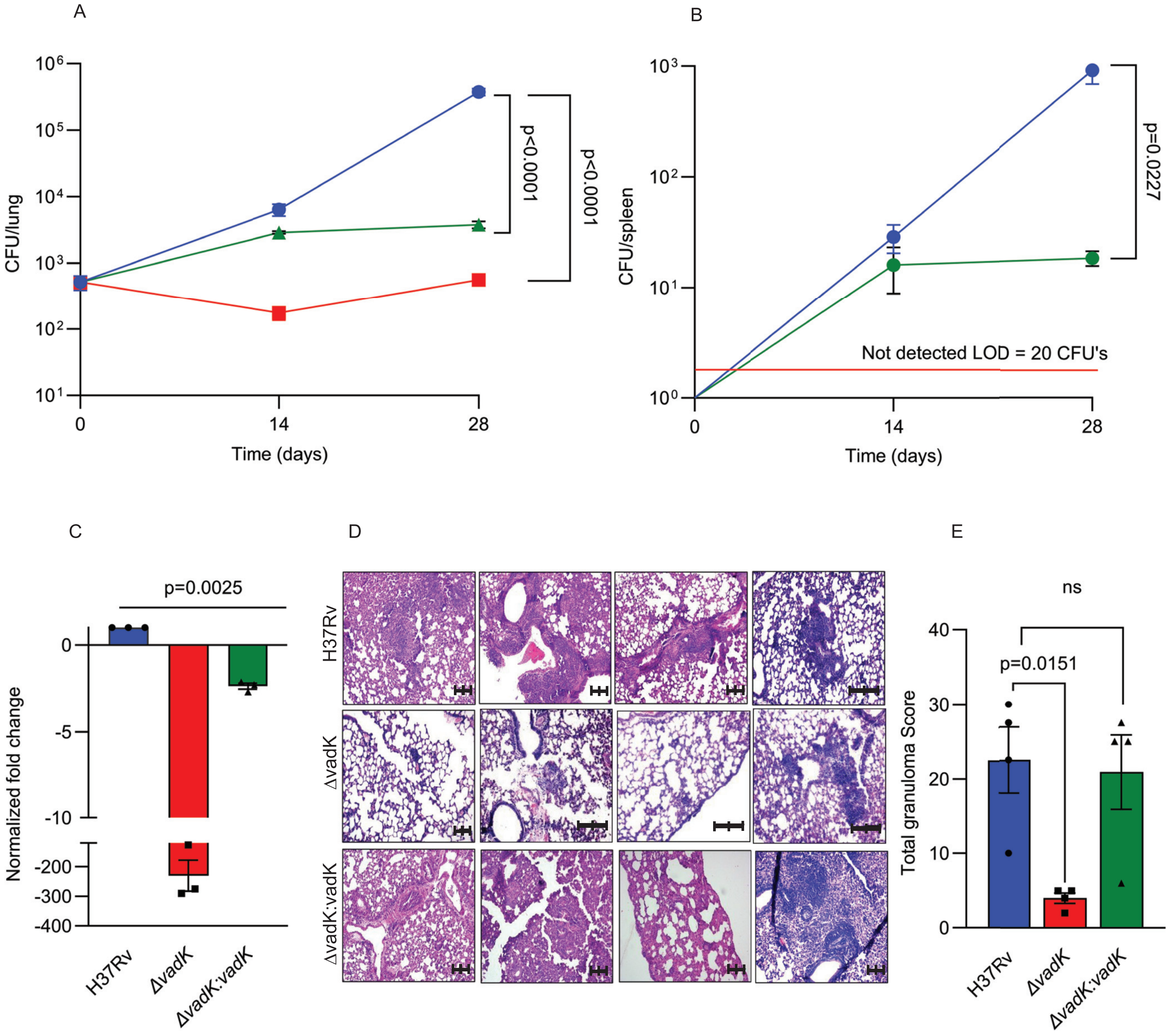

Figure EV2. VadK is required for *Mtb* to cause tuberculosis in BALB/c mice.

BALB/c mice ($n = 6$) were aerosol infected with WT (blue), *ΔvadK* (red) and *ΔvadK:vadK* (green) and the bacterial load in the (**A**) lungs and (**B**) spleen was measured at day 14 and day 28. *ΔvadK* was not detected in the spleen, and the limit of detection is 20 CFU's. (**C**) Total RNA was isolated from animal passaged logarithmically grown cells ($n = 3$) of WT H37Rv, *ΔvadK* (red) and *ΔvadK:vadK* (green) and expression of *vadK* was measured by real-time PCR. The results are from $n = 3$ biological replicates and are expressed as fold change as compared with WT expression ± SEM. (**D**) Lung sections were stained with HE after 28 days of infection and scored blindly by a pathologist using the method described (Kramnik and Beamer, 2016). The images show HE-stained (10 × magnification) from individual sections representative of four infected animals, and (**E**) the scores for animals ($n = 4$) in each group. Data information: In (**A–C**, **E**), data were presented as mean ± SEM. Comparison for determining statistical significance was made using an ordinary two-way ANOVA (Dunnett's multiple comparison test) for (**A**), an unpaired *t*-test with Welch's correction for (**B**, **C**) and an ordinary one-way ANOVA (Dunnett's multiple comparison test) for (**E**). Scale bars in (**D**): 100 µm.

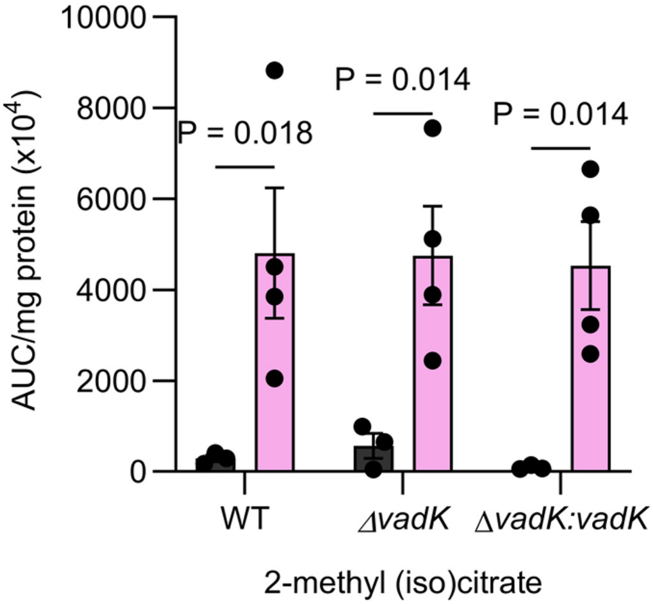

Figure EV3. Abundance of 2-methyl(iso)citrate.

MS measurements of intracellular 2-methyl(iso)citrate from *Mtb* strains grown in either Roisin's minimal media with cholesterol (black bars) or 7H9 with 20 mM propionate (pink bars) for 48 h. Abundances are shown as normalised AUC (Methods). Data information: Mean ± SEM ($n = 3$–4 biological replicates). Statistics was calculated using an unpaired two-tailed *t*-test with Welch's correction.

 © The Author(s)

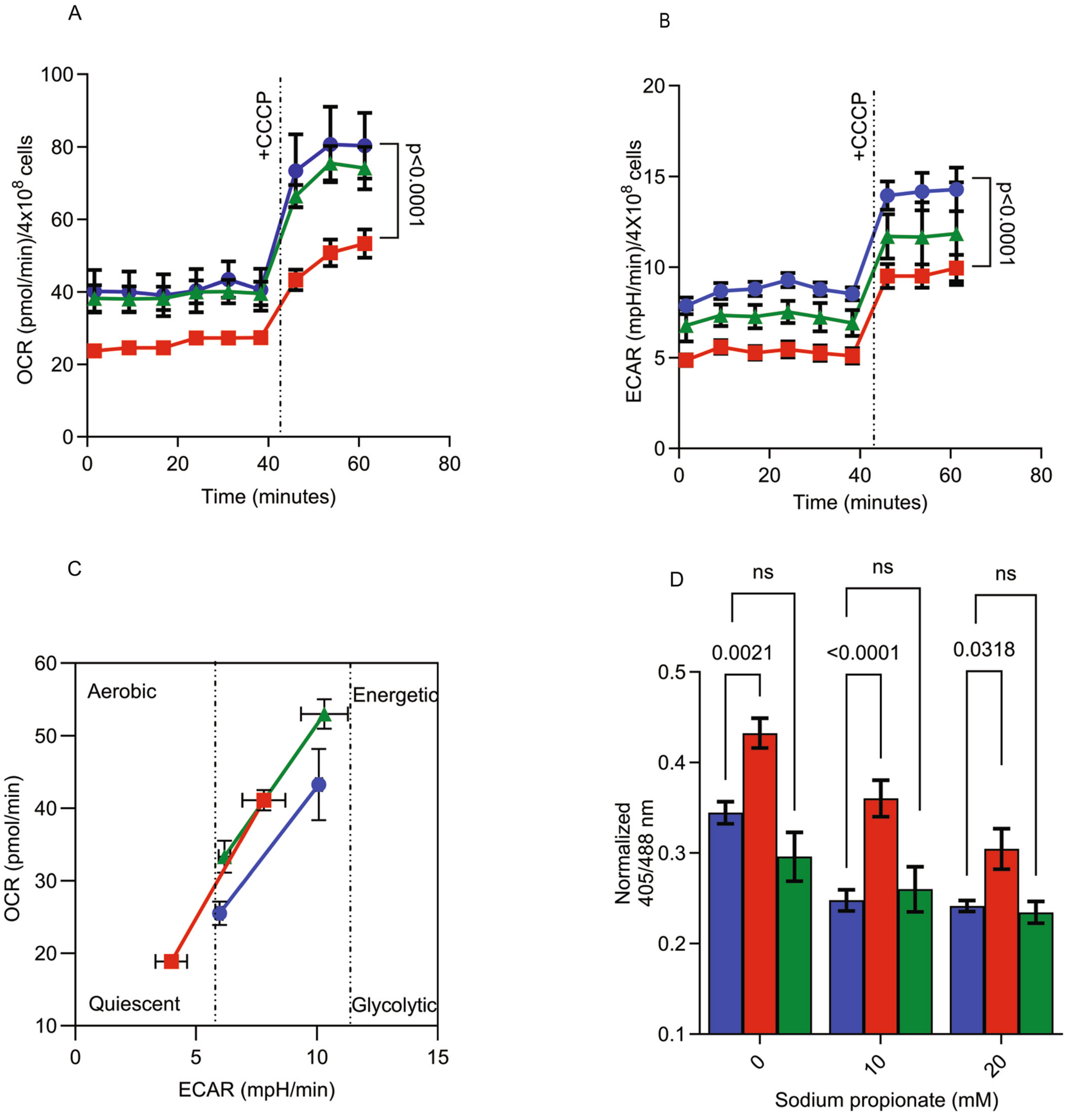

Figure EV4. VadK-deficient *Mtb* has reduced oxygen consumption rate (OCR), extracellular acidification rate (ECAR) and is more oxidised.

The metabolic potential of WT, *ΔvadK* and *ΔvadK:vadk* was assessed using the Agilent Seahorse XF cell energy phenotype test. Basal and stressed energy profiles were generated by measuring (**A**) oxygen consumption rate (OCR) and (**B**) extracellular acidification rate (ECAR), before and after treatment with the mitochondrial uncoupler CCCP. (**C**) The cell energy phenotype profile indicates that *ΔvadK* has low energetics in comparison to WT and complement. OCR and ECAR are normalised to colony-forming unit (CFU). (**D**) The mycothiol redox potential (EMSH) of WT (blue) and *ΔvadK* (red), *ΔvadK:vadK* (green) in 7H9 media with glycerol and propionate as indicated was determined by measuring Mrx1-roGFP2 biosensor ratio (405/488 nm) using flow cytometry. Data information: Mean ± SEM ($n = 9$ biological replicates). Statistical significance was calculated using a one-way ANOVA with Dunnett's multiple comparison test.

© The Author(s)

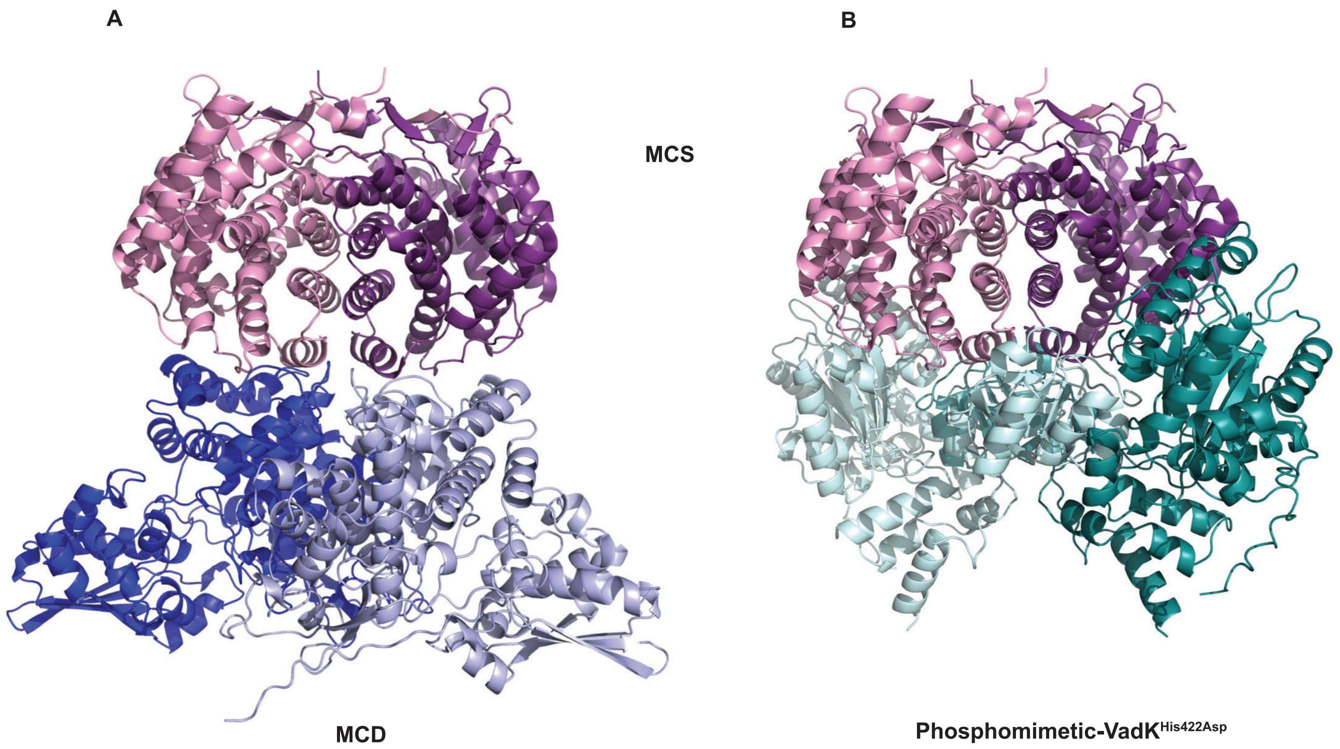

Figure EV5. Structure prediction of MCS/MCD and MCS and VadK.

(**A**) Cartoon representation of the AlphaFold3 (Abramson et al, 2024) predicted heterotetrameric complex of MCS (pink/purple) and MCD (blue/light blue) with medium confidence. (**B**) Cartoon representation of the predicted heterotetrameric complex of MCS (pink/purple) and phosphomimetic VadK^{His422Asp} (cyan/light cyan) with medium confidence. Data information: Models were generated using AlphaFold3 (Abramson et al, 2024) and visualised in PyMOL. Prediction statistics for (**A**) MCS/MCD ipTM/pTM scores is 0.41/0.55 and (**B**) MCS/VadK ipTM/pTM scores is 0.36/0.49.

 © The Author(s)
