# Supplementary material for: Mycobacterium tuberculosis VadK is required for the regulation of the methylcitrate cycle and virulence
Source: EMBO Rep. 2026 Jun 11;27(14):4100–23. doi: 10.1038/s44319-026-00818-0 (PMC13400623; doi:10.1038/s44319-026-00818-0)
Supplement: Supplementary file 2 — Peer Review File [file 44319_2026_818_MOESM2_ESM.pdf]

EMBO reports

Mycobacterium tuberculosis VadK is required for the regulation of the methylcitrate cycle and virulence

Jordan Pascoe, Jane Newcombe, Jessica Mendoza, Shalini Birua, Tom Mendum, Kushi Anand, Albel Singh, Amitava Sinha, Kadamba Papavinasasundaram, Apoorva Bhatt, Gerald Larrouy-Maumus, Amit Singh, Celia Goulding, and Dany Beste

Corresponding author(s): Dany Beste (d.beste@surrey.ac.uk)

Review Timeline:

Submission Date:	14th Nov 25
Editorial Decision:	8th Dec 25
Revision Received:	10th Mar 26
Editorial Decision:	14th Apr 26
Revision Received:	15th May 26
Accepted:	22nd May 26

Editor: Achim Breiling

Transaction Report:

(Note: With the exception of the correction of typographical or spelling errors that could be a source of ambiguity, letters and reports are not edited. Depending on transfer agreements, referee reports obtained elsewhere may or may not be included in this compilation. Referee reports are anonymous unless the Referee chooses to sign their reports.)

Dear Dr. Beste,

Thank you for the submission of your manuscript to EMBO reports. I have now received reports from the three referees that were asked to evaluate your study, which can be found at the end of this email. As you will see, the referees think that these findings are of interest. However, they have several comments, concerns, and suggestions, indicating that a major revision of the manuscript is necessary to allow publication of the study in EMBO reports. As the reports are below, and all the referee concerns need to be addressed, I will not detail them here.

Given the constructive referee comments, I would thus like to invite you to revise your manuscript with the understanding that the concerns of the referees must be addressed in the revised manuscript and/or in a detailed point-by-point response. Acceptance of your manuscript will depend on a positive outcome of a second round of review. It is EMBO reports policy to allow a single round of revision only and acceptance of the manuscript will therefore depend on the completeness of your responses included in the next, final version of the manuscript.

Revised manuscripts should be submitted within three months of a request for revision. Please contact me to discuss the revision (also by video chat) if you have questions or comments regarding the revision, or should you need additional time.

When submitting your revised manuscript, please also carefully review the instructions that follow below.

PLEASE NOTE THAT upon resubmission revised manuscripts are subjected to an initial quality control prior to exposition to re-review. Upon failure in the initial quality control, the manuscripts are sent back to the authors, which may lead to delays. Frequent reasons for such a failure are the lack of the data availability section (please see below) and the presence of statistics based on n=2 (the authors are then asked to present scatter plots or provide more data points).

When submitting your revised manuscript, we will require:

1) a .docx formatted version of the final manuscript text (including legends for main figures, EV figures and tables), but without the figures included. Figure legends should be compiled at the end of the manuscript text.

2) individual production quality figure files as .eps, .tif, .jpg (one file per figure), of main figures and EV figures. Please upload these as separate, individual files upon re-submission.

The Expanded View format, which will be displayed in the main HTML of the paper in a collapsible format, has replaced the Supplementary information. You can submit up to 6 images as Expanded View. Please follow the nomenclature Figure EV1, Figure EV2 etc. The figure legend for these should be included in the main manuscript document file in a section called Expanded View Figure Legends after the main Figure Legends section. Additional Supplementary material should be supplied as a single pdf file labeled Appendix. The Appendix should have page numbers and needs to include a table of content on the first page (with page numbers) and legends for all content. Please follow the nomenclature Appendix Figure Sx, Appendix Table Sx etc. throughout the text, and also label the figures and tables according to this nomenclature.

For more details, please refer to our guide to authors:
https://link.springer.com/journal/44319/submission-guidelines#cms-Revised-submissions

Please consult our guide for figure preparation:
https://media.springernature.com/original/springer-cms/rest/v1/content/27825798/data/v1

3) a .docx formatted letter INCLUDING the reviewers' reports and your detailed point-by-point responses to their comments. As part of the EMBO Press transparent editorial process, the point-by-point response is part of the Review Process File (RPF), which will be published alongside your paper.

4) a complete author checklist, which you can download from our author guidelines (https://link.springer.com/journal/44319/submission-guidelines#cms-Revised-submissions). Please insert page numbers in the checklist to indicate where the requested information can be found in the manuscript. The completed author checklist will also be part of the RPF.

Please also follow our guidelines for the use of living organisms, and the respective reporting guidelines:
https://link.springer.com/partners/embo-press/editorial-policies#Animal%20and%20human%20studies

5) that primary datasets produced in this study (e.g. RNA-seq, ChIP-seq, structural and array data) are deposited in an appropriate public database. If no primary datasets have been deposited, please also state this in a dedicated section (e.g. 'No primary datasets have been generated and deposited'), see below.

See also: https://link.springer.com/partners/embo-press/editorial-policies#Data%20deposition

Please remember to provide a reviewer password if the datasets are not yet public.

The accession numbers and database should be listed in a formal "Data Availability" section that follows the model below. This is now mandatory (like the COI statement). Please note that the Data Availability Section is restricted to new primary data that are part of this study. This section is mandatory. As indicated above, if no primary datasets have been deposited, please state this in this section

Data availability

The datasets produced in this study are available in the following databases:

- RNA-Seq data: Gene Expression Omnibus GSE46843 (https://www.ncbi.nlm.nih.gov/geo/query/acc.cgi?acc=GSE46843)
- [data type]: [name of the resource] [accession number/identifier/doi] ([URL or identifiers.org/DATABASE:ACCESSION])

*** Note - All links should resolve to a page where the data can be accessed. ***

See also: https://link.springer.com/partners/embo-press/editorial-policies#Data%20availability%20statement

Moreover, I have these editorial requests:

6) We now request the publication of original source data with the aim of making primary data more accessible and transparent to the reader. You will receive a separate email with instructions for providing source data with your revised manuscript, including information how to upload and organize the files.

7) Our journal encourages inclusion of *data citations in the reference list* to directly cite datasets that were re-used and obtained from public databases. Data citations in the article text are distinct from normal bibliographical citations and should directly link to the database records from which the data can be accessed. In the main text, data citations are formatted as follows: "Data ref: Smith et al, 2001" or "Data ref: NCBI Sequence Read Archive PRJNA342805, 2017". In the Reference list, data citations must be labeled with "[DATASET]". A data reference must provide the database name, accession number/identifiers and a resolvable link to the landing page from which the data can be accessed at the end of the reference. Further instructions are available at: https://link.springer.com/journal/44319/submission-guidelines#cms-Reference-guidelines

8) Regarding data quantification and statistics, please make sure that the number "n" for how many independent experiments were performed, their nature (biological versus technical replicates), the bars and error bars (e.g. SEM, SD) and the test used to calculate p-values is indicated in the respective figure legends (also for EV and Appendix figures). Please also check that all the p-values are explained in the legend, and that these fit to those shown in the figure. Please provide statistical testing where applicable. Please avoid the phrase 'independent experiment' but clearly state if these were biological or technical replicates. Please also indicate (e.g. with n.s.) if testing was performed, but the differences are not significant. In case n=2, please show the data as separate datapoints without error bars and statistics. See also: https://link.springer.com/journal/44319/submission-guidelines#cms-Figure-and-data-presentation

If n<5, please show single datapoints for diagrams.

9) Please add scale bars of similar style and thickness to all microscopic images, using clearly visible black or white bars (depending on the background). Please place these in the lower right corner of the images themselves. Please do not write on or near the bars in the image but define the size in the respective figure legend.

10) Please also note our reference format:
https://link.springer.com/journal/44319/submission-guidelines#cms-Reference-guidelines

11) We updated our journal's competing interests policy in January 2022 and request authors to consider both actual and perceived competing interests. Please review the policy https://www.embopress.org/competing-interests and update your competing interests if necessary. Please name this section 'Disclosure and Competing Interests Statement' and put it after the Acknowledgements section.
See: https://link.springer.com/partners/embo-press/editorial-policies#Competing%20interest%20disclosures

12) We now use CRediT to specify the contributions of each author in the journal submission system. CRediT replaces the author contribution section. Please use the free text box to provide more detailed descriptions and do NOT provide your final manuscript text file with an author contributions section. See also our guide to authors (section 'Author contributions'): https://link.springer.com/journal/44319/submission-guidelines#cms-Revised-submissions

13) All Materials and Methods need to be described in the main text using our 'Structured Methods' format, which is required for

all research articles. According to this format, the Methods section should include a Reagents and Tools Table (listing key reagents, experimental models, software, and relevant equipment and including their sources and relevant identifiers), uploaded as separate file, and a Methods section in which we encourage the authors to describe their methods using a step-by-step protocol format with bullet points, to facilitate the adoption of the methodologies across labs. More information on how to adhere to this format as well as downloadable templates (.doc) for the Reagents and Tools Table can be found in our author guidelines (section 'Structured Methods'):

https://link.springer.com/journal/44319/submission-guidelines#cms-Manuscript-organisation-and-formatting

14) Please order the manuscript sections like this, using only these names:
Title page - Abstract (max. 175 words) - Keywords - Introduction - Results - Discussion - Methods - Data availability section - Acknowledgements (please include here all the funding information) - Disclosure and Competing Interests Statement - References - Figure legends - Expanded View Figure legends

15) Please make sure that all the funding information is also entered into the online submission system and that it is complete and similar to the one in the acknowledgement section of the manuscript text file.

We would also welcome the submission of cover suggestions, or motifs to be used by our Graphics Illustrator in designing a cover.

As part of the EMBO publication's Transparent Editorial Process, EMBO reports publishes online a Review Process File (RPF) to accompany accepted manuscripts. This File will be published in conjunction with your paper and will include the referee reports, your point-by-point response and all pertinent correspondence relating to the manuscript.

You are able to opt out of this by letting the editorial office know (emboreports@embo.org). If you do opt out, the Review Process File link will point to the following statement: "No Review Process File is available with this article, as the authors have chosen not to make the review process public in this case."

I look forward to seeing a revised form of your manuscript when it is ready.

Yours sincerely,

Achim Breiling
Senior Editor
EMBO Reports

Referee #1:

The manuscript entitled "Mycobacterium tuberculosis VadK required for regulation of the methylcitrate 2 cycle and virulence"by Pascoe et al represents a compelling and well-supported study demonstrating that Mycobacterium tuberculosis Rv1127c, long annotated as pyruvate phosphate dikinase, has evolved into a distinct regulatory enzyme that functions as a histidine kinase in pathogenic mycobacteria. Through a combination of biochemical assays, genetic analysis, and systems-level metabolic profiling, the authors provide clear evidence that this enzyme-renamed Virulence Associated DiKinase (VadK)-autophosphorylates and plays an essential role in Mtb metabolism and virulence. The work convincingly shows that VadK is required for the utilization of host-relevant carbon sources and for establishing infection in murine models. Particularly notable is the integration of protein-protein interaction studies with ^13C-metabolic flux analysis, demonstrating that VadK modulates methylcitrate cycle flux and that dysregulated flux becomes growth limiting. Together, these findings establish VadK as a key regulatory node linking central carbon metabolism with virulence, reveal an unappreciated facet of metabolic regulation in Mtb pathogenesis, and highlight VadK as a plausible therapeutic target.

Overall, the claims made in the abstract are strongly supported by the presented experimental evidence, and the study represents an important contribution to the field of mycobacterial physiology and pathogenesis.
Two points to consider:

1. The inability to observe full complementation with the integrated plasmid expressing the vadK gene is interesting. Was this integrated in the L5 attachment site? This needs to be explicitly added as it may suggest that chromosome location is paly a role in geen expression. As the authors could have used 6 other different integration vectors for complementation, a specification of the vector abd its reference should be included. As an alternative explabation, if they used a vector that retained the integrase, the complementing vector could have been lost in subpopulations of transformants in vivo. I am not asking for them to repeat the mouse in vitro complementation, but it shpild be noted what vector was used.
2. My first reaction when I failed to see full complementation was perhaps the authors had inadvertently selected for sub populations of cells that had lost their ability to be virulent due to the loss of PDIM as previously described by [Mulholland et al,

(2024) Nature Microbiol.]. Their lipid analysis clearly shows this is not the case, but it is worthwhile to mention that study as loss of PDIM is frequently seen for many Mtb strains when grown extensively in Middlebrook media and can be selected against with the additon of low levels of propionate - consistent with the groups model that Mtb lives on a mixed carbon diet in vivo.

Referee #2:

In their manuscript entitled "Mycobacterium tuberculosis VadK required for regulation of the methylcitrate cycle and virulence," Pascoe, Newcombe et al. further define the role of VadK, a protein they previously implicated in Mtb central carbon metabolism (anaplerotic node) and in virulence using THP-1 macrophages (Basu et al. 2018). Earlier metabolomic work from the same group (Borah et al. 2021) indicated that VadK may not function within the anaplerotic node, consistent with their observation that VadK is not a canonical PPDK enzyme because it lacks the C terminal domain required for binding PEP/pyruvate, a feature typically necessary for participation in the anaplerotic node.

In the present study, the authors confirm a role for VadK in Mtb virulence using two mouse models and show that VadK undergoes auto phosphorylation, a modification that appears essential for its function. Building on their prior finding that a vadK deletion mutant (ΔvadK) fails to grow in the presence of cholesterol or propionate (Basu et al. 2018), they hypothesize that VadK contributes to regulation of the methylcitrate cycle (MCC), which enables Mtb to detoxify propionate and cholesterol derived propionate. Consistent with this hypothesis, the vadK gene is located in the same locus as MCC enzymes PrpC and PrpD, VadK interacts physically with both proteins, and loss of VadK increases PrpC activity, suggesting that VadK negatively regulates the MCC, particularly PrpC. The authors also show that carbon flux from cholesterol and propionate is altered in the ΔvadK mutant and that this phenotype is associated with increased ATP levels and oxidative stress, reduced oxygen consumption, and impaired utilization of other carbon sources (glycerol/pyruvate) that require a "reverse" MCC for full assimilation.

The lack of a human VadK orthologue supports its potential as a drug target and underscores the significance of this work. The virulence defects of the ΔvadK mutant are convincingly demonstrated, as are the in vitro kinase assays and the requirement for VadK phosphorylation to complement the ΔvadK growth defect in propionate. However, the metabolomic data, particularly in propionate grown cultures (Fig. 4), do not clearly support the proposed central role of VadK in propionate assimilation, and re analysis of these datasets may strengthen the authors' model. In addition, the manuscript does not present or discuss a clear mechanistic framework explaining how VadK regulates the MCC; for example, how VadK-PrpC/PrpD interactions and VadK phosphorylation modulate enzyme activity, or how both ΔvadK and ΔprpC mutants share a propionate growth defect if VadK acts as an inhibitor of PrpC. The emphasis on growth defects of the ΔvadK mutant on carbon sources other than cholesterol and propionate further rises doubts regarding the specificity of VadK's role in propionate/cholesterol assimilation and MCC regulation.

Major comments:
1. In Figure 3, the authors focus on fluxes and therefore display only metabolites containing one or two 13C labels; however, it would be very informative to also report the AUC/mg protein of the unlabeled (fully 12C) metabolites, as already done for 2 methyl(isocitrate) in Fig. S8. Providing total pool sizes for all metabolites, at least in a supplementary figure, would allow assessment of whether reduced cholesterol assimilation into specific intermediates is accompanied by a decrease in their overall abundance and could help better explain the ΔvadK growth defect and any pathway specific roles of VadK. Statistical comparisons between the ΔvadK mutant and the complemented strain appear to be missing in Figure 3 and should be added. Furthermore, clarification on whether pyruvate and, specifically, 13C labeled pyruvate derived from 13C cholesterol were detected would be valuable, as such data could reveal whether the ΔvadK strain shows altered capacity to derive pyruvate from cholesterol, which is toxic to this mutant.
2. The flux changes observed in the ΔvadK mutant with 13C cholesterol (Fig. 3) are largely absent when 13C propionate is used (Fig. 4), particularly for MCC intermediates, which appears inconsistent with the statement in lines 681-682 that loss of vadK enhances propionate flux through the MCC. For instance, the ΔvadK mutant seems to channel more cholesterol into methyl(isocitrate) in Fig. 3, whereas a comparable increase is not evident with 13C propionate in Fig. 4, despite the mutant being growth inhibited by both propionate and cholesterol. This discrepancy raises questions about the contribution of increased methyl(isocitrate) flux to the ΔvadK growth defect; the authors should clarify and discuss these differences, including whether they could arise from distinct labeling patterns (direct 13C propionate versus 13C pyruvate derived from cholesterol). In addition, Figure 4 normalizes metabolite levels to 100%, which may obscure differences in absolute pool sizes between ΔvadK and wild type; providing total metabolite pools, even in supplementary material, and specifying the reference used for the statistical tests in each condition would greatly enhance interpretability
3. The data support a model in which VadK inhibits MCC activity (Fig. 4B), possibly through direct interaction with PrpC and PrpD (Table 1), and the authors also show that VadK function depends on its phosphorylation state (Fig. 2 and Fig. S6). Nevertheless, the manuscript does not discuss how VadK-PrpC or VadK-PrpD interactions might alter enzyme function, how VadK phosphorylation modulates these interactions, or how these mechanisms together influence PrpC activity as inferred from Figure 4B. The authors are encouraged to propose and discuss a mechanistic model addressing these points; predictive structural analyses, such as AlphaFold based modeling of the VadK-PrpC complex, could provide testable hypotheses and strengthen the mechanistic insights of the study.
4. In line 721, the authors state that the 13C data demonstrate reduced flux through the pentose phosphate pathway (PPP), yet

no PPP intermediates are directly measured. Although reduced carbon incorporation into histidine is compatible with diminished PPP flux, it could also arise from other, as yet uncharacterized effects on histidine biosynthesis in the ΔvadK mutant. The claim regarding reduced PPP flux should therefore be softened or more cautiously worded.
5. In Figure 5, the ΔvadK mutant displays a growth defect when glycerol or pyruvate is provided as the sole carbon source, which the authors attribute to impaired reverse MCC activity. This observation, however, also suggests a broader carbon source dependency of the ΔvadK growth defect and may weaken the argument for a specific role in propionate/cholesterol metabolism. The authors should consider providing growth curves for single carbon sources that either require or bypass the MCC and, where possible, include MCC deficient control strains (e.g., ΔprpC, ΔprpD, or ΔprpR) to support their interpretation.

Minor comments:
• Table 1: Please provide additional details on how the numerical values in the table were calculated.
• Lines 48-52: The text appears to classify bedaquiline, an ATP synthase inhibitor, as a central carbon metabolism inhibitor; it would be more accurate to describe bedaquiline as a respiratory inhibitor, consistent with current literature (e.g., PMID: 37235914).
• Line 150: Replace "10- or 20-mm" with "10- or 20-mM."
• Line 519: Replace "CD" with "circular dichroism (CD)."
• Figure 3: Add a clear label for the y axis (e.g., "AUC/mg protein"), consistent with the methods.
• Figures 3 and 4: Given the density of information, consider removing cofactors (NAD/FAD, ATP/ADP, water, CO22) and metabolites for which no data are presented (e.g., E4P, S7P, chorismate) from the pathway schematics to improve clarity.
• Figure 4: Ensure that all graphs explicitly indicate WT, ΔvadK, and ΔvadK::vadK and that the x axis has a descriptive title.
• Line 624: Replace "(Fig S9)" with "(Fig. S8)."
• Line 683: Remove the word "lyase."
• Line 738: Replace "which is accordance" with "which is in accordance.

Referee #3:

The manuscript "Mycobacterium tuberculosis VadK required for regulation of the methylcitrate cycle and virulence" authored by Pascoe and cols. characterizes the enzymatic function of the Rv1127c gene of Mycobacterium tuberculosis. Using biochemical, carbon flux, in vitro growth and animal infection assays, the authors identify the histidine kinase function of this protein and its potential involvement in central carbon metabolism.

Major comments:
- The structure of the manuscript is confusing, since it goes from in vivo data to in vitro data, but the opposite structure will improve the readability of the manuscript. I propose the following structure:
1-Phylogenetic analyses of Rv1127c (VadK) in Mycobacterium and related genera
2-Identification of the VadK deletion in the MTBC and related pathogenic mycobacteria (i. e. M. abscessus), but not in non-pathogenic mycobacteria (i. e. M. smegmatis). Please include here a sequence alignment of VadK orthologes to illustrate the phylogeny.
3-Confirmation of kinase activity of VadK in M. tuberculosis
4-Immunoprecipitation assays of VadK and interqaction with PrpC and PrpD
5-Carbon flux assays with the VadK mutant
6-Growt defects in propionate, and B12 rescue, of the VadK mutant
7-Infection assays in C3H and BALB/c mice

-The absence of a complete complementation in vivo is intriguing. The authors point to insufficient expression of VadK from the attB insertion site; however, they do not clarify whether they use the own vadK promoter, or an unrelated promoter contained in the complementing vector. An expression assay (qRT-PCR, protein expression, or similar) in wt, mutant and complemented strains is required to confirm VadK expression levels.
-Increased levels of SL in the VadK mutant could represent an interesting result due to the key role of this lipid in pathogenesis. However, I can hardly observe the SL band in TLCs (Fig S11A). In addition, the authors should clarify the number of experiments, since the SL expression is calculated without error bars (Fig S11B).
-Section "VadK does not transcriptionally regulate the MCC during propionate metabolism" (L544-L597). This paragraph is too long, considering that the results obtained do not have further implications for the manuscript. This section should be shortened to accommodate the main findings.
-Immunoprecipitation assays: it is surprising that the authors did not include the vadK mutant as control. Please clarify, or include this result.
-Figure 3: Increase the font sizes of the central diagram (central carbon metabolism).
-Figure 4: Increase the font sizes of the whole picture. Consider moving to supplementary figures.

Minor comments:
L27: "architecture of an organisms metabolic and regulatory networks", might refer to "architecture of an organism's metabolic and regulatory networks"?

L88: essential is a strong assumption considering that this gene was not identified as essential for in vitro growth; replace with required, or something similar.
L492: BALB/C BALB/c
L520: clarify the sentence "For these experiments, we didn't remove the His-tag." For example "For these experiments, we didn't remove the His-tag contained in the expression vector and used for protein purification."
L546-547: Rephrase the sentence "Bacterial histidine kinases typically act as two-component regulators that modulate gene expression." For example: "Histidine kinases in bacteria typically are constituents of two-component systems that modulate gene expression."
L596-597: Delete this sentence "and may represent non-specific associations through conserved nucleotide-binding interfaces rather than bona fide interactions." Unless the authors provide the appropriate control in immunoprecipitation experiments (vadK mutant), these proteins were identified as interacting partners.
L639: OR withour capital letters.
L741-742: Remove this sentence, or indicate that it was only observed in a single experiment.
L778: replace abscesses with abscessus.

Point by point response to reviewers

We thank you for your constructive and insightful comments on our manuscript. We are very pleased that Referee 1 agrees that our work establishes VadK as a key regulatory node linking central carbon metabolism with virulence. We also agree with Referee 2 that the lack of a human VadK orthologue supports its potential as a drug target and underscores the significance of this work. All of you provided valuable comments that have helped us to improve the clarity and impact of our manuscript, which we address point by point below and in the revised version.

Referee #1:

Overall, the claims made in the abstract are strongly supported by the presented experimental evidence, and the study represents an important contribution to the field of mycobacterial physiology and pathogenesis.

Reviewer comment. The inability to observe full complementation with the integrated plasmid expressing the vadK gene is interesting. Was this integrated in the L5 attachment site? This needs to be explicitly added as it may suggest that chromosome location plays a role in gene expression. As the authors could have used 6 other different integration vectors for complementation, a specification of the vector and its reference should be included. As an alternative explanation, if they used a vector that retained the integrase, the complementing vector could have been lost in subpopulations of transformants in vivo. I am not asking for them to repeat the mouse in vitro complementation, but it should be noted what vector was used.

Response. The complemented strain of *ΔvadK* (Née *Δppdk*) has been previously described in Basu et al. (2018); therefore, we did not redescribe it in full but have now added additional details below. The wild type *vadK* gene and native promoter was reintroduced into the *ΔvadK* strain using the pMV306 suicide vector, which integrates into the *att*B site of *M. tuberculosis*. We have also included qRT-PCR data to confirm that the complemented strain expresses *vadK*, albeit at slightly lower levels than the parental strain, consistent with the expected effects of gene relocation on expression. We have added this now in Fig. EV2C and added the text below in red.

Text added to manuscript from L170. *Although there was a significant difference between the growth of ΔvadK and ΔvadK:vadK, complementation was incomplete suggesting that expression of vadK from the chromosomally distant attB site where we re-inserted it into the genome using pMV306 under its native promoter was insufficient. (Basu et al., 2018). Consistent with this, qRT-PCR analysis of RNA derived from bacteria isolated from murine lungs (Fig. EV2C) confirmed that the complemented strain expresses VadK, albeit at lower levels than the parental strain. Further, the histopathology data (Fig. EV2D,E) showed that whilst mice infected with ΔvadK had significantly less tissue damage there were no significant differences between WT and ΔvadK:vadK infected mice providing further evidence that the phenotype of ΔvadK was due to deletion of vadK.*

Reviewer Comment. My first reaction when I failed to see full complementation was perhaps the authors had inadvertently selected for sub populations of cells that had lost their ability to be virulent due to the loss of PDIM as previously described by [Mulholland et al, (2024) Nature Microbiol]. Their lipid analysis clearly shows this is not the case, but it is worthwhile to mention that study as loss of PDIM is frequently seen for many Mtb strains when grown extensively in Middlebrook media and can be selected against with the addition of low levels of propionate - consistent with the groups model that Mtb lives on a mixed carbon diet in vivo.

Response. Fortunately, most of this work involved growth on propionate, which ensured that we maintained PDIM in our strains. We have added a sentence to the manuscript to emphasize this, including the Mulholland reference.

Text Added to manuscript from L400. *This result also confirmed that PDIM was maintained in our strains, driven by the frequent inclusion of propionate in our culture media, as recently described (Mulholland et al, 2024).*

Referee #2:

Reviewer summary. The lack of a human VadK orthologue supports its potential as a drug target and underscores the significance of this work. The virulence defects of the *ΔvadK* mutant are convincingly demonstrated, as are the in vitro kinase assays and the requirement for VadK phosphorylation to complement the *ΔvadK* growth defect in propionate.

Reviewer Comment. In Figure 3, the authors focus on fluxes and therefore display only metabolites containing one or two 13C labels; however, it would be very informative to also report the AUC/mg protein of the unlabeled (fully 12C) metabolites, as already done for 2 methyl(isocitrate) in Fig. S8. Providing total pool sizes for all metabolites, at least in a supplementary figure, would allow assessment of whether reduced cholesterol assimilation into specific intermediates is accompanied by a decrease in their overall abundance and could help better explain the ΔvadK growth defect and any pathway specific roles of VadK.

Response. Because our goal was to measure the changes in metabolic flux rather than pool sizes, which rarely correlate with flux, we did not initially include pool sizes other than for methyl(iso)citrate, that could become toxic if levels are elevated. We now include pool size data (Appendix Fig. S6), but do not observe any significant changes when the strains were grown in cholesterol.

Text Added to manuscript from L295. *However, 2-methyl(iso)citrate (Fig. EV3) and the levels of any other metabolite measured were not significantly different between the strains in these conditions (Appendix Fig. S6).*

Reviewer Comment. Statistical comparisons between the *ΔvadK* mutant and the complemented strain appear to be missing in Figure 3 and should be added.

Response. This has now been added.

Reviewer Comment. Furthermore, clarification on whether pyruvate and, specifically, ^{13}C-labeled pyruvate derived from ^{13}C cholesterol were detected would be valuable, as such data could reveal whether the *ΔvadK* strain shows altered capacity to derive pyruvate from cholesterol, which is toxic to this mutant.

Response. We have added this ^{13}C-labeling data from pyruvate into Fig. 3. Notably, ion suppression often results in a low pyruvate signal; therefore, alanine-directly derived from pyruvate-is used as a proxy, an approach that is well established in the field. Consistently, alanine also shows no significant difference in labelling.

Text Added to manuscript from L285. *There was no significant difference in 13C-labeling of pyruvate and alanine, indicating that ΔvadK can generate pyruvate from cholesterol at levels comparable to WT.*

Reviewer Comment. The flux changes observed in the Δ*vadK* mutant with ^{13}C cholesterol (Fig. 3) are largely absent when ^{13}C propionate is used (Fig. 4), particularly for MCC intermediates, which appears inconsistent with the statement in lines 681-682 that loss of vadK enhances propionate flux through the MCC. For instance, the Δ*vadK* mutant seems to channel more cholesterol into methyl(isocitrate) in Fig. 3, whereas a comparable increase is not evident with ^{13}C propionate in Fig. 4, despite the mutant being growth inhibited by both propionate and cholesterol. This discrepancy raises questions about the contribution of increased methyl(isocitrate) flux to the ΔvadK growth defect; the authors should clarify and discuss these differences, including whether they could arise from distinct labeling patterns (direct ^{13}C-propionate versus ^{13}C pyruvate derived from cholesterol).

Response. We agree that the magnitude of the flux changes differs between the two experiments presented in Figures 3 and 4. However, both datasets demonstrate a clear and significant increase in flux through methyl(iso)citrate and for the mirrored ^{13}C experiment into the downstream intermediates of the MCC (Figure 4). Whilst the presence of multiple carbon sources in the mirrored ^{13}C experiment complicates direct quantitative comparison with the cholesterol-only condition, Figure 4 unequivocally shows increased propionate flux through all the MCC intermediates.

We have revised this section to better emphasise this point and to address the reviewers' comments.

Text Added to manuscript from L325. The different media and ^{13}C labels used limit direct quantitative comparison between ^{13}C-experiments (Fig. 3,4) however there are notable differences worth discussion. Under cholesterol-only conditions, the elevated flux into 2-methyl(iso)citrate measured for *ΔvadK* (Fig. 3) was greater than that detected for ^{13}C-propionate (Fig. 4) but did not extend to other MCC intermediates, likely due to differences in flux through the glyoxylate shunt. This pattern differs from that measured for propionate, where we measured significantly increased ^{13}C-incorporation into 2-methyl(iso)citrate and MCC intermediates malate, aspartate (as a proxy for OAA) and succinate (Fig. 4). Together, these results demonstrated that loss of vadK results in increased flux through the MCC when Mtb is grown in either cholesterol or propionate.

Reviewer Comment. In addition, Figure 4 normalizes metabolite levels to 100%, which may obscure differences in absolute pool sizes between *ΔvadK* and wild type; providing total metabolite pools, even in supplementary material, and specifying the reference used for the statistical tests in each condition would greatly enhance interpretability

Response. The results shown in the previous version of Fig. 4 were not normalized, as the ^{13}C and ^{12}C fractions naturally sum to 100%. However, in response to the reviewers' comments and to help the readers better understand the data, we have now plotted just the ^{13}C labelling from glycerol and propionate in Figure 4. We have also added details of the statistical analyses used.

We have now included metabolite pool sizes in the publication (Appendix Fig. S7), which show that the relative levels of aspartate and alanine were reduced in *ΔvadK* compared with the parental strain. As these metabolites are directly derived from the intermediate's oxaloacetate and pyruvate, which are cycled through the MCC, these data provide further evidence that increased flux through the MCC reduces their production. The mutant also showed a relative reduction in glutamate, GABA, and serine. Text below has been added to the manuscript to emphasise these points.

Text Added to manuscript from L341. *However, we did detect a small but significant reduction in the relative levels of aspartate and alanine in ΔvadK compared with the parental strain (Appendix Fig. 7). As these amino acids are directly derived from OAA and pyruvate respectively, key intermediates of the MCC, these data provide further evidence of increased MCC flux in ΔvadK in propionate conditions limiting their production. The mutant also showed a small but significant relative reduction in pool size of glutamate, GABA, and serine as compared with the parental strain (Appendix Fig. 7).*

Reviewer Comment. The data support a model in which VadK inhibits MCC activity (Fig. 4B), possibly through direct interaction with PrpC and PrpD (Table 1), and the authors also show that VadK function depends on its phosphorylation state (Fig. 2 and Fig. S6). Nevertheless, the manuscript does not discuss how VadK-PrpC or VadK-PrpD interactions might alter enzyme function, how VadK phosphorylation modulates these interactions, or how these mechanisms together influence PrpC activity as inferred from Figure 4B. The authors are encouraged to propose and discuss a mechanistic model addressing these points; predictive structural analyses, such as AlphaFold based modeling of the VadK-PrpC complex, could provide testable hypotheses and strengthen the mechanistic insights of the study.

Response. We have now added the below text and accompanying Fig. EV5 for our current hypothesis of how VadK regulates the methylcitrate cycle.

Text Added to manuscript from L424. *Collectively, our data indicate that VadK regulates the methylcitrate cycle (MCC) by modulating the activity of MCS and/or MCD. Our pull-down assays indicated that this is modulated through direct interactions between VadK and the MCC enzymes. In addition to our AlphaFold3 structure of VadK (Fig. EV1E), we also predicted the structures of MCS and MCD and found that*

AlphaFold3 supports formation of a heterotetrameric MCS-MCD complex (Fig. EV5A). Furthermore, AlphaFold3 predicts that VadK can form a heterotetrameric complex with MCS and, notably, that phosphomimetic VadK^{His422Ala} binds MCS with higher predicted affinity than unphosphorylated VadK (Fig. EV5B).

Co-transcription of the genes encoding these MCC enzymes, prpC and prpD (Fig. EV1C), further supports the possibility that MCS and MCD form a functional complex (Griffin et al, 2011). Biologically, such an interaction could be advantageous for Mtb, as it would facilitate channelling of the toxic methylcitrate intermediate between enzyme active sites, thereby minimizing toxicity to the rest of the cell.

Based on these observations, we propose a working model in which VadK competes with MCD for binding to MCS, disrupting the putative MCS-MCD complex. Furthermore, binding of phosphorylated VadK to MCS modulates MCS activity. Future work will focus on validating these predictions through experimental determination of the protein complexes.

Reviewer Comment. In line 721, the authors state that the ^{13}C data demonstrate reduced flux through the pentose phosphate pathway (PPP), yet no PPP intermediates are directly measured. Although reduced carbon incorporation into histidine is compatible with diminished PPP flux, it could also arise from other, as yet uncharacterized effects on histidine biosynthesis in the *ΔvadK* mutant. The claim regarding reduced PPP flux should therefore be softened or more cautiously worded.

Response. Measurement of proteinogenic amino acids directly reports isotopic labelling in central metabolites, reflecting pathway activity, and underpins classical ^{13}C-Metabolic Flux Analysis (MFA). Histidine derives from ribose-5-phosphate in the pentose phosphate pathway, and its labelling is a well-established proxy for flux through this pathway, validated in multiple ^{13}C-MFA studies (e.g. Sauer, 2006; Zamboni et al., 2009 etc).

Reviewer Comment. In Figure 5, the *ΔvadK* mutant displays a growth defect when glycerol or pyruvate is provided as the sole carbon source, which the authors attribute to impaired reverse MCC activity. This observation, however, also suggests a broader carbon source dependency of the ΔvadK growth defect and may weaken the argument for a specific role in propionate/cholesterol metabolism. The authors should consider providing growth curves for single carbon sources that either require or bypass the MCC and, where possible, include MCC deficient control strains (e.g., ΔprpC, ΔprpD, or ΔprpR) to support their interpretation.

Response. The role of VadK is in modulating the MCC and therefore effects Mtb growth in several carbon sources not just cholesterol and propionate. The attenuated growth of *ΔVadK* in either glycerol or pyruvate is complemented by vitamin B12, directly linking the pyruvate and glycerol phenotypes to the methylcitrate cycle (MCC) rather than suggesting "a more general carbon source dependency". Previous studies have already assessed VadK and indeed MCC mutants under a broad range of conditions: *ΔvadK* (nee

Δppdk) in Basu et al. (2018) and *ΔprpC, ΔprpD*, and *ΔprpR* in multiple publications. Therefore, we do not see the merit of repeating these experiments.

Reviewer Comment. Table 1: Please provide additional details on how the numerical values in the table were calculated.

Response. We have now modified the description of how the numbers were generated. The numbers are the ratio of average peptide spectrum matches from VadKMtb grown in 7H9 + Prop versus 7H9 only.

Text Added to Table 1 legend. *Mtb transformed with a plasmid encoding VadK-FLAG under a constitutive promoter (VadKMtb) were grown in standard 7H9 media (7H9) and standard 7H9 media and propionate (7H9 + prop). VadKMtb was immunoprecipitated from whole-cell lysates and interacting proteins were identified by MS. Data were calculated from two biological replicates. Nonspecific binding peptides were removed by setting the filter of Peptide spectrum matches (PSMs) of each replicate to "≤1" in the WT control samples and "≥50" in "VadKMtb" samples. Hits were ranked in descending order based on ratio of average PSM's from VadKMtb grown in 7H9 + propionate versus 7H9. Raw data have been deposited to the ProteomeXchange Consortium via the PRIDE {Perez-Riverol, 2025 #1121} partner repository with the dataset identifier PXD073941.*

Reviewer Comment. Lines 48-52: The text appears to classify bedaquiline, an ATP synthase inhibitor, as a central carbon metabolism inhibitor; it would be more accurate to describe bedaquiline as a respiratory inhibitor, consistent with current literature (e.g., PMID: 37235914).

This point is largely moot, as bedaquiline directly targets ATP synthase, which is functionally intrinsically linked to metabolism by virtue of its central role in energy production. Nonetheless, we appreciate the reviewer's perspective and have clarified the text in the manuscript to make this connection explicit.

Text Added to manuscript from L78. *This connection is illustrated by bedaquiline, a clinically approved ATP synthase inhibitor, which disrupts energy production linked to CCM and exhibits potent bactericidal activity (Lakshmanan & Xavier, 2013).*

Reviewer Comment. Line 150: Replace "10- or 20-mm" with "10- or 20-mM."

Response. Done

Reviewer Comment. Line 519: Replace "CD" with "circular dichroism (CD)."

Response. Done

Reviewer Comment. Figure 3: Add a clear label for the y axis (e.g., "AUC/mg protein"), consistent with the methods.

Response. We have added the x-axis.

Reviewer Comment. Figures 3 and 4: Given the density of information, consider removing cofactors (NAD/FAD, ATP/ADP, water, CO22) and metabolites for which no

data are presented (e.g., E4P, S7P, chorismate) from the pathway schematics to improve clarity.

Response. We have simplified the metabolic map for clarity.

Reviewer Comment. Figure 4: Ensure that all graphs explicitly indicate WT, ΔvadK, and ΔvadK::vadK and that the x axis has a descriptive title.

Response. Done

Reviewer Comment. Line 624: Replace "(Fig S9)" with "(Fig. S8)."

Reviewer Comment. Line 683: Remove the word "lyase."

Response. Done

Reviewer Comment. Line 738: Replace "which is accordance" with "which is in accordance.

Response. Done

Referee #3:

Reviewer Comment. The structure of the manuscript is confusing, since it goes from in vivo data to in vitro data, but the opposite structure will improve the readability of the manuscript. I propose the following structure:

Response. The other two reviewers did not express any concern regarding our decision to begin by highlighting what we consider a central finding, namely, that a truncated gene remains essential for *M. tuberculosis* virulence. We intentionally present the in vivo data first, as it provides the most physiologically relevant context and logically frames the interpretation of the subsequent in vitro experiments.

Reviewer Comment. Please include here a sequence alignment of VadK orthologues to illustrate the phylogeny.

Response. Included in the raw data files for Figure 5.

Reviewer Comment. The absence of a complete complementation in vivo is intriguing. The authors point to insufficient expression of VadK from the attB insertion site; however, they do not clarify whether they use the own vadK promoter, or an unrelated promoter contained in the complementing vector. An expression assay (qRT-PCR, protein expression, or similar) in wt, mutant and complemented strains is required to confirm VadK expression levels.

Response. Please see response to referee 1 above.

Reviewer Comment. Increased levels of SL in the VadK mutant could represent an interesting result due to the key role of this lipid in pathogenesis. However, I can hardly observe the SL band in TLCs (Fig S11A). In addition, the authors should clarify the number of experiments, since the SL expression is calculated without error bars (Fig S11B).

Response. We have now provided a new figure with increased amount of apolar lipid loading showing the sulfolipids clearly. The TLC shown in Appendix Figure S9A is representative of three TLCs. The densitometric data was used to generate the bar graph shown in Appendix Figure S9B.

Reviewer Comment. Section "VadK does not transcriptionally regulate the MCC during propionate metabolism" (L544-L597). This paragraph is too long, considering that the results obtained do not have further implications for the manuscript. This section should be shortened to accommodate the main findings.

Response. We agree with this point, as the section does not substantially contribute to the main narrative of the manuscript, and we have therefore considerably shortened it (L247-258).

Reviewer Comment. Immunoprecipitation assays: it is surprising that the authors did not include the vadK mutant as control. Please clarify, or include this result.

Response. The KO strain was not used as a control for the pulldown experiments. Rather, WT lysate, that contains low levels of native VadK served as the negative control to define background binding arising from non-specific interactions with the beads and antibody, which represents standard practice for such experiments. This control effectively captures proteins that are non-specifically pulled down under the experimental conditions. Additionally, the KO strain is unable to grow on propionate, precluding its use as a control even if it were otherwise informative.

Reviewer Comment. Figure 3: Increase the font sizes of the central diagram (central carbon metabolism).

Response. Done

Reviewer Comment. Figure 4: Increase the font sizes of the whole picture. Consider moving to supplementary figures.

Response. We consider this to be an important figure and therefore do not want to move to supplementary

Reviewer Comment. L27: "architecture of an organisms metabolic and regulatory networks", might refer to "architecture of an organism's metabolic and regulatory networks"?

Response. Done

Reviewer Comment. L88: essential is a strong assumption considering that this gene was not identified as essential for in vitro growth; replace with required, or something similar.

Response. We are slightly confused by this point. Gene essentiality for many genes, and particularly for metabolic genes, is fundamentally conditional upon the specific environmental and nutritional context in which it is assessed. Accordingly, any measured essentiality denotes essentiality only under the experimental conditions

tested and should not be interpreted as an absolute property. The original Sassetti "essential for in vitro growth" experiments referenced by the reviewer were conducted when *M. tuberculosis* was grown in vitro on Middlebrook 7H10 medium. As demonstrated in subsequent studies from the Sassetti laboratory and other groups, many genes that are essential for in vivo growth or survival are distinct from those identified as essential under these in vitro conditions.

Reviewer Comment. L492: BALB/C □ BALB/c

Response. Done

Reviewer Comment. L520: clarify the sentence "For these experiments, we didn't remove the His-tag." For example, "For these experiments, we didn't remove the His-tag contained in the expression vector and used for protein purification."

Response. We have changed this.

Modified text added to the manuscript from L209. *For these experiments the His-tag used for purification was not removed.*

Reviewer Comment. L546-547: Rephrase the sentence "Bacterial histidine kinases typically act as two-component regulators that modulate gene expression." For example: "Histidine kinases in bacteria typically are constituents of two-component systems that modulate gene expression."

Modified text Added to manuscript from L236. *Bacterial histidine kinases are typically part of two-component regulators that modulate gene expression.*

Reviewer Comment. L596-597: Delete this sentence "and may represent non-specific associations through conserved nucleotide-binding interfaces rather than bona fide interactions." Unless the authors provide the appropriate control in immunoprecipitation experiments (vadK mutant), these proteins were identified as interacting partners.

See answer to response above.

Reviewer Comment. L639: OR without capital letters.

Response. Done

Reviewer Comment. L741-742: Remove this sentence, or indicate that it was only observed in a single experiment.

Response. We have now presented results from triplicated experiments.

Dear Dr. Beste,

Thank you for the submission of your revised manuscript to our editorial offices. I have now received the reports from the two referees that were asked to re-evaluate the study, you will find below. As you will see, the referees now fully support the publication of the study in EMBO reports.

Before we can proceed with formal acceptance, I have the editorial requests below I ask you to address in a final revised manuscript. Please also provide a final p-b-p-response to the editorial requests.

Editorial requests:

- I suggest this slightly modified and complete title:
Mycobacterium tuberculosis VadK is required for the regulation of the methylcitrate cycle and virulence

Please make sure this is used similarly in all places (submission system, manuscript text file and Appendix). Please also add this to the title page of the Appendix (Appendic for ... - followed by the title). Moreover, please add page numbers to the Appendix ToC.

- Please order the manuscript sections like this, using only these names:
Title page - Abstract - Keywords - Introduction - Results - Discussion - Methods - Data availability section - Acknowledgements - Disclosure and Competing Interests Statement - References - Figure legends - Expanded View Figure legends

- We now use CRediT to specify the contributions of each author in the journal submission system. CRediT replaces the author contribution section. Please use the free text box to provide more detailed descriptions and do NOT provide your final manuscript text file with an author contributions section. See also our guide to authors (section 'Author contributions'): https://link.springer.com/journal/44319/submission-guidelines#cms-Revised-submissions

- Please provide a fully completed author checklist including the manuscript number and responses in cells D81-D84.

- Please check again that the number "n" for how many independent experiments were performed, their nature (biological versus technical replicates), the bars and error bars (e.g. SEM, SD) and the test used to calculate p-values is indicated in the respective figure legends (main and EV figures). Please also check that all the p-values are explained in the legend, and that these fit to those shown in the figure. Please provide statistical testing where applicable. Please avoid the phrase 'independent experiment' but clearly state if these were biological or technical replicates. Please also indicate (e.g. with n.s.) if testing was performed, but the differences are not significant. In case n=2, please show the data as separate datapoints without error bars and statistics. See also:

https://link.springer.com/journal/44319/submission-guidelines#cms-Figure-and-data-presentation

If n<5, please show single datapoints for diagrams.

- Please add direct links for the deposited datasets in the data availability section.

- Please add scale bars of similar style and thickness to all microscopic images, using clearly visible black or white bars (depending on the background). Please place these in the lower right corner of the images themselves. Please do not write on or near the bars in the image but define the size in the respective figure legend. Presently, scale bars are too small and have text nearby (Fig. 1C) or are missing (Fig. EV2D).

- Please make sure that each figure panel is called out separately and that the panels are called out sequentially. The correct callout for Appendix figures is 'Appendix Figure Sx) (not Fig. SXy) and the dataset needs to be named and called out as 'Dataset EV1' (not Appendix Supplementary Dataset 1). Please correct or update these callouts or file names.

- Please place Table 1 between the main and EV figure legends.

- Please make sure that all the funding information is also entered into the online submission system and that it is complete and similar to the one in the acknowledgement section of the manuscript text file. Presently, the grant P200A240034 is missing in the submission system. Please check.

In addition, I would need from you uploaded separately:
- a short, two-sentence summary of the manuscript (not more than 35 words).
- two to four short (!) bullet points highlighting the key findings of your study (two lines each).
- a schematic summary figure as separate file that provides a sketch of the major findings (not a data image) in jpeg or tiff format

(with the exact width of 550 pixels and the exact height of 300 pixels) that can be used as a visual synopsis on our website.

I look forward to seeing the further revised version of your manuscript when it is ready. Please let me know if you have questions regarding the revision.

Best,

Achim Breiling
Senior Editor
EMBO Reports

Referee #2:

The authors addressed my previous concerns and I have no additional requests.

Referee #3:

I would like to thank the authors for their time and effort when revising the manuscript and responding to the raised reviewers' comments.

The revised version shows clear improvements, as several of the issues raised in the initial review have been addressed in a satisfactory manner. However, some points remain partially resolved and could have benefited from a more complete response.

That said, in my opinion, the manuscript has improved sufficiently and now meets the standards required for publication in EMBO reports. On this basis, I support its acceptance.

All minor editorial requests have been addressed by the authors.

2nd Revision - Editorial Decision **22nd May 2026**

Dr. Dany Beste
University of Surrey
Microbial Sciences
Stag Hill
Guildford, Surrey GU2 7XH
United Kingdom

Dear Dr. Beste,

I am very pleased to accept your manuscript for publication in the next available issue of EMBO reports. Thank you for your contribution to our journal.

Your manuscript will be processed for publication by EMBO Press. It will be copy edited and you will receive page proofs prior to publication. Please note that you will be contacted by Springer Nature Author Services to complete licensing and payment information.

You may qualify for financial assistance for your publication charges - either via a Springer Nature fully open access agreement or an EMBO initiative. Check your eligibility: https://link.springer.com/journal/44319/how-to-publish-with-us

Should you be planning a Press Release on your article, please notify the production team via this form in order to coordinate publication and release dates: https://forms.cloud.microsoft/e/eH4A8JVP6g

If you have any questions, please do not hesitate to contact the Editorial Office. Thank you for your contribution to EMBO Reports.

Yours sincerely,

Achim Breiling
Senior Editor
EMBO Reports

>>> Please note that it is EMBO Reports policy for the transcript of the editorial process (containing referee reports and your response letter) to be published as an online supplement to each paper. If you do NOT want this, you will need to inform the Editorial Office via email immediately. More information is available here: https://link.springer.com/partners/embo-press/editorial-policies#Peer%20review

EMBO Press Author Checklist

Corresponding Author Name: Dany Beste
Journal Submitted to: EMBO Reports
Manuscript Number: EMBOR-2025-63088

USEFUL LINKS FOR COMPLETING THIS FORM
The EMBO Journal - Author Guidelines
EMBO Reports - Author Guidelines
Molecular Systems Biology - Author Guidelines
EMBO Molecular Medicine - Author Guidelines

Reporting Checklist for Life Science Articles (updated January
This checklist is adapted from Materials Design Analysis Reporting (MDAR) Checklist for Authors. MDAR establishes a minimum set of requirements in transparent reporting in the life sciences (see Statement of Task: 10.31222/osf.io/9sm4x). Please follow the journal's guidelines in preparing your
Please note that a copy of this checklist will be published alongside your article.

Abridged guidelines for figures
1. Data
The data shown in figures should satisfy the following conditions:
- the data were obtained and processed according to the field's best practice and are presented to reflect the results of the experiments in an accurate and unbiased manner.
- ideally, figure panels should include only measurements that are directly comparable to each other and obtained with the same assay.
- plots include clearly labeled error bars for independent experiments and sample sizes. Unless justified, error bars should not be shown for technical
- if n<5, the individual data points from each experiment should be plotted. Any statistical test employed should be justified.
- Source Data should be included to report the data underlying figures according to the guidelines set out in the authorship guidelines on Data

2. Captions
Each figure caption should contain the following information, for each panel where they are relevant:
- a specification of the experimental system investigated (eg cell line, species name).
- the assay(s) and method(s) used to carry out the reported observations and measurements.
- an explicit mention of the biological and chemical entity(ies) that are being measured.
- an explicit mention of the biological and chemical entity(ies) that are altered/varied/perturbed in a controlled manner.
- the exact sample size (n) for each experimental group/condition, given as a number, not a range;
- a description of the sample collection allowing the reader to understand whether the samples represent technical or biological replicates (including how many animals, litters, cultures, etc.).
- a statement of how many times the experiment shown was independently replicated in the laboratory.
- definitions of statistical methods and measures:
  - common tests, such as t-test (please specify whether paired vs. unpaired), simple χ2 tests, Wilcoxon and Mann-Whitney tests, can be unambiguously identified by name only, but more complex techniques should be described in the methods section;
  - are tests one-sided or two-sided?
  - are there adjustments for multiple comparisons?
  - exact statistical test results, e.g., P values = x but not P values < x;
  - definition of 'center values' as median or average;
  - definition of error bars as s.d. or s.e.m.

Please complete ALL of the questions below.
Select "Not Applicable" only when the requested information is not relevant for your study.

Materials

Newly Created Materials	Information included in the manuscript?	In which section is the information available? (Reagents and Tools Table, Materials and Methods, Figures, Data Availability Section)
New materials and reagents need to be available; do any restrictions apply?	Not Applicable	

Antibodies	Information included in the manuscript?	In which section is the information available? (Reagents and Tools Table, Materials and Methods, Figures, Data Availability Section)
For **antibodies** provide the following information:<br>- Commercial antibodies: RRID (if possible) or supplier name, catalogue number and or/clone number<br>- Non-commercial: RRID or citation	Yes	Reagents and tools table and Materials and Methods

DNA and RNA sequences	Information included in the manuscript?	In which section is the information available? (Reagents and Tools Table, Materials and Methods, Figures, Data Availability Section)
**Short novel DNA or RNA including primers, probes**: provide the sequences.	Yes	Reagents and Tools table/Materials and Methods

Cell materials	Information included in the manuscript?	In which section is the information available? (Reagents and Tools Table, Materials and Methods, Figures, Data Availability Section)
**Cell lines:** Provide species information, strain. Provide accession number in repository **OR** supplier name, catalog number, clone number, and/**OR** RRID.	Not Applicable	
**Primary cultures:** Provide species, strain, sex of origin, genetic modification status.	Not Applicable	
Report if the cell lines were recently **authenticated** (e.g., by STR profiling) and tested for mycoplasma contamination.	Not Applicable	

Experimental animals	Information included in the manuscript?	In which section is the information available? (Reagents and Tools Table, Materials and Methods, Figures, Data Availability Section)
**Laboratory animals or Model organisms:** Provide species, strain, sex, age, genetic modification status. Provide accession number in repository **OR** supplier name, catalog number, clone number, **OR** RRID.	Yes	Reagents and tools table
**Animal observed in or captured from the field:** Provide species, sex, and age where possible.	Not Applicable	
Please detail **housing and husbandry conditions**.	Yes	Materials and methods

Plants and microbes	Information included in the manuscript?	In which section is the information available? (Reagents and Tools Table, Materials and Methods, Figures, Data Availability Section)
**Plants:** provide species and strain, ecotype and cultivar where relevant, unique accession number if available, and source (including location for collected wild specimens).	Not Applicable	
**Microbes:** provide species and strain, unique accession number if available, and source.	Yes	Reagent and tools table

Human research participants	Information included in the manuscript?	In which section is the information available? (Reagents and Tools Table, Materials and Methods, Figures, Data Availability Section)
If collected and within the bounds of privacy constraints report on age, sex and gender or ethnicity for all study participants.	Not Applicable	

Core facilities	Information included in the manuscript?	In which section is the information available? (Reagents and Tools Table, Materials and Methods, Figures, Data Availability Section)
If your work benefited from core facilities, was their service mentioned in the acknowledgments section?	Not Applicable	

Not Applicable

Design

Study protocol	Information included in the manuscript?	In which section is the information available? (Reagents and Tools Table, Materials and Methods, Figures, Data Availability Section)
If study protocol has been **pre-registered, provide DOI in the manuscript**. For clinical trials, provide the trial registration number **OR** cite DOI.	Not Applicable	
Report the **clinical trial registration number** (at ClinicalTrials.gov or equivalent), where applicable.	Not Applicable	

Laboratory protocol	Information included in the manuscript?	In which section is the information available? (Reagents and Tools Table, Materials and Methods, Figures, Data Availability Section)
Provide DOI OR other citation details if **external detailed step-by-step protocols** are available.	Not Applicable	

Experimental study design and statistics	Information included in the manuscript?	In which section is the information available? (Reagents and Tools Table, Materials and Methods, Figures, Data Availability Section)
Include a statement about **sample size** estimate even if no statistical methods were used.	Yes	Materials and Methods, Figure legends, data availability
Were any steps taken to minimize the effects of subjective bias when allocating animals/samples to treatment (e.g. **randomization procedure**)? If yes, have they been described?	Yes	Materials and Methods
Include a statement about **blinding** even if no blinding was done.	Yes	Materials and Methods
Describe **inclusion/exclusion criteria** if samples or animals were excluded from the analysis. Were the criteria pre-established?<br><br>If sample or data points were omitted from analysis, report if this was due to attrition or intentional exclusion and provide justification.	Not Applicable	
For every figure, are **statistical tests** justified as appropriate? Do the data meet the assumptions of the tests (e.g., normal distribution)? Describe any methods used to assess it. Is there an estimate of variation within each group of data? Is the variance similar between the groups that are being statistically compared?	Yes	

Sample definition and in-laboratory replication	Information included in the manuscript?	In which section is the information available? (Reagents and Tools Table, Materials and Methods, Figures, Data Availability Section)
In the figure legends: state number of times the experiment was **replicated** in laboratory.	Yes	Figure legends/data availability
In the figure legends: define whether data describe **technical or biological replicates**.	Yes	Figure legends/data availability

Ethics

Ethics	Information included in the manuscript?	In which section is the information available? (Reagents and Tools Table, Materials and Methods, Figures, Data Availability Section)
Studies involving **human participants**: State details of **authority granting ethics approval** (IRB or equivalent committee(s), provide reference number for approval.	Not Applicable	
Studies involving **human participants**: Include a statement confirming that **informed consent** was obtained from all subjects and that the experiments conformed to the principles set out in the WMA Declaration of Helsinki and the Department of Health and Human Services Belmont Report.	Not Applicable	
Studies involving **human participants:** For publication of **patient photos**, include a statement confirming that consent to publish was obtained.	Not Applicable	
Studies involving experimental **animals**: State details of **authority granting ethics approval** (IRB or equivalent committee(s), provide reference number for approval. Include a statement of compliance with ethical regulations.	Yes	Materials and methods
Studies involving **specimen and field samples:** State if relevant **permits** obtained, provide details of authority approving study; if none were required, explain why.	Not Applicable	

Dual Use Research of Concern (DURC)	Information included in the manuscript?	In which section is the information available? (Reagents and Tools Table, Materials and Methods, Figures, Data Availability Section)
Could your study fall under dual use research restrictions? Please check biosecurity documents and list of **select agents and toxins** (CDC): https://www.selectagents.gov/sat/list.htm	Not Applicable	
If you used a select agent, is the security level of the lab appropriate and reported in the manuscript?	Not Applicable	
If a study is subject to dual use research of concern regulations, is the name of the **authority granting approval and reference number** for the regulatory approval provided in the manuscript?	Not Applicable	

Reporting

The MDAR framework recommends adoption of discipline-specific guidelines, established and endorsed through community initiatives. Journals have their own policy about requiring specific guidelines and recommendations to complement MDAR.

Adherence to community standards	Information included in the manuscript?	In which section is the information available? (Reagents and Tools Table, Materials and Methods, Figures, Data Availability Section)
State if relevant guidelines or checklists (e.g., **ICMJE, MIBBI, ARRIVE, PRISMA**) have been followed or provided.	Not Applicable	
For **tumor marker prognostic studies,** we recommend that you follow the **REMARK** reporting guidelines (see link list at top right). See author guidelines, under 'Reporting Guidelines'. Please confirm you have followed these guidelines.	Not Applicable	
For **phase II and III randomized controlled trials**, please refer to the **CONSORT** flow diagram (see link list at top right) and submit the CONSORT checklist (see link list at top right) with your submission. See author guidelines, under 'Reporting Guidelines'. Please confirm you have submitted this list.	Not Applicable	

Data Availability

Data availability	Information included in the manuscript?	In which section is the information available? (Reagents and Tools Table, Materials and Methods, Figures, Data Availability Section)
Have **primary datasets** been deposited according to the journal's guidelines (see 'Data Deposition' section) and the respective accession numbers provided in the Data Availability Section?	Yes	Data availability section
Were **human clinical and genomic datasets** deposited in a public access-controlled repository in accordance to ethical obligations to the patients and to the applicable consent agreement?	Not Applicable	
Are **computational models** that are central and integral to a study available without restrictions in a machine-readable form? Were the relevant accession numbers or links provided?	Not Applicable	
If publicly available data were reused, provide the respective **data citations in the reference list**.	Not Applicable	
